# Spotted Fever Group *Rickettsia* Trigger Species-Specific Alterations in Macrophage Proteome Signatures with Different Impacts in Host Innate Inflammatory Responses

**DOI:** 10.1128/spectrum.00814-21

**Published:** 2021-12-22

**Authors:** Pedro Curto, Cátia Santa, Luísa Cortes, Bruno Manadas, Isaura Simões

**Affiliations:** a Center for Neuroscience and Cell Biology, University of Coimbra, Coimbra, Portugal; b Institute of Interdisciplinary Research, University of Coimbra, Coimbra, Portugal; National Institutes of Health

**Keywords:** SFG *Rickettsia*, obligate intracellular bacteria, macrophage permissiveness, immune evasion, host-pathogen interactions, pathogenicity, innate immunity, type I interferon responses, SWATH-MS/MS

## Abstract

The molecular details underlying differences in pathogenicity between *Rickettsia* species remain to be fully understood. Evidence points to macrophage permissiveness as a key mechanism in rickettsial virulence. Different studies have shown that several rickettsial species responsible for mild forms of rickettsioses can also escape macrophage-mediated killing mechanisms and establish a replicative niche within these cells. However, their manipulative capacity with respect to host cellular processes is far from being understood. A deeper understanding of the interplay between mildly pathogenic rickettsiae and macrophages and the commonalities and specificities of host responses to infection would illuminate differences in immune evasion mechanisms and pathogenicity. We used quantitative proteomics by sequential windowed data independent acquisition of the total high-resolution mass spectra with tandem mass spectrometry (SWATH-MS/MS) to profile alterations resulting from infection of THP-1 macrophages with three mildly pathogenic rickettsiae: Rickettsia parkeri, Rickettsia africae, and Rickettsia massiliae, all successfully proliferating in these cells. We show that all three species trigger different proteome signatures. Our results reveal a significant impact of infection on proteins categorized as type I interferon responses, which here included several components of the retinoic acid-inducible gene I (RIG-1)-like signaling pathway, mRNA splicing, and protein translation. Moreover, significant differences in protein content between infection conditions provide evidence for species-specific induced alterations. Indeed, we confirm distinct impacts on host inflammatory responses between species during infection, demonstrating that these species trigger different levels of beta interferon (IFN-β), differences in the bioavailability of the proinflammatory cytokine interleukin 1β (IL-1β), and differences in triggering of pyroptotic events. This work reveals novel aspects and exciting nuances of macrophage-*Rickettsia* interactions, adding additional layers of complexity between *Rickettsia* and host cells’ constant arms race for survival.

**IMPORTANCE** The incidence of diseases caused by Rickettsia has been increasing over the years. It has long been known that rickettsioses comprise diseases with a continuous spectrum of severity. There are highly pathogenic species causing diseases that are life threatening if untreated, others causing mild forms of the disease, and a third group for which no pathogenicity to humans has been described. These marked differences likely reflect distinct capacities for manipulation of host cell processes, with macrophage permissiveness emerging as a key virulence trait. However, what defines pathogenicity attributes among rickettsial species is far from being resolved. We demonstrate that the mildly pathogenic Rickettsia parkeri, Rickettsia africae, and Rickettsia massiliae, all successfully proliferating in macrophages, trigger different proteome signatures in these cells and differentially impact critical components of innate immune responses by inducing different levels of beta interferon (IFN-β) and interleukin 1β (IL-1β) and different timing of pyroptotic events during infection. Our work reveals novel nuances in rickettsia-macrophage interactions, offering new clues to understand *Rickettsia* pathogenicity.

## INTRODUCTION

The incidence of rickettsial infections has been increasing over the years across all geographic regions (1). Moreover, the overall rise in temperature caused by global climate change has increased the prevalence and activity of ticks, which is also expected to raise the incidence of tick-borne diseases in humans, including rickettsial diseases (2–5). Rickettsioses are a group of diseases with a continuous spectrum of disease severity caused by different rickettsial species (6, 7). Among these species are Rickettsia rickettsii, Rickettsia prowazekii, Rickettsia conorii, and Rickettsia typhi, which are considered highly pathogenic and are responsible for life-threatening diseases in humans, with very high mortality rates if untreated (1, 8). On the other hand, species like Rickettsia montanensis and Rickettsia amblyommatis have been considered to have limited or no pathogenicity to humans and are mainly associated with asymptomatic or mild illness with seroconversion (1, 7, 9). In between are organisms like R. parkeri, R. africae, and R. massiliae (among many others), etiologic agents of non-life-threatening rickettsioses to which no death, to our knowledge, has ever been attributed (6, 7, 10–14). Although this spectrum of disease severity in rickettsioses has long been known, the molecular signatures underlying such drastic differences in pathogenicity are still to be fully elucidated. The need to better understand the molecular details that contribute to rickettsial pathogenesis is further bolstered by the identification of many rickettsial species whose pathogenicity to humans is still uncertain (1, 4, 15).

In several animal models of *Rickettsia* infection, bacteria have been found within macrophages, raising important questions about the biological role of these phagocytic cells in rickettsial pathogenesis (16, 17). The ability to avoid macrophage-mediated killing mechanisms and replicate, or at least survive, within the cells that are supposed to kill pathogens has been considered an essential part of what it means to be a pathogen, giving rise to the so-called “macrophage paradox” (18). We have previously reported that a drastic phenotypic difference between the highly pathogenic R. conorii and the nonpathogenic R. montanensis lies in their ability to survive and proliferate within macrophages (19). Moreover, a study performed by Engström and colleagues has shown that *ompB*-deficient R. parkeri bacteria, which proliferated in human dermal microvascular endothelial cells (HMECs) but could not replicate in mouse bone marrow-derived macrophages (BMDMs), were unable to colonize mouse organs and cause disease (20). Together, these results point toward macrophages as critical players during rickettsial infections, by either acting to eliminate the bacteria or, alternatively, succumbing to rickettsia colonization and allowing bacterial replication and systemic dissemination, likely through a “Trojan horse” mechanism (6, 21). Thus, macrophage permissiveness to infection is emerging as a key mechanism in rickettsial pathogenesis. This reinforces the need to deepen our understanding on how rickettsia species escape macrophage immune defenses and establish a replicative niche within these phagocytic cells. We have recently reported that the highly pathogenic R. conorii substantially modulates several macrophage signaling pathways, interfering with a wide range of host cellular processes, likely changing the cellular environment to better suit its survival and proliferation requirements (22, 23). More recently, the ability of R. parkeri to use outer membrane protein B (rOmpB) to protect the bacterial surface from ubiquitylation has been described as a strategy to promote bacterial survival in macrophages through evasion of autophagy (20). Also, studies in BMDMs have shown that R. parkeri takes advantage of inflammasome-mediated host cell death to antagonize the deleterious effect of type I interferon (IFN-I) during infection (24). These results illustrate the intensive research efforts currently ongoing to understand the strategies employed by rickettsiae to establish a replicative niche within macrophages. However, the global landscape of alterations induced within the host by rickettsial species responsible for mild rickettsioses and species-specific nuances in host responses to infection remains vastly unexplored.

High-throughput approaches have emerged as valuable tools to study the host-pathogen interface during infection (25–27). Here, we employed a high-throughput quantitative proteomics approach, sequential windowed acquisition of all theoretical mass spectra with tandem mass spectrometry (SWATH-MS/MS), to profile alterations of the proteome of THP-1 macrophages upon infection with three rickettsial species responsible for mild rickettsioses: R. parkeri (causing *R. parkeri* rickettsiosis), R. africae (agent of African tick bite fever), and R. massiliae (causing a yet-unnamed spotted fever). Our results revealed that all three species triggered substantial alterations in proteomic signatures upon infection, impacting several host cellular processes. The observed changes in various proteins associated with RNA splicing, protein translation, and type I interferon responses, including several components of the retinoic acid-inducible gene I (RIG-1)-like receptor pathway (RLR), reveal additional mechanisms targeted/impacted by these obligate intracellular bacteria during macrophage colonization. Moreover, significant differences in protein content observed between infection conditions are consistent with species-specific induced alterations. These findings suggest that different mildly pathogenic rickettsiae are likely differentially possessed of manipulative capacities to subvert macrophage-mediated killing mechanisms. We provide further evidence of this by showing that R. parkeri, R. africae, and R. massiliae elicit qualitatively and quantitatively distinct immune responses, differentially impacting important mediators, such as IFN-β, IL-1β, or pyroptosis.

The findings presented herein provide additional insights into the complex interactions at the host-*Rickettsia* interface and provide evidence of species-dependent nuances that offer new clues to understand differences in pathogenicity attributes among rickettsiae.

## RESULTS

### SFG *Rickettsia* species responsible for mild rickettsioses induce substantial alterations in the proteome of THP-1 macrophages during infection.

**FIG 1:**
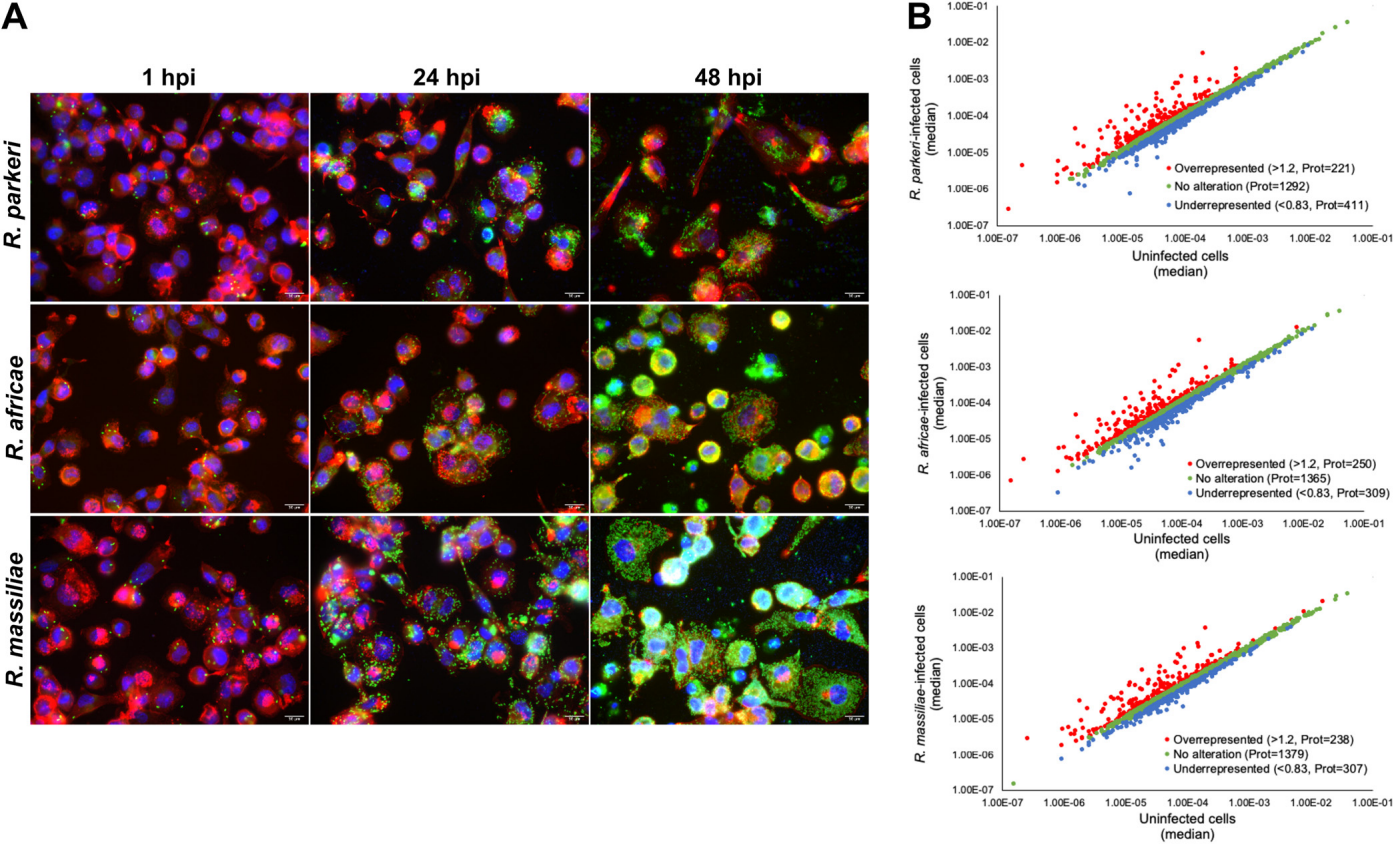
Global alterations in the proteome of THP-1 macrophages infected with R. parkeri, R. africae, and *R. massiliae*. (A) Immunofluorescence microscopy of THP-1 macrophages infected with *R. parkeri*, *R. africae*, and *R. massiliae* (MOI of 10) at 1 h, 24 h, and 48 h postinfection. Cells were stained with DAPI (blue) to stain host nuclei, phalloidin (red) to stain actin, and rabbit anti-*Rickettsia* polyclonal antibody NIH/RML I7198 followed by Alexa Fluor 488 (green) to stain *Rickettsia*. Scale bar = 10 μm. (B) Scatterplot representation of changes in protein abundance of THP-1 macrophages upon infection with *R. parkeri* (top), *R. africae* (middle), and *R. massiliae* (bottom) (at 24 hpi, MOI of 10). The 1,925 proteins that were confidently quantified in all 4 experimental conditions were plotted and considered altered when a change of at least 20% in abundance (fold change of ≤0.83 or ≥1.2) was observed between uninfected and infected conditions. Proteins that were considered to decrease, not change, or increase their abundance upon infection are represented in blue, green, and red, respectively. See also Table S1 in the supplemental material.

The ability of spotted fever group (SFG) *Rickettsia* species responsible for mild rickettsioses, namely, R. parkeri and R. africae, to establish a replicative niche within macrophages has been reported previously (20, 24, 28). However, the global landscape of strategies employed to subvert macrophage-mediated killing mechanisms and the nature of macrophage cellular processes modulated/manipulated during infection by these and other mildly pathogenic rickettsiae remain largely unknown. We herein used a high-throughput, label-free, quantitative proteomics approach (SWATH-MS/MS) to extensively characterize the responses of macrophages to infection by R. parkeri, R. africae, and R. massiliae, all responsible for milder forms of the disease. Using phorbol 12-myristate 13-acetate (PMA)-differentiated THP-1 macrophages as our model of study, we confirmed by quantitative PCR and immunofluorescence microscopy that the three rickettsial species established a replicative niche within these phagocytic cells (multiplicity of infection [MOI] of 10) (Fig. 1A; Fig. S1). Total protein extracts were then prepared from uninfected and infected THP-1 macrophages at 24 h postinfection (hpi) in a total of 4 biological replicates per experimental condition. The relative protein quantification was performed using liquid chromatography (LC)-SWATH-MS analysis, where a comprehensive library of 2,837 confidently identified proteins was created. A total of 1,925 proteins were confidently quantified in all samples. Proteins were considered altered when an alteration of at least 20% in abundance (fold change of ≤0.83 or ≥1.2) was observed between uninfected and infected conditions (23, 29, 30). Using these criteria, significant changes in the content of host proteins were observed upon infection with these rickettsial species. Specifically, we found alterations in the content of 632 (411 enriched and 221 reduced), 559 (309 enriched and 250 reduced), and 545 (307 enriched and 238 reduced) host proteins upon infection with *R. parkeri*, *R. africae*, and *R. massiliae*, respectively (Fig. 1B; Table S1).

### Infection of THP-1 macrophages with *R. parkeri*, *R. africae*, and *R. massiliae* impacts several host cellular functions.

**FIG 2:**
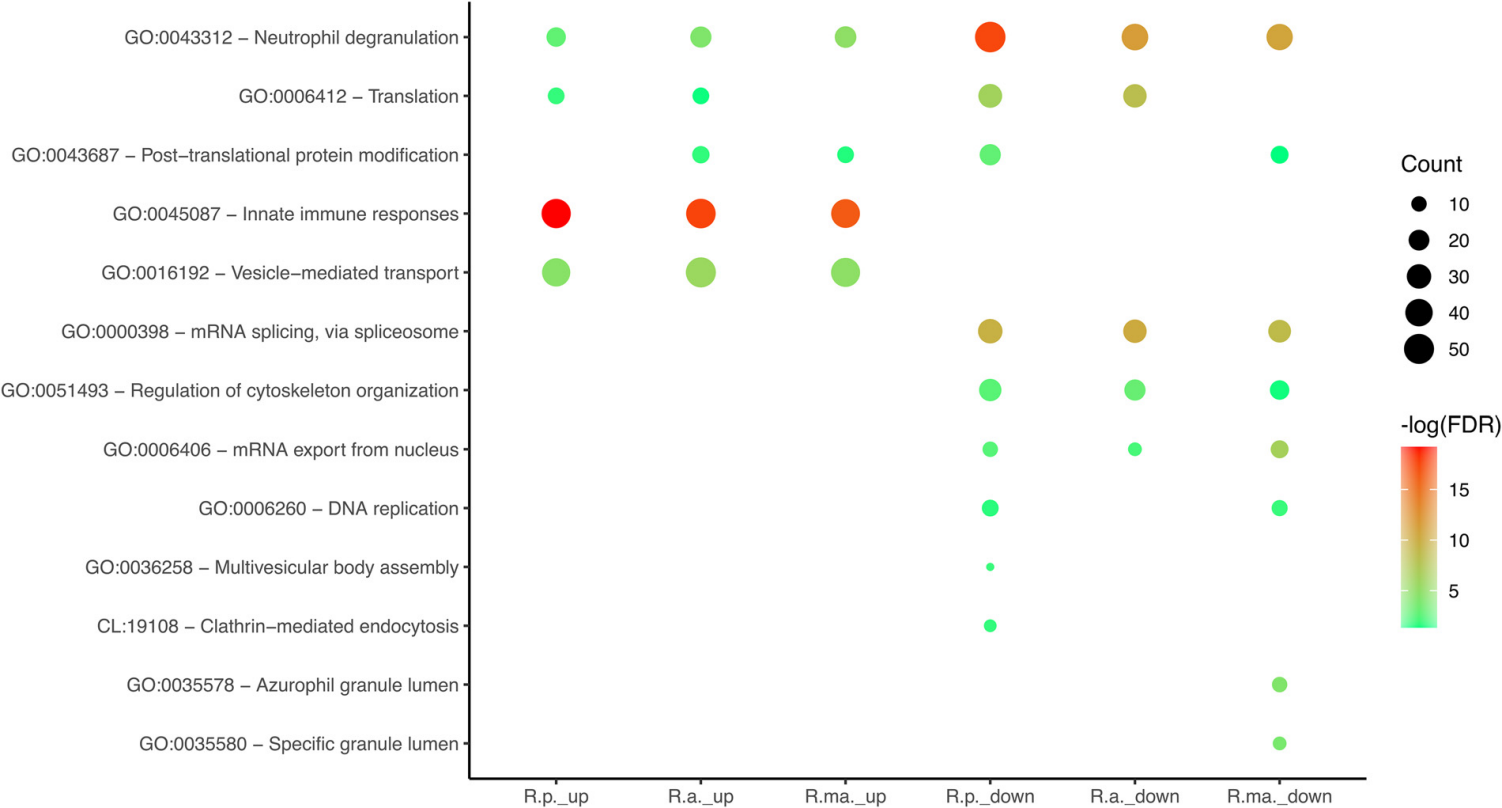
Gene ontology (GO) classification analysis of host proteins with altered abundance upon infection. Protein-protein interaction network cluster analysis for the host proteins with increased and decreased abundance upon infection with each rickettsial species was carried out with STRING 11.0 (http://string-db.org) using the highest confidence score (0.9) and MCL clustering with an inflation parameter of 3 to select the most representative and significant GO or local STRING network cluster (CL) terms (Fig. S2 to S4). GO Enrichment Plot function in Image GP (http://www.ehbio.com/ImageGP/) was then used to integrate and represent the Gene Ontology analysis. Analysis of the 221, 250, and 238 host proteins with increased abundance upon infection with R. parkeri, R. africae, and R. massiliae is represented by R.p._up, R.a._up, and R.ma._up, respectively. Analysis of the 411, 309, and 307 host proteins with decreased abundance upon infection with *R. parkeri*, *R. africae*, and *R. massiliae* is represented by R.p._down, R.a._down, and R.ma._down, respectively. The dot size indicates the number of host proteins with altered abundance for the specific infection condition of each GO or CL categorization. The dot color indicates the significance of the enrichment [−log_10_(FDR-corrected *P* value)].

**FIG 3:**
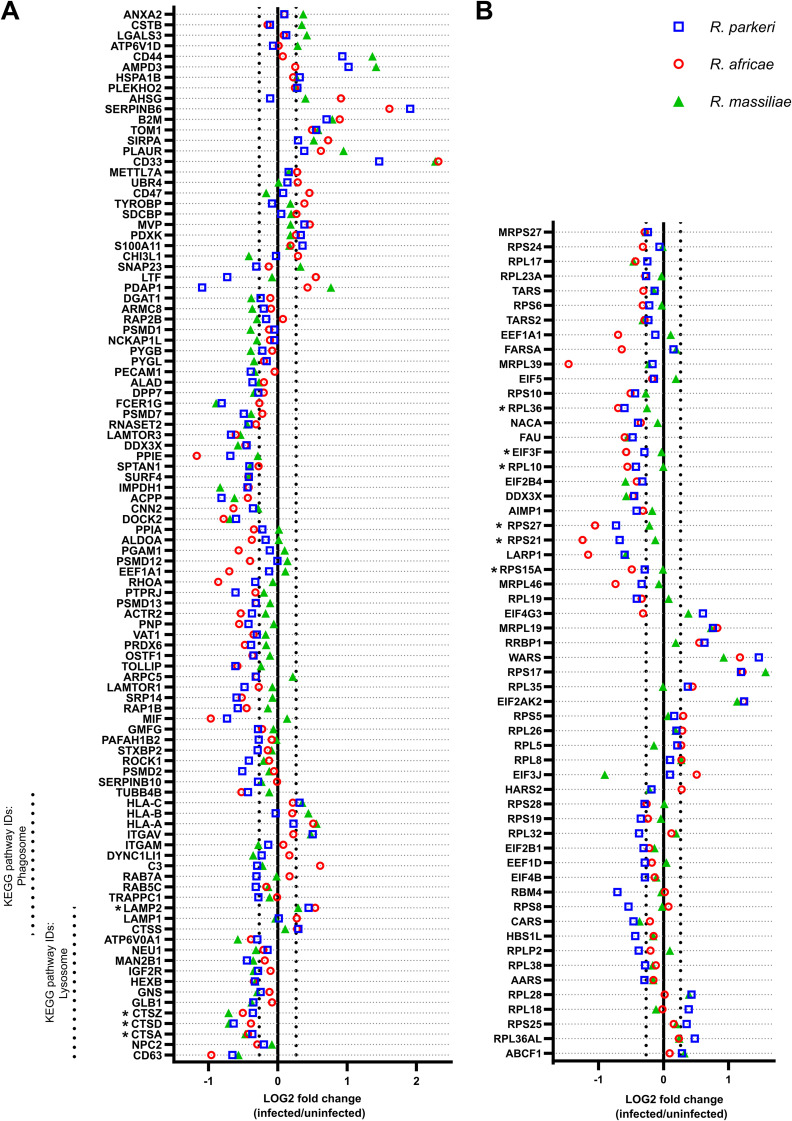
Rickettsial species responsible for mild rickettsioses affect several biological processes during infection of THP-1 macrophages. (A and B) Combined list of the individual gene names (and respective log_2_ fold change values) categorized with the GO terms neutrophil degranulation (GO:0043312) (A) and translation (GO:0006412) (B) that display altered abundance in at least one infection condition. Colored circles represent individual proteins in THP-1 macrophages infected with R. parkeri (blue), R. africae (red), and R. massiliae (green). Gene names associated with an asterisk highlight proteins described in the text. Detailed information can be found in Table S2.

**FIG 4:**
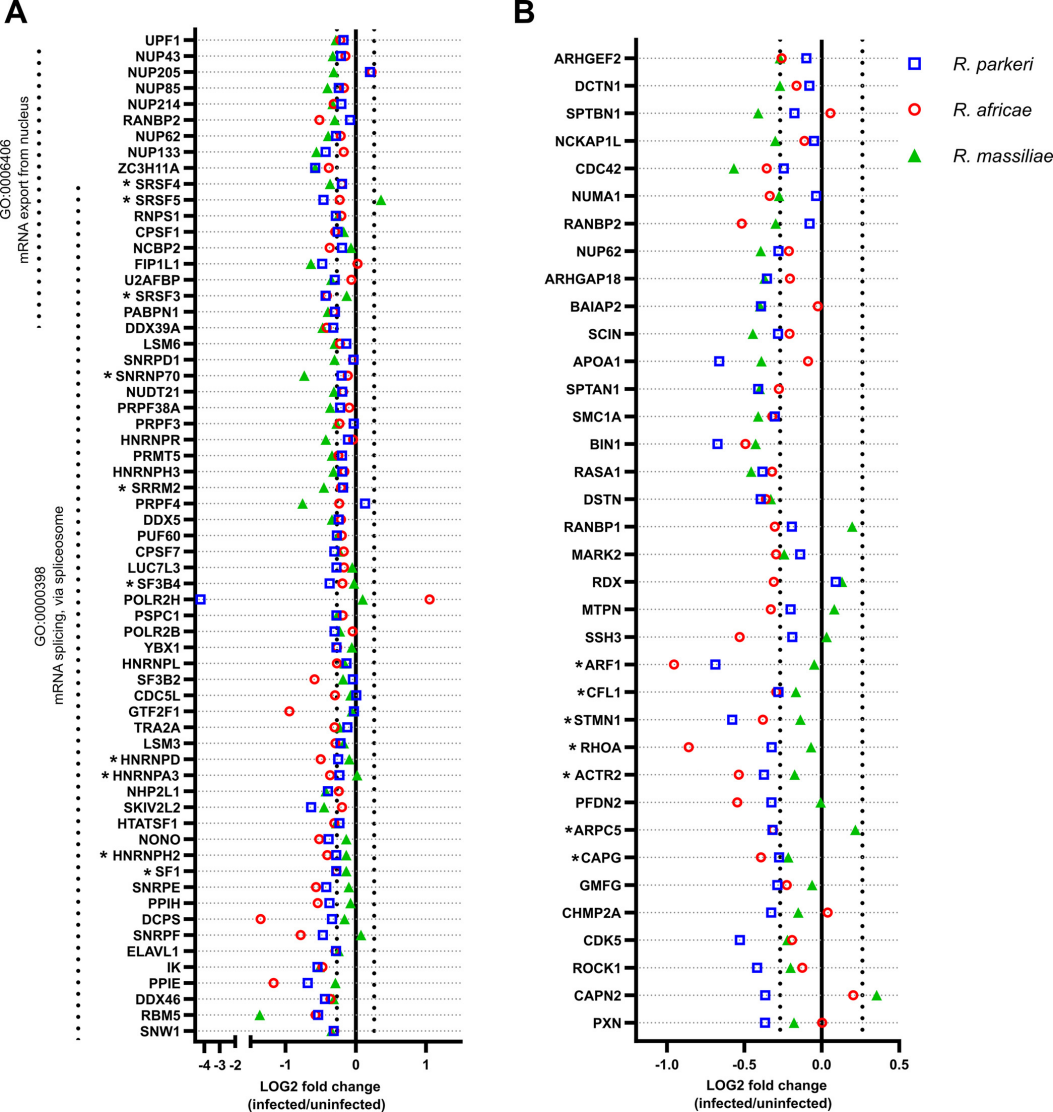
Rickettsial species responsible for mild rickettsioses affect several biological processes during infection of THP-1 macrophages. (A and B) Combined list of the individual gene names (and respective log_2_ fold change values) categorized with the GO terms mRNA splicing, via spliceosome (GO:0000398) and mRNA export from nucleus (GO:0006406) (A) and regulation of cytoskeleton organization (GO:0051493) (B) that display altered abundance in at least one infection condition. Colored circles represent individual proteins in THP-1 macrophages infected with R. parkeri (blue), R. africae (red), and R. massiliae (green). Gene names associated with an asterisk highlight proteins described in the text. Detailed information can be found in Table S2.

To provide insights into the molecular processes modulated by these rickettsial species, we started by analyzing the host proteins with altered abundance in each infection condition using the Search Tool for Retrieval of Interacting Genes/Proteins (STRING) (31). The global interaction networks obtained for host proteins with increased and decreased abundance upon *R. parkeri*, *R. africae*, and *R. massiliae* infection can be found in Fig. S2, S3, and S4, respectively, and the Gene Ontology (GO) enrichment plot integrating these results is illustrated in Figure 2. This analysis revealed the enrichment of several proteins categorized as neutrophil degranulation (GO:0043312), innate immune responses (GO:00045087), and vesicle-mediated transport (GO:0016192) in all three infection conditions. In contrast, clusters with GO associated with translation (GO:0006412) were observed only in R. parkeri- and R. africae-infected cells (Fig. 2; Fig. S2A, S3A, and S4A). Several clusters were also identified among proteins with reduced abundance (Fig. 2; Fig. S2B, S3B, and S4B). Interestingly, proteins associated with translation (GO:0006412) were also found to be underrepresented in *R. parkeri* and *R. africae*-infected cells, suggesting a common impact of infection with both species on this particular process. Additionally, other protein clusters found to have reduced abundance shared among the three infection conditions included GO terms categorized as mRNA splicing, via spliceosome (GO:0000398), mRNA export from nucleus (GO:0006406), neutrophil degranulation (GO:0043312) (also observed among overrepresented proteins), and regulation of cytoskeleton organization (GO:0051493). A cluster associated with DNA replication (GO:0006260) was observed in *R. parkeri*- and R. massiliae-infected cells, and one cluster associated with clathrin-mediated endocytosis (local STRING network cluster [CL] term identifier CL:19108) was found only in the former. This STRING analysis evidenced commonalities and specificities related to THP-1 cell responses to each of these mildly pathogenic rickettsiae. Therefore, to gain additional insights into the nature/identity of proteins altered in the most populated clusters and how these vary between infection conditions, we started by performing a detailed analysis of the proteins that showed altered abundance in at least one infection condition (Fig. 3 and 4; Table S2) (“innate immune responses” is explored in a different section below). Not unexpectedly, one of the obvious observations is that the abundance of many of these proteins varied differently among infection conditions, likely evidencing a different impact on these biological processes by each *Rickettsia* species. In the group of proteins categorized as neutrophil degranulation (GO:0043312) (Fig. 3A), we found several proteins associated with KEGG pathway identifiers for lysosomes and phagosomes, among which lysosomal protective protein/cathepsin A (GenBank accession no. CTSA [P10619]), cathepsin Z (CTSZ [accession no. Q9UBR2]), cathepsin D (CTSD [accession no. P07339]), with decreased abundance in the three data sets, and lysosome-associated membrane glycoprotein 2 (LAMP2 [accession no. P13473]) that was enriched in all infection conditions (Fig. 3A).

Regarding the cluster of proteins categorized as translation (GO:0006412) (Fig. 3B), we found that of the 57 host proteins identified in this GO group (which showed altered abundance in at least one infection condition), 40 and 38 proteins were considered altered in R. parkeri- and R. africae-infected cells, respectively, whereas only 18 proteins were significantly altered in R. massiliae-infected cells (Fig. 3B; Table S2), likely explaining why this cluster was not observed in *R. massiliae* (Fig. 2). Specifically, we found that several ribosomal proteins, such as 60S ribosomal protein L10 (RPL10 [accession no. P27635]), 60S ribosomal protein L36 (RPL36 [accession no. Q9Y3U8]), 40S ribosomal protein S15a (RPS15A [accession no. P62244]), 40S ribosomal protein S21 (RPS21 [accession no. P63220]), and 40S ribosomal protein S27 (RPS27 [accession no. P42677]), as well as eukaryotic translation initiation factor 3 subunit F (EIF3F [accession no. O00303]), showed reduced abundance in R. parkeri- and *R. africae*-infected cells only (Fig. 3B).

In the group of proteins that were categorized as mRNA splicing via spliceosome (GO:0000398) and mRNA export from nucleus (GO:0006406), there were a large number of splicing factors and accessory proteins, which play a critical role in regulating alternative splicing. Specifically, we found several serine-arginine-containing proteins (SR proteins), such as serine/arginine-rich splicing factor 3 (SRSF3 [accession no. P84103]), serine/arginine-rich splicing factor 4 (SRSF4 [accession no. Q08170]), serine/arginine-rich splicing factor 5 (SRSF5 [accession no. Q13243]), and serine/arginine repetitive matrix protein 2 (SRRM2 [accession no. Q9UQ35]), as well as several small nuclear ribonucleoproteins (snRNPs) and heterogeneous nuclear ribonucleoproteins (hnRNPs), such as U1 small nuclear ribonucleoprotein 70 kDa (SNRNP70 [accession no. P08621]), heterogenous nuclear ribonucleoprotein H2 (HNRNPH2 [accession no. P55795]), heterogenous nuclear ribonucleoprotein A3 (HNRNPA3 [accession no. P51991]), and heterogenous nuclear ribonucleoprotein D0 (HNRNPD [accession no. Q14103]), and splicing factors such as splicing factor 1 (SF1 [accession no. Q15637]), splicing factor 3B subunit 4 (SF3B4 [accession no. Q15427]) (Fig. 4A), with reduced abundance in at least one infection condition.

Analysis of the proteins that were categorized as regulation of cytoskeleton organization (GO:0051493) revealed that the macrophage-capping protein (CAPG [accession no. P40121]), actin-related protein 2/3 complex subunit 5 (ARPC5 [accession no. O15511]), actin-related protein 2 (ACTR2 [accession no. P61160]), transforming protein RhoA (RHOA [accession no. P61586]), stathmin (STMN1 [accession no. P16949]), cofilin-1 (CFL1 [accession no. P23528]), and ADP-ribosylation factor 1 (ARF1 [accession no. P84077]) showed reduced abundance in *R. parkeri*- and R. africae-infected cells only (Fig. 4B). Curiously, capping protein and cofilin were shown to play an essential role in *R. parkeri*’s motility (32). The changes in protein abundance observed herein suggest different impacts on host cytoskeleton components/organization between these three SFG *Rickettsia* species and raise intriguing questions about the repercussions on actin-based motility mechanisms.

### SFG *Rickettsia* species induce substantial alterations in the proteome of THP-1 macrophages in a species-dependent manner.

**FIG 5:**
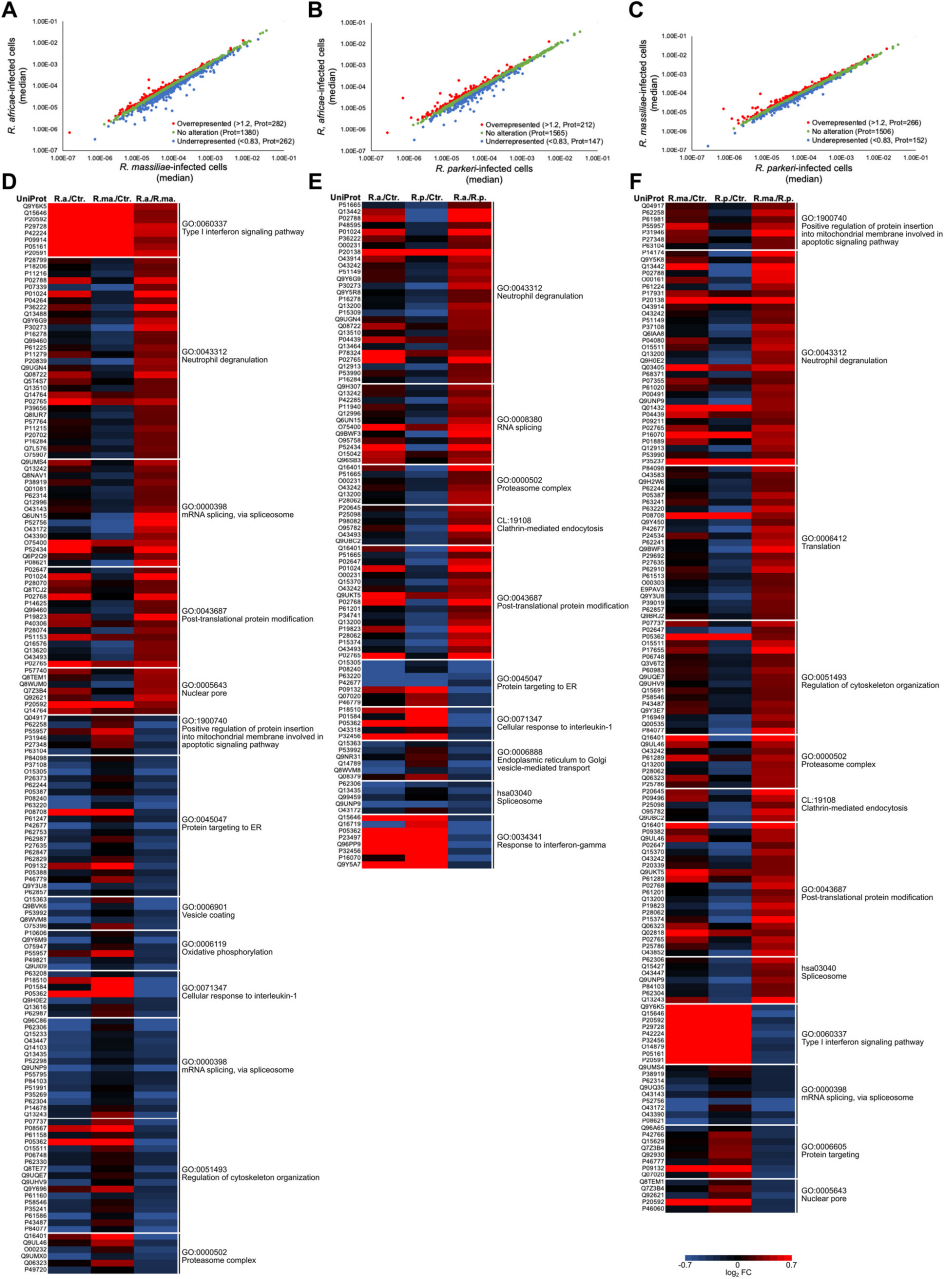
SFG *Rickettsia* species induce substantial alterations in the proteome of THP-1 macrophages in a species-specific manner. (A to C) Scatterplot representation comparing global changes in protein abundance between two infection conditions. Comparison between R. africae-infected cells and *R. massiliae*-infected cells (A), *R. africae*-infected cells and R. parkeri-infected cells (B), and R. massiliae-infected cells and *R. parkeri*-infected cells (C). The 1,925 proteins that were confidently quantified in all 4 experimental conditions were plotted and considered altered when a change of at least 20% in abundance (fold change of ≤0.83 or ≥1.2) was observed between infection conditions. Proteins that were considered to decrease, not change, or increase their abundance upon infection are represented in blue, green, and red, respectively. See also Table S3. (D to F) Host proteins that changed abundance between two infection conditions were analyzed in STRING to identify protein network clusters and their respective classification according to gene ontology (GO) or local STRING network cluster (CL) terms. The heatmaps display alterations in the abundance of individual host proteins and their respective GO or CL term classification. The heatmaps compare alterations between *R. africae*-infected cells and *R. massiliae*-infected cells (D), *R. africae*-infected cells and *R. parkeri*-infected cells (E), and *R. massiliae*-infected cells and *R. parkeri*-infected cells (F). The blue (decreased) and red (increased) color scale indicates alterations in protein abundance (log_2_ ratio) between infection conditions. See also Table S4.

The difference in the nature of protein network clusters observed for each infection condition and the abundance of individual host proteins within each GO group suggested alterations of the macrophage proteome that are species specific. To further evaluate this, we analyzed the relative protein quantifications between infection conditions of R. africae/R. massiliae, *R. africae*/R. parkeri, and *R. massiliae*/*R. parkeri*, following the same criteria as previously. The results are illustrated in Figure 5A to C. We could observe substantial alterations (of at least 20%) in the abundance of 534 (corresponding to 27.7% of the quantified proteins) host proteins between THP-1 macrophages infected with *R. africae* and *R. massiliae* (Fig. 5A; Table S3), 359 (18.6% of the quantified proteins) between *R. africae*- and *R. parkeri*-infected cells (Fig. 5B; Table S3), and 418 (21.7% of the quantified proteins) between R. massiliae- and *R. parkeri*-infected cells (Fig. 5C; Table S3). To shed light on the cellular processes represented by these proteins, we performed a STRING analysis and evaluated the most representative GO terms among each of these groups. The heat maps shown in Figure 5D to F represent the changes in protein abundance between infection conditions within these clusters, confirming that the pattern of host protein alterations differed between rickettsial species. For example, although both R. africae and *R. massiliae* induced the accumulation of several proteins that were categorized as type I interferon signaling (GO:0060337), the levels of accumulation of several of these proteins, including the interferon-induced GTP-binding protein Mx1 (accession no. P20591) and 2′-5′-oligoadenylate synthase 2 (accession no. P29728), were significantly higher in *R. africae*-infected cells, possibly suggesting higher type I IFN responses in *R. africae*-infected cells at this time point postinfection (Fig. 5D; Table S4). Indeed, we provide further validation of this trend later in this work. Proteins associated with neutrophil degranulation (GO:0043312), mRNA splicing, via spliceosome (GO:0000398), and the nuclear pore (GO:0005643) also displayed significant differences in accumulation between *R. africae*-infected cells and *R. massiliae*-infected cells. Moreover, several proteins that were categorized as cellular response to interleukin 1 (GO:0071347), including the interleukin 1 receptor antagonist protein (accession no. P18510), intercellular adhesion molecule 1 (accession no. P05362), and interleukin 1β (accession no. P01584), were found in lower abundance in R. africae-infected cells than in R. massiliae-infected cells (Fig. 5D; Table S4), IL-1 β validated later in this work.

Comparing *R. africae*-infected cells with *R. parkeri*-infected cells, we also observed that several proteins that were categorized as proteasome complex (GO:0000502), as well as several proteins categorized as clathrin-mediated endocytosis (CL:19108), were found in lower abundance in *R. parkeri*-infected cells than in *R. africae*-infected cells (Fig. 5E; Table S4). Interestingly, proteins that were categorized as cellular response to interleukin 1 (GO:0071347), including the interleukin 1 receptor antagonist protein (accession no. P18510), interleukin 1β (accession no. P01584), intercellular adhesion molecule 1 (accession no. P05362), and guanylate-binding protein 2 (accession no. P32456), as well as other proteins that were categorized as response to interferon gamma (GO:0034341), including kynureninase (accession no. P23497), nuclear autoantigen Sp-100 (accession no. P23497), guanylate-binding protein 4 (accession no. Q96PP9), CD44 antigen (accession no. P16070), and NEDD8 ultimate buster 1 (accession no. Q9Y5A7), were found in higher abundance in *R. parkeri*-infected cells than in R. africae-infected cells (Fig. 5E; Table S4), evidencing differences in host inflammatory responses.

When comparing *R. massiliae-* and *R. parkeri*-infected cells, the pattern of host protein alterations was also different for several cellular processes (Fig. 5F; Table S4). As an example, several proteins that were categorized as regulation of cytoskeleton organization (GO:0051493) and others that were categorized as clathrin-mediated endocytosis (CL:19108) were found in lower abundance in *R. parkeri*-infected cells than in R. massiliae-infected THP-1 macrophages (Fig. 5F; Table S4). Proteins categorized as type I interferon signaling (GO:0060337) were found in lower abundance in *R. massiliae*-infected cells than in THP-1 macrophages infected with *R. parkeri* (Fig. 5F; Table S4), again evidencing differences that we validate later in this study.

Although all the SFG *Rickettsia* species used herein possess the ability to subvert macrophage-mediated killing mechanisms and establish a replicative niche within THP-1 macrophages, they trigger substantially different proteomic signatures in a species-specific manner.

### SFG *Rickettsia* species responsible for mild rickettsioses elicit changes in the abundance of several proteins of the RIG-I-like receptor signaling pathway.

**FIG 6:**
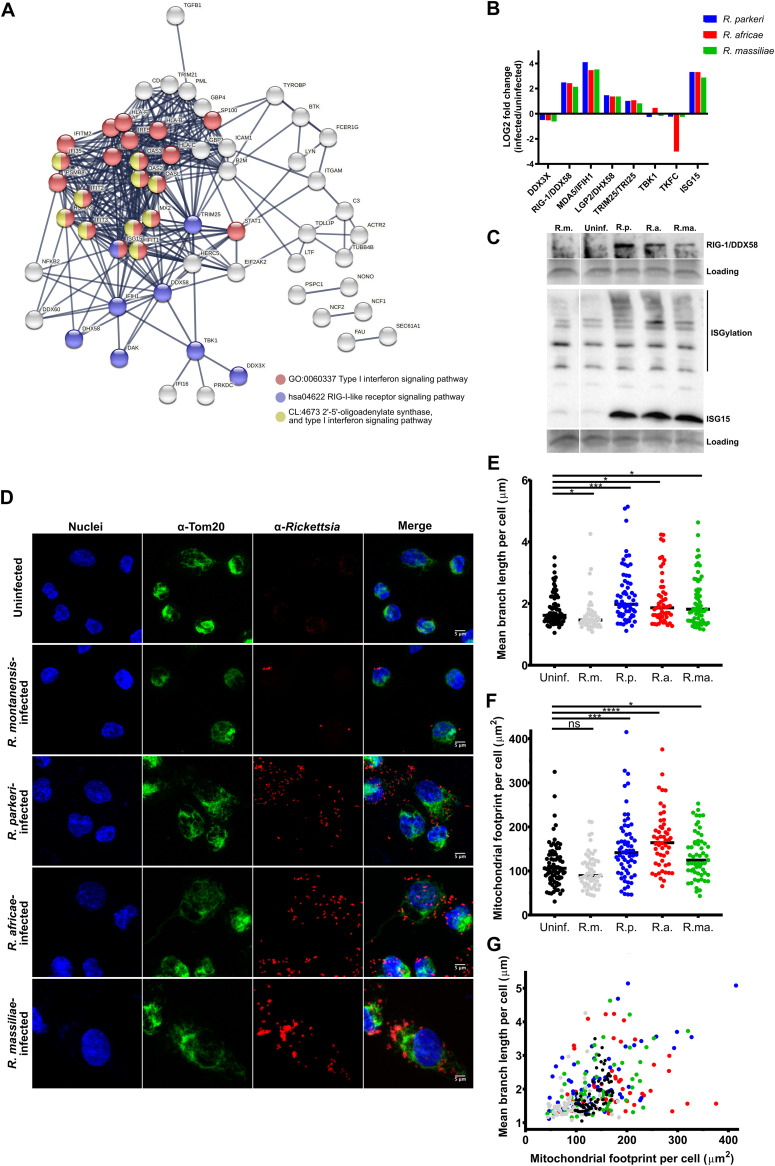
SFG *Rickettsia* species modulate multiple components impacting the RIG-I-like receptor signaling pathway. (A) STRING network analysis of the 80 proteins categorized as innate immune response (GO:00045087) that show altered abundance in at least one infection condition. The analysis was carried out using the highest confidence score (0.9) and MCL clustering with an inflation parameter of 3. Nodes are represented with different colors according to their categorization in gene ontology (GO) terms. See also Table S5. (B) Individual analysis of the host proteins (and respective log_2_ fold change values as determined by SWATH-MS/MS) identified in KEGG pathways as RIG-I-like receptor signaling pathway (hsa04622) that show altered abundance in at least one infection condition. Color bars represent individual proteins in THP-1 macrophages infected with R. parkeri (blue), R. africae (red), and R. massiliae (green). See also Table S5. (C) Western blot analysis of total protein extracts (15 μg) from uninfected THP-1 macrophages (uninf.) and THP-1 macrophages infected with R. montanensis (R.m.), *R. parkeri* (R.p.), *R. africae* (R.a.), and *R. massiliae* (R.ma.). Protein samples were probed for retinoic acid-inducible gene I (RIG-1 [accession no. O95786]) and ubiquitin-like protein ISG15 (accession no. P05161), and immunoblot analysis with SERVA purple was used as the protein loading control. (D) Evaluation of mitochondrial morphology by immunofluorescence microscopy of uninfected and R. montanensis*-*, *R. parkeri-*, *R. africae-*, and *R. massiliae-*infected THP-1 macrophages (MOI of 10) at 24 h postinfection. Cells were stained with DAPI (blue) to identify host nuclei, anti-Tom20 antibody (F-10) (sc-17764) followed by Alexa Fluor 647 goat anti-mouse antibody (green) to identify mitochondria, and anti-*Rickettsia* antibody (anti-RcPFA antibody) (NIH/RML I7198) followed by Alexa Fluor 568 (red) to identify *Rickettsia*. Scale bar = 5 μm. (E to G) Quantification of alterations in mitochondrial network morphology parameters using the morphometric ImageJ plugin tool MiNA on mitochondrion-labeled images. Scatterplots show the quantification of mitochondrial footprints (mitochondrial mass in a cell) (E), the mean branch lengths (average size of all branches) (F), and 2-dimensional representations of mitochondrial footprint versus mean branch length (G) for uninfected (black) and R. montanensis*-* (gray), *R. parkeri-* (blue), *R. africae-* (red), and *R. massiliae* (green)-infected THP-1 macrophages. Dots are data from individual cells (*n* > 50), and mean values (horizontal bar) ± standard deviations (SD) are representative of results from three independent experiments. Significance was determined by Brown-Forsythe and Welch analysis of variance (ANOVA) using GraphPad Prism 8 (ns, not significant; *, *P* < 0.1; ***, *P* < 0.001; ****, *P* < 0.0001). Statistical analysis was performed using GraphPad Prism version 8.0.1 (GraphPad Software, Inc., CA, USA).

As shown by the results in Figure 2 and Fig. S2 to S4, our data revealed that infection of THP-1 macrophages with SFG *Rickettsia* species responsible for mild rickettsioses induces the accumulation of several proteins categorized as innate immune responses (GO:00045087). We analyzed the 80 proteins categorized in this cluster that had altered abundance in at least one infection condition (Table S5). The observed enrichment pattern revealed as top categories the type I interferon signaling pathway (GO:0060337), RIG-I-like receptor signaling pathway (hsa04622), and 2′-5′-oligoadenylate synthase and type I interferon signaling pathway (CL:4673) (Fig. 6A). The RLR signaling pathway has been identified as an important component of the innate immune system that detects intracellular infections and generates actions to limit pathogen replication (33, 34). However, to our knowledge, this pathway has not been previously identified in *Rickettsia*-macrophage interactions (23, 24). Interestingly, our results revealed that the three proteins identified as cytoplasmic RNA sensors, RIG-I/DDX58 (accession no. O95786), melanoma differentiation associated factor 5 (MDA5/IFIH1 [accession no. Q9BYX4]), and probable ATP-dependent RNA helicase DHX58 (LGP2/DHX58 [accession no. Q96C10]), as well as the E3 ubiquitin/IFN-stimulated gene 15 (ISG15) ligase TRIM25 (TRIM25/TRI25 [accession no. Q14258]), which mediates the ubiquitination of RIG-I and MDA5, and ISG15 (accession no. P05161), which has been described as a product and a regulator of the activation of this pathway, are among the proteins found with the highest abundance upon infection (Fig. 6B; Table S5). Notably, the dihydroxyacetone kinase protein (dihydroxyacetone kinase [accession no. Q3LXA3]), described as a negative regulator of MDA5 (35), is found with decreased abundance in *R. africae*-infected THP-1 macrophages only, pointing toward species-specific alterations.

As a follow-up validation, we performed Western blot analysis of uninfected and infected THP-1 macrophages against RIG-1 and ISG15 (Fig. 6C). These results showed the same trend in alterations in protein abundance between the MS-based proteomics data and the Western blot results. Moreover, an increased smear pattern of ISG15 conjugated to target proteins (ISGylation), which is known to be strongly induced by type I interferon responses, was visible at this time point postinfection in cells infected with pathogenic *Rickettsia* only. None of these changes in protein abundance were observed in uninfected or R. montanensis-infected cells (used here as a nonpathogen control that cannot proliferate in macrophages) (19, 22, 23). These results are consistent with the augmented accumulation of interferon-stimulated genes observed in the quantitative SWATH-MS data for the three mildly pathogenic rickettsiae.

It has been shown that RLR activation promotes elongation of the mitochondrial network, thus facilitating mitochondrial antiviral-signaling protein (MAVS) interactions with downstream signaling molecules (36, 37). So, we next sought to determine if infection of THP-1 macrophages with *R. parkeri*, *R. africae*, and *R. massiliae* would promote alterations in mitochondrial morphology compared with the results of infection with the nonpathogen R. montanensis. To investigate this, THP-1 macrophages were infected with the different rickettsial species (MOI of 10) or left uninfected as a control, and at 24 hpi, cells were fixed and mitochondria immunolabeled with anti-TOM20 antibody for confocal microscopy analysis. Indeed, we observed an expansion of the mitochondrial network in THP-1 macrophages infected with R. parkeri, R. africae, and R. massiliae compared with uninfected or R. montanensis-infected cells (Fig. 6D). To obtain an unbiased and quantitative representation of these observations, we used the semiautomated morphometric tool MiNA to analyze the mitochondrial network morphology (38). Our results showed that infection with the pathogenic species induced a significant increase in the mean branch length (reflecting the average size of all branches) compared with the branch length in uninfected cells. In contrast, a significant reduction in these values was observed in R. montanensis (nonpathogen)-infected cells (Fig. 6E). Moreover, mitochondria of *R. parkeri*-, *R. africae*- and *R. massiliae*-hijacked macrophages also showed a significant increase in the mitochondrial footprint, reflecting mitochondrial mass in a cell, compared with that in uninfected and R. montanensis-infected cells (Fig. 6F). These higher values in footprint and mean branch length (Fig. 6G) are consistent with an expansion/fusion of the mitochondrial network footprint in THP-1 macrophages infected with these mildly pathogenic rickettsiae, thus reinforcing the RLR pathway as one of the routes for type I interferon production.

### *Rickettsia*-dependent regulation of interleukin 1 and type I interferon responses during infection.

**FIG 7:**
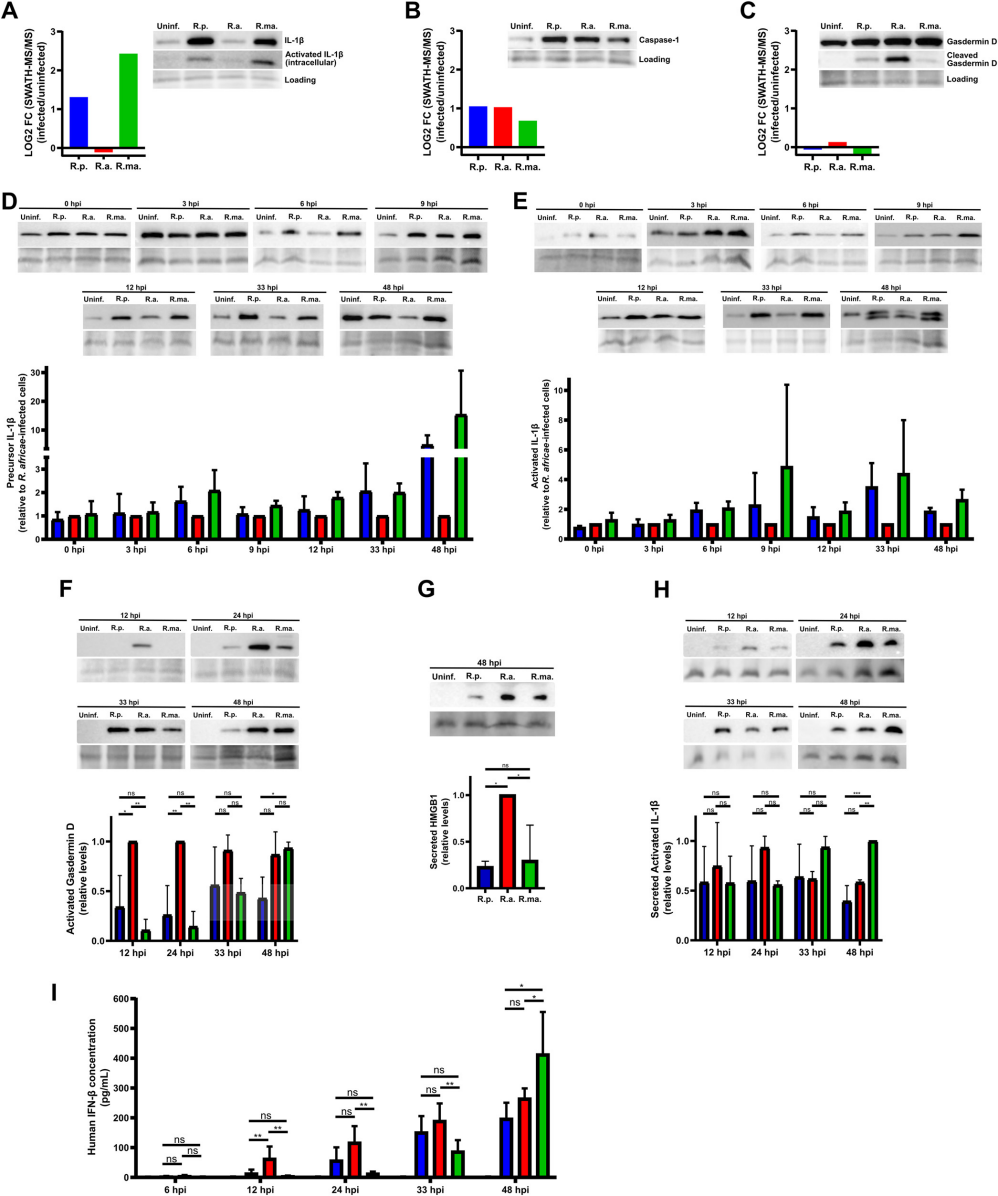
*Rickettsia*-dependent regulation of IL-1β and IFN-β responses during infection. (A to C) Bar chart of log_2_ fold change for the respective individual protein as determined by SWATH-MS between R. parkeri- (blue), R. africae- (red), and R. massiliae- (green)-infected and uninfected THP-1 macrophages, and the corresponding Western blot analyses. Total protein extracts (15 μg) from uninfected THP-1 macrophages (uninf.) and *R. parkeri*- (R.p.), *R. africae*- (R.a), and *R. massiliae*- (R.ma.) infected THP-1 macrophages were probed for interleukin 1β (precursor and activated forms) (IL-1β [accession no. P01584]) (A), caspase-1 (accession no. P29466) (B), and gasdermin D (precursor and activated forms) (accession no. P57764) (C). Immunoblot analysis with SERVA purple was used as the protein loading control. (D to H) Western blot analysis, loading controls, and respective quantification for the corresponding individual protein on uninfected (uninf.) and *R. parkeri*-, *R. africae*-, and *R. massiliae*-infected THP-1 macrophages at the indicated time points postinfection. Bars represent mean values ± SD from three independent experiments. (D and E) Evaluation of the intracellular levels of precursor (D) and activated (E) forms of IL-1β in total protein extracts (15 μg). Immunoblot analysis with SERVA purple was used as the protein loading control, and results were normalized for *R. africae*-infected cells. (F) Evaluation of the intracellular levels of activated gasdermin D in total protein extracts (25 μg). Immunoblot analysis with SERVA purple was used as the protein loading control and results were normalized for the highest-intensity band. (G) Evaluation of the secreted levels of HMGB1 in culture supernatants (50 μl). Results were normalized for the highest-intensity band. Loading controls are shown below each group. (H) Evaluation of the secreted levels of activated IL-1β in culture supernatants (50 μl). Results were normalized for the highest-intensity band. Loading controls are shown below each group. (I) Determination of the levels of IFN-β in culture supernatants of uninfected (white) and *R. parkeri*-, *R. africae*-, and *R. massiliae*-infected THP-1 macrophages by ELISA at the respective time points postinfection. Statistical analysis was performed using ordinary one-way ANOVA with correction for multiple comparisons using statistical hypothesis testing (Tukey). Significant differences are indicated as follows: ns, nonsignificant; *, *P* < 0.05; **, *P* < 0.01; ***, *P* < 0.001. Statistical analysis was performed using GraphPad Prism version 8.0.1 (GraphPad Software, Inc., CA, USA). Raw data of the cropped Western blot images and loading controls can be found in the supplemental material (Fig. S5 to S20).

Innate cytokines are central players of the inflammatory response, in which type I interferon and interleukin 1 represent the pillars of distinct and specialized pathways (39). We have found that several proteins categorized as cellular response to interleukin 1 (GO:0071347) (Fig. 5D to F), including IL-1β (accession no. P01584) itself, showed decreased abundance in THP-1 macrophages infected with *R. africae* compared to their levels in *R. parkeri*- and *R. massiliae*-infected cells (Fig. 7A). To further confirm these results, we evaluated the intracellular levels of IL-1β by Western blotting at 24 hpi. Again, our results confirmed the lower intracellular bioavailability not only of the precursor form of IL-1β but also of its activated product in *R. africae*-infected cells (Fig. 7A). Notably, our proteomics data and follow-up validation by Western blotting demonstrated that the markers of inflammasome and pyroptosis, caspase-1 and cleaved gasdermin D, followed a different trend (Fig. 7B and C). The intracellular levels of the cysteine protease caspase-1 (accession no. P29466), which activates itself and converts the immature pro-IL-1β into its mature form after recruitment by the inflammasome complex (40), were found to be enriched in THP-1 macrophages upon infection with all rickettsial species (Fig. 7B). The pyroptosis inducer gasdermin D (GSDMD [accession no. P57764]) is cleaved by activated caspase-1 to release the membrane pore-forming GSDMD-N domain, thus contributing to the release of activated IL-1β, IL-18, and DAMPS (40, 41). Our Western blot analysis revealed that, at 24 hpi, the intracellular levels of activated gasdermin D were higher in R. africae-infected THP-1 macrophages than in *R. parkeri*- and R. massiliae-infected cells (Fig. 7C), evidencing a different impact on the inflammasome/IL-1β axis upon *R. africae* infection.

Taking this into account, we next sought to determine the intracellular levels of IL-1β (both precursor and mature forms) at different time points postinfection by Western blotting. Our results confirmed lower bioavailability of both (precursor and mature) forms of this proinflammatory cytokine in *R. africae*-infected cells across several time points postinfection (as early as 6 hpi) (Fig. 7D and E). However, consistent with our previous result, we observed significantly higher levels of activated gasdermin D in *R. africae*-infected THP-1 macrophages than in the other infection conditions at 12 and 24 hpi, suggesting that pyroptotic events are triggered earlier by *R. africae* (Fig. 7F). We then evaluated the levels of high mobility group protein B1 (HMGB1) in the supernatant of *Rickettsia-*infected THP-1 macrophages to confirm this observation. Our results confirmed significantly higher levels of HMGB1 in the culture medium of R. africae-infected cells, thus corroborating more pyroptotic events in this infection condition (Fig. 7G). However, when assessing the levels of secreted activated IL-1β at different time points postinfection, our results revealed no significant differences between infection conditions, except at 48 hpi for *R. massiliae*-infected cells (Fig. 7H). Therefore, although pyroptotic events appear to be triggered earlier in *R. africae*-infected cells, this does not contribute to increased secretion of IL-1β, pointing again to differential regulation of the production of this proinflammatory cytokine in *R. africae*-infected cells.

Several reports have demonstrated the cross talk between IL-1 and type I IFN in regulating inflammatory responses (39, 42, 43). It has been demonstrated that type I IFN can inhibit IL-1 production by either repressing inflammasome activity or reducing the abundance of pro-IL-1β in a STAT1/IL-10-dependent manner (43). By acting as a possible counterregulator of pro-IL-1β production, we hypothesized that these rickettsial species would trigger differential levels of type I IFNs in THP-1 macrophages, as evidenced by the observed differences in the abundance of several ISGs between infection conditions (Fig. 5D and F). To further evaluate this, we determined the concentrations of secreted IFN-β at different time points postinfection (Fig. 7I). Our results confirmed significantly higher levels of this cytokine in R. africae-infected cells than in *R. parkeri* and *R. massiliae* infections at 12 hpi, with these levels remaining significantly higher than in *R. massiliae*-infected cells for 24 and 33 hpi. In fact, IFN-β levels in *R. massiliae* infection only increased at later times postinfection. This evidence confirmed that infection by different rickettsial species induced different levels of type I IFN. Combined with the differences in IL-1β bioavailability, these results indicate that although R. parkeri, *R. africae*, and R. massiliae all replicate successfully within THP-1 macrophages, they trigger qualitatively and quantitatively distinct inflammatory responses during infection.

### THP-1 cell signatures in response to infection with mildly pathogenic SFG *Rickettsia* species versus the highly pathogenic R. conorii.

The species-specific differences in host responses observed between mildly pathogenic *Rickettsia* species led us to compare those responses with our previously published data sets for the highly pathogenic R. conorii and the nonpathogen R. montanensis (23). To evaluate this, we crossed the data sets and identified a total of 684 host proteins that were confidently quantified in all infection conditions and that could be used for comparison purposes (Table S6). Importantly, we found that the proteins related to interferon-stimulated responses were not present in this merged data set. Indeed, in THP-1 cells infected with the highly pathogenic R. conorii, this drastic increase in interferon-stimulated responses was not quantifiable/observable (23). Considering that, we remined the SWATH files acquired at that time for R. conorii-infected and uninfected cells and extracted the information with the library of identified proteins in the present data set for four proteins of interest (IL-1β, Mx1 [accession no. P20591], ISG15, and RIG-I). As detailed in the supplemental material (Fig. S21 to S26), from the analyzed proteins, only IL-1β was undoubtedly present in R. conorii-infected THP-1 cells with high intensity. For the three interferon-induced proteins, the selected peptides were not found in the R. conorii data set (or any control sample) for Mx1 and RIG-I, whereas for ISG15, there was a visible peak with very low intensity, indicating that the protein may be present but is not regulated in this infection condition. Taken together, these results indicate marked differences in interferon-stimulated responses triggered by mildly pathogenic *Rickettsia* species versus the highly pathogenic R. conorii.

**FIG 8:**
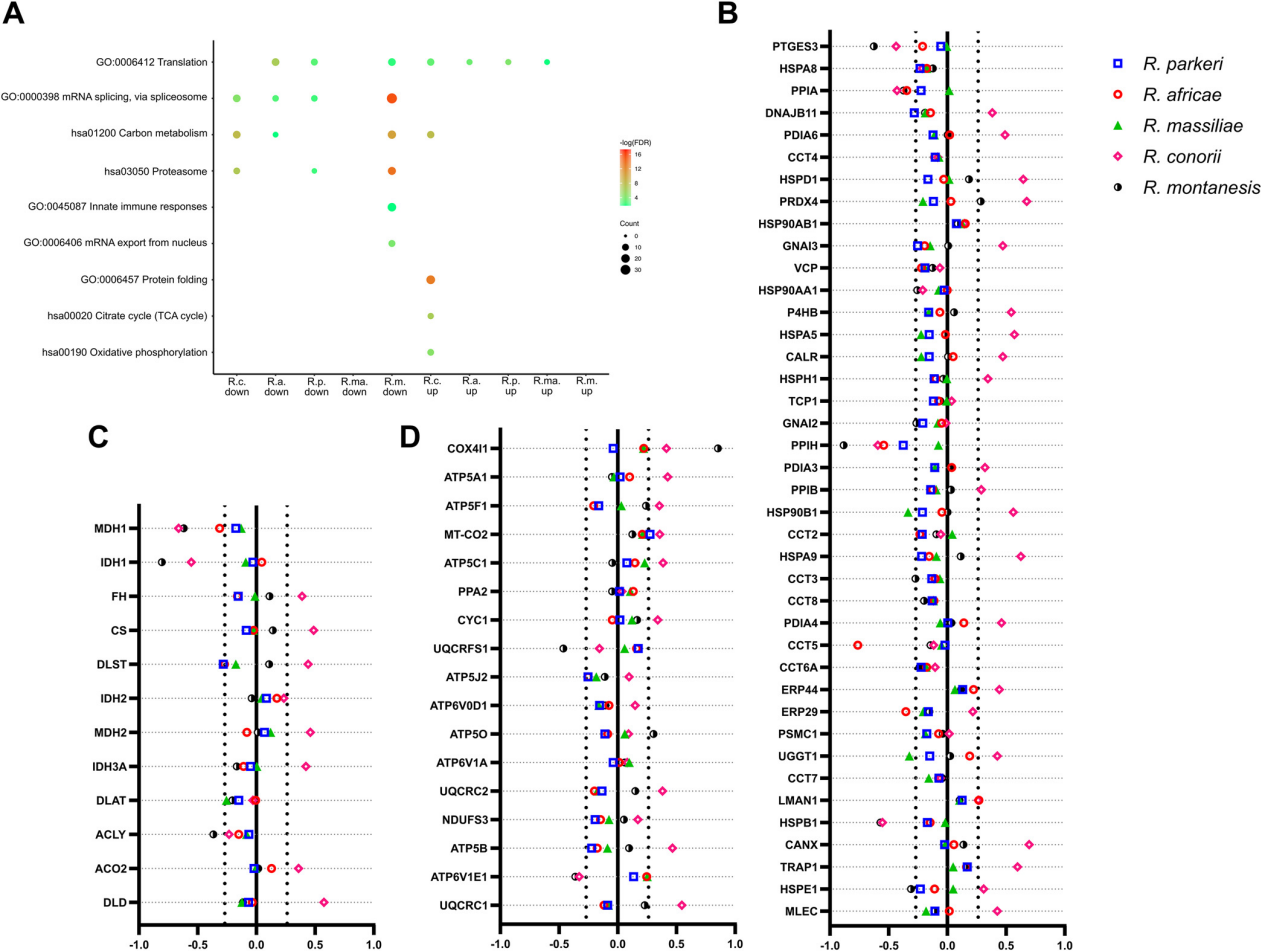
THP-1 signatures differ in response to infection with mildly pathogenic SFG *Rickettsia* species versus the highly pathogenic R. conorii. (A) Gene Ontology analysis of 684 host proteins with altered abundance upon infection that were confidently quantified in all of the following infection conditions: R. conorii- (R.c.), R. africae- (R.a.), R. parkeri- (R.p.), R. massiliae*-* (R.ma.), and R. montanensis- (R.m.) infected THP-1 macrophages. GO Enrichment Plot function in Image GP (http://www.ehbio.com/ImageGP/) was used to integrate and represent the Gene Ontology analysis. Analyses of the host proteins with decreased and increased abundance upon infection with each bacterial species are represented by “down” and “up,” respectively. The dot size indicates the number of host proteins with altered abundance for the specific infection condition of the selected GO or KEGG pathway (hsa) categorization. The dot color indicates the significance of the enrichment [−log_10_(FDR-corrected *P* values)]. (B to D) Combined list of the individual gene names (and respective log_2_ fold change values) categorized with the GO or hsa term protein folding (GO:0006457) (B), TCA cycle (hsa00020) (C), and oxidative phosphorylation (hsa00190) (D). Colored circles represent individual proteins in THP-1 macrophages infected with R. conorii (pink), *R. parkeri* (blue), *R. africae* (red), and *R. massiliae* (green), and R. montanensis (black and white). Detailed information can be found in Table S6.

To gain more insights into other cellular signatures that may distinguish highly and mildly pathogenic *Rickettsia* species, we performed a GO analysis of this merged data set. The GO enrichment plot integrating these results is shown in Figure 8A. Our results point toward substantial differences in the levels of several proteins categorized as protein folding (GO:0006457), tricarboxylic acid (TCA) cycle (hsa00020), and oxidative phosphorylation (hsa00190), which mainly showed increased abundance in R. conorii-infected THP-1 macrophages only (details of proteins and log_2_ fold change values are listed in Fig. 8B to D and Table S6). These observations suggest potential differences also in host metabolic rewiring and protein folding capacity between R. conorii and the other, mildly pathogenic species.

## DISCUSSION

This work provides evidence that R. parkeri, R. africae, and R. massiliae, all responsible for mild rickettsioses, trigger different proteome signatures in THP-1 macrophages upon infection, indicating species-dependent differential impacts in several host cellular processes. Among the differences observed, our results confirm distinct impacts on inflammatory responses between species. Although all species triggered type I interferon responses, the differences in the levels of IFN-β and bioavailability of IL-1β between infection conditions further strengthen the evidence of variations among rickettsiae in their ability to manipulate the host. Moreover, our quantitative proteomics analysis reveals significant impacts of infection with these mildly pathogenic rickettsiae on various macrophage cellular processes, highlighting the exciting complexity of rickettsia-host interactions.

Proteolytic enzymes like cathepsins participate in the intracellular digestion of bacteria. This event contributes directly to the elimination of the invading pathogen and indirectly to the generation of bacterial antigens for presentation, thus supporting adaptive immune responses (44). We found that *Rickettsia*-infected THP-1 macrophages displayed reductions in the amounts of cathepsin A/lysosomal protective protein (CtsA), cathepsin D (CtsD), and cathepsin Z (CtsZ) at 24 hpi. These results are in agreement with our previous observations of lower accumulation of CtsD and CtsZ in macrophages infected with the highly pathogenic R. conorii (23). Many bacterial pathogens have evolved multiple strategies for controlling the expression and proteolytic activity of cathepsins to limit their antibacterial action and subsequently favor intracellular pathogen survival (44–48). Therefore, understanding the cathepsin modulation strategies of *Rickettsia* and how the dysregulation of these proteases contributes to the pathogenesis of rickettsial infections through their role in both innate and adaptive immune functions of macrophages warrants further investigation.

Alterations in host RNA splicing patterns upon infection have been reported as a means to tweak the host immune responses against the pathogen, thus emerging as a new paradigm in host-pathogen interactions (49–52). The implications of alterations in alternative splicing during infection are remarkable, since they can influence a wide variety of aspects, such as transcript stability, stability of the translated products, loss/gain of function, interacting partners, and subcellular localization (49, 50). Although this is more extensively reported during viral infections, (a few) recent studies also demonstrate bacterial interference with the host splicing machinery (49). One example is the massive alterations in the RNA splicing pattern of human macrophages upon infection with Mycobacterium tuberculosis, with alternative spliced variants of host genes resulting in better survival of M. tuberculosis strains within these cells (53). Serine-arginine-containing proteins (SR proteins) and ribonucleoproteins (RNPs) are critical regulators of alternative splicing, and therefore, alterations in the activity, localization, and concentration of these regulatory proteins can impact the cellular alternative splicing landscape (49). We found that infection of THP-1 macrophages with rickettsial species decreased the accumulation of a large number of splicing factors and other accessory proteins, such as small nuclear ribonucleoproteins (snRNPs) and heterogenous nuclear ribonucleoproteins (hnRNPs), suggesting pivotal alterations in the host RNA splicing pattern during rickettsial infections.

Another step of host gene expression known to be impacted by microbial infection is protein synthesis (54, 55), with a growing number of reports demonstrating the ability of pathogens to interfere with host mRNA translation to trigger and/or limit the production of host immune defenses (54, 55). Our results showed that infection of THP-1 macrophages with *R. africae* and *R. parkeri* (less so for *R. massiliae*) altered the abundance of a significant number of proteins involved in translation, including several ribosomal proteins and eukaryotic initiation factors (eIFs). Specifically, we found significant reductions in the abundance of the translation initiation factor eIF-2B subunit delta in all *Rickettsia*-infected cells and the eukaryotic translation initiation factor 3 subunit F in *R. parkeri*- and *R. africae*-infected macrophages. Our data sets also revealed substantial alterations in the abundance of several subunits of the 40S and 60S ribosomal proteins in *Rickettsia*-infected cells, possibly resulting in modifications in the ribosome composition and subsequent alterations in the host translation machinery. Distinct impacts of bacterial infections on translation have been found between the action of specific toxins that directly inhibit translation (e.g., in Pseudomonas aeruginosa and Legionella pneumophila infections) and by induction of stress pathways that result in general host metabolic stress responses to infection (e.g., in Chlamydia, Shigella flexneri, and Salmonella species infections) (56, 57). Curiously, recent findings with the *Rickettsiales* member *Wolbachia* provided evidence for a *Wolbachia*-host translation interaction by revealing that perturbations in ribosome components and eIFs by RNA interference (RNAi) or mutations result in increased bacterial levels, both *in vitro* and *in vivo* (58). Our work provides evidence that *Rickettsia* species may also interfere with the ongoing host protein synthesis during infection. However, whether this potential dampening of protein translation results from host stress responses to infection or an active manipulation of the host translation machinery by *Rickettsia* remains an exciting open question. Curiously, it has been demonstrated that viruses manipulate host translation through proteolytic cleavage of eIFs (55). One specific example is the HIV-1 protease, which has been shown to be capable of cleaving the eukaryotic initiation factor 4G (eIF4G) and subunit d of eukaryotic initiation factor 3 (eIF3d) to facilitate HIV disease progression (59–63). We have previously identified a membrane-embedded retropepsin-like enzyme (APRc) that is highly conserved across *Rickettsia* genomes and shares structural and functional features with the HIV-1 protease (64, 65). Evaluating eukaryotic initiation factors as potential substrates for APRc during rickettsial infections and the subsequent impacts on rickettsial pathogenesis emerges as an exciting hypothesis and a new avenue of research in our laboratory. Perturbation of host translation and RNA splicing machineries during infection with mildly pathogenic rickettsial species and analysis of the consequent impacts of these interactions on the host immune responses and pathogenesis of the disease reveal additional layers of complexity to the *Rickettsia*-host interactions that call for further research.

Innate immune responses are the first line of defense against invading pathogens (39, 42). Infection of THP-1 macrophages with R. parkeri, R. africae, and R. massiliae caused the accumulation of many proteins associated with innate immunity, with the products of several ISGs being among the proteins with the highest increases in abundance upon infection. Among these proteins are guanylate-binding protein 1 (GBP1), guanylate-binding protein 2 (GBP2), and guanylate-binding protein 4 (GBP4), which are interferon-inducible GTPases of the dynamin GTPase superfamily that have been shown to restrict the replication of intracellular pathogens in both immune and nonimmune cells (66). Another group of ISGs that we found to have increased abundance in *Rickettsia*-infected THP-1 macrophages were the 2′-5′-oligoadenylate synthetase (OAS) molecular innate immune sensors, including OAS2, OAS3, and OASL, which can detect cytosolic double-stranded RNA (dsRNA) in order to activate the latent RNase L (RNase L) to induce degradation of pathogen and cellular RNAs and thereby block pathogen replication (67). Also, we found that several protein members of the RLR pathway, including the cytoplasmic RNA sensors RIG-I, MDA5, and LGP2, as well as TRIM25 and ISG15, were among the proteins with increased abundance in *Rickettsia*-infected THP-1 macrophages. Remarkably, the protein DAK, described as a physiological repressor of MDA5-mediated signaling (35), showed lower abundance in *R. africae*-infected cells only. However, the contribution of this species-specific decrease in DAK abundance for a more pronounced activation of the RLR pathway in *R. africae*-infected cells warrants future investigation. Overall, these results are consistent with the induction of type I IFN responses by all three mildly pathogenic rickettsiae in THP-1 macrophages. The restrictive role of type I IFN in *R. parkeri* replication has recently been demonstrated in mouse BMDMs (24), with the authors elegantly showing that in this cell type, IFN-I production is mediated by the DNA sensor cyclic-GMP-AMP synthase (cGAS). Here, we demonstrate the higher abundance of many RLR pathway components by MS-based proteomics, which we further validated, by Western blotting for RIG-I and ISG15 (together with ISGylation), to be specifically induced by pathogenic species only. Moreover, and consistent with the fact that activation of the RLR pathway promotes elongation of the mitochondrial network (36, 37), we showed a significant expansion/fusion of the mitochondrial network footprint in THP-1 macrophages infected with the three pathogenic *Rickettsia* species. Combined, these results provide the first evidence for the activation of the RLR-dependent pathway for type I IFN production in human macrophages during rickettsial infections, indicating that mildly pathogenic rickettsiae may employ different pathways to stimulate IFN-I production. Evidence for cytosolic detection of RNA through RIG-I has been demonstrated for a much-reduced number of bacterial pathogens (68). Our results indicate that *R. parkeri*, R. africae, and *R. massiliae* should also be added to this group. However, the nature of the rickettsial ligands inducing RLR-mediated responses in macrophages, the contribution of the RLR pathway to type I interferon production during rickettsial infections, how repercussions of type I IFN production (through this or other pathways) differ between rickettsial species, and the possible existence of bacterial effectors targeting host IFN responses are several of the outstanding questions arising from our and others’ data (24, 69) that certainly mandate further studies into the interactions between rickettsiae and interferons.

**FIG 9:**
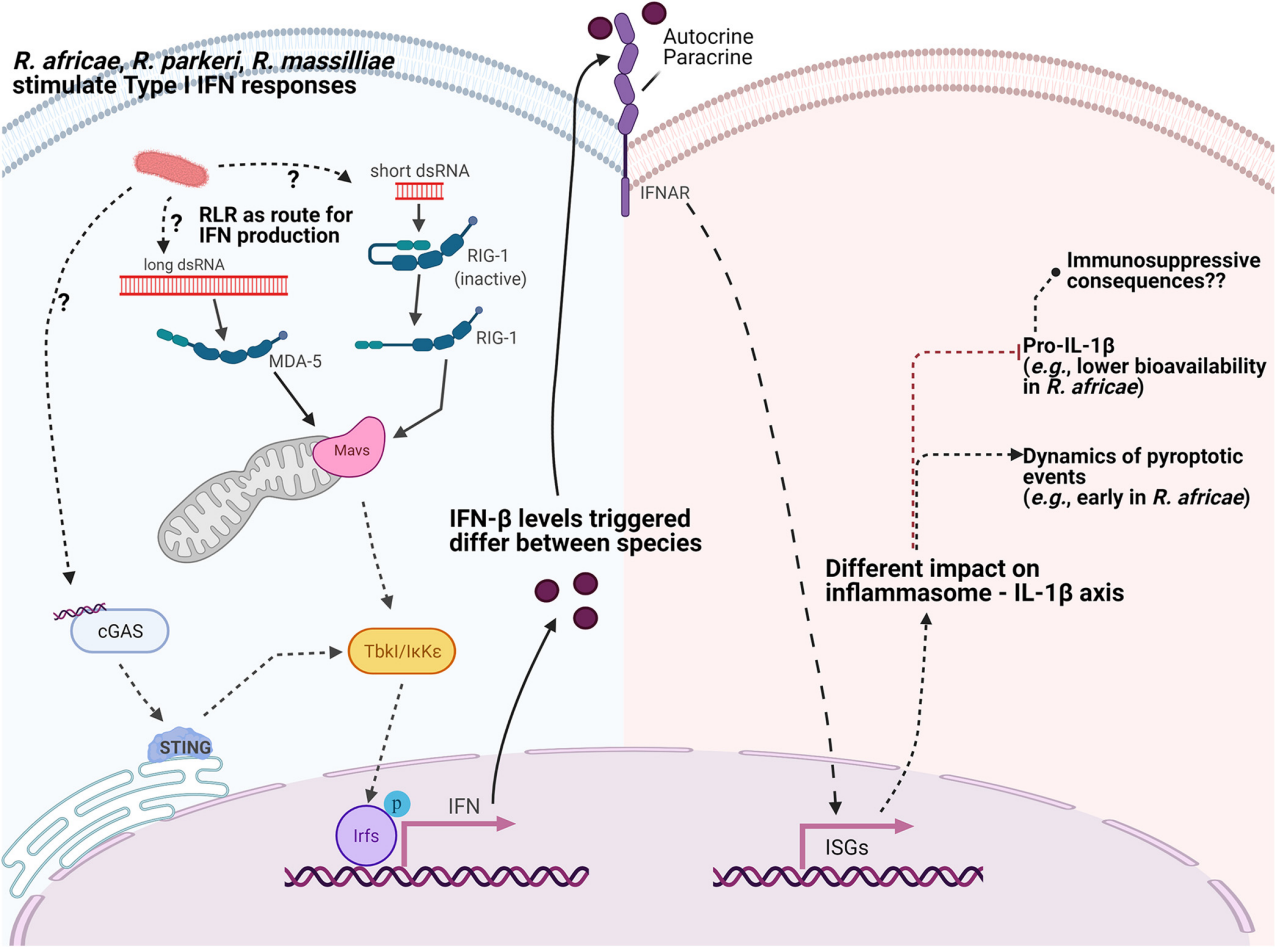
R. parkeri, R. africae, and R. massiliae-infected macrophages display differences in key inflammatory components. Our quantitative proteomics data indicate that all three species of mildly pathogenic rickettsiae stimulate type I interferon responses. Among the proteins with the highest increases in abundance upon infection are several members of the RIG-1-like receptor pathway (RLR), including the cytoplasmic RNA sensors RIG-I, MDA5, LGP2, and TRIM25 and ISG15. Therefore, RLR emerges as another possible route for IFN-1 production in *Rickettsia*-infected macrophages, raising exciting questions about the nature of the bacterial ligands triggering the MDA5 or RIG-1 sensors (highlighted with “?”). We further demonstrate mitochondrial fusion upon infection with all pathogenic species, consistent with the activation of the RLR pathway. These results also provide evidence that *Rickettsia* might induce IFN-I production by different routes, as shown by the identification of cGAS by Burke and colleagues in their work with mouse BMDMs (24). Although all three species triggered type I interferon responses, we have further demonstrated that the levels of IFN-β are different during the course of infection between species, with *R. africae* displaying higher levels at earlier times postinfection. These differences are likely to have a differential impact on the inflammasome–IL-1β axis between species, as we demonstrate that despite triggering earlier pyroptotic events, *R. africae* infection results in a lower bioavailability of the proinflammatory cytokine IL-β (both precursor and activated forms). We anticipate a possible link to IFN-β counterregulation. Differential regulation of IL-1β by *R. africae* opens exciting questions regarding its potential immunosuppressive repercussions, thereby enhancing *R. africae* pathogenesis. Figure created with bioRender.

Type I IFN and IL-1 cytokines represent distinct and specialized pathways of the innate inflammatory responses (39). Our results demonstrated decreased abundance of the proinflammatory IL-1β in *R. africae*-infected cells compared to the levels in *R. parkeri*- and R. massiliae-infected THP-1 macrophages at 24 hpi and consistently lower bioavailability of both precursor and mature forms of IL-1β in *R. africae*-infected cells across the infection. With an opposite trend, we observed more pyroptotic events in this infection condition at earlier time points postinfection. It was recently demonstrated that *R. parkeri* benefits from inflammasome-mediated host cell death to antagonize IFN-I (24). Moreover, it is also known that type I IFNs contribute to inflammasome-dependent caspase-1 activation, leading to proinflammatory pyroptotic cell death (70, 71). Therefore, our observations of earlier pyroptotic events in R. africae-infected cells could result from differences in IFN-I levels between infection conditions. Indeed, our results confirmed higher production of IFN-β in *R. africae*-infected THP-1 macrophages, thus demonstrating that rickettsial species trigger qualitatively and quantitatively distinct inflammatory responses during infection. Adding to this, the reduced bioavailability of IL-1β in *R. africae*-infected cells indicates a possible link to IFN-β counterregulation, as it has been demonstrated that during M. tuberculosis infections, IFN-β potently inhibits (in an IL-10-dependent manner) both IL-1α and IL-1β production (39, 72). Given the importance of IL-1β as an amplifier of immune reactions through the activation of innate immune cells, the differential regulation of this proinflammatory cytokine by *R. africae* opens exciting questions regarding the potential immunosuppressive repercussions of reducing IL-1 signaling for enhancing R. africae pathogenesis. The main differences in innate inflammatory components observed in this work, and possible responses associated with these changes, are summarized in Figure 9. Notably, in THP-1 macrophages infected with the highly pathogenic R. conorii, we did not observe this drastic increase in interferon-stimulated responses (23). We have further corroborated these observations by remining the SWATH files for three interferon-induced proteins (Mx1 [accession no. P20591], ISG15, and RIG-I), all significantly overrepresented upon infection with mildly pathogenic species. Further comparison of proteins commonly quantified in all data sets also revealed differential accumulation of various proteins involved in metabolic pathways and protein folding/quality control, evidencing additional distinctions in host cellular signatures resulting from infection with R. conorii compared with infection with the mildly pathogenic rickettsiae. The future elucidation of these different capacities to exploit immunometabolic pathways among *Rickettsia* species may contribute important molecular cues to distinguish between the severe and mild pathogenic conditions caused by different rickettsial species.

Overall, our data underline the complex and nuanced interactions between different rickettsial species and macrophages during infection, continuing to reveal additional layers of complexity between *Rickettsia* and host cells’ constant arms race for survival. We confirm species-specific interference with many host cellular processes and thereby provide new clues to understand the molecular basis for the long-known differences in pathogenicity among mildly pathogenic *Rickettsia* species.

## MATERIALS AND METHODS

### Cell lines and *Rickettsia* growth and purification.

Vero cells were grown in Dulbecco’s modified Eagle’s medium (DMEM; Gibco) supplemented with 10% heat-inactivated fetal bovine serum (Atlanta Biologicals), 1× nonessential amino acids (Corning), and 0.5 mM sodium pyruvate (Corning). THP-1 (ATCC TIB-202) cells were grown in RPMI 1640 medium (Gibco) supplemented with 10% heat-inactivated fetal bovine serum (Atlanta Biologicals). Differentiation of THP-1 cells into macrophage-like cells was carried out by the addition of 100 nM phorbol 12-myristate 13-acetate (PMA; Fisher). Cells were allowed to differentiate and adhere for 3 days prior to infection. Both cell lines were maintained in a humidified 5% CO_2_ incubator at 34°C. Rickettsia montanensis isolate M/5-6T, Rickettsia massiliae isolate MTU5, Rickettsia parkeri isolate Portsmouth, and Rickettsia africae isolate ESF-5 T were obtained from CSUR—Collection de Souches de l’Unité des Rickettsies, Marseille, France. All *Rickettsia* species were propagated in Vero cells and purified as previously described (73, 74).

### Assessment of *Rickettsia* growth dynamics.

Growth curves were performed by inoculating *R. parkeri*, *R. africae*, and *R. massiliae* at a MOI of 10 into PMA-differentiated THP-1 macrophages at a confluence of 2 × 10^5^ cells per well, in 24-well plates, with 2 wells infected for each day of the growth curve. Plates were centrifuged at 300 × *g* for 5 min at room temperature to induce contact between rickettsiae and host cells and incubated at 34°C and 5% CO_2_. At each specific time point postinoculation, cells were scraped and samples were stored in phosphate-buffered saline (PBS) at −80°C. Genomic DNA was extracted using the DNeasy blood and tissue kit (Qiagen) according to the manufacturer’s instructions. The extracted DNA was subjected to quantitative PCR analysis using the 7500 fast real-time PCR system (Thermo Fisher Scientific). Bacterial growth was queried by quantitative PCR using PowerUp SYBR green master mix (Applied Biosystems) under the following conditions: 2 min at 50°C and 2 min at 95°C, followed by 40 cycles of 95°C for 3 s and 58°C for 30 s. The rickettsial *sca1* gene was amplified using the primers sca1-F (5′-GGGTTCGATGCTGAAATCGAC-3′) and sca1-R (5′-GTCCGTAAATAGAAACCACATGAC-3′), and the mammalian *b2m* gene was amplified using the primers b2m-F (5′-CACTGAAAAAGATGAGTATGCC-3′) and b2m-R (5′-AACATTCCCTGACAATCCC-3′). Growth is presented as the ratio of *sca1* versus *b2m*. All unknowns were quantified by the cycle threshold method (ΔΔ*C_T_*) with comparison to molar standards. Experiments were done in triplicate.

Growth dynamics were also assessed by immunofluorescence. Briefly, PMA-differentiated THP-1 macrophage monolayers at a confluence of 2 × 10^5^ cells per well, in 24-well plates seeded onto glass coverslips, were inoculated with R. parkeri, R. africae, and R. massiliae at an MOI of 10. Plates were centrifuged at 300 × *g* for 5 min at room temperature to induce contact between rickettsiae and host cells and incubated at 34°C and 5% CO_2_ for 24, 48, and 72 h. At each indicated time point postinoculation, infected monolayers were washed with PBS and fixed in 4% paraformaldehyde (PFA) for 20 min. Samples were then permeabilized with 0.1% Triton X-100 and blocked with 2% bovine serum albumin (BSA). *Rickettsia* growth was assessed by staining with anti-*Rickettsia* polyclonal antibody NIH/RML I7198 (1:1,500), followed by Alexa Fluor 488-conjugated goat anti-rabbit IgG (1:1,000), DAPI (4′,6-diamidino-2-phenylindole; 1:1,000), and Texas Red–X phalloidin (1:200). After washing with PBS, glass coverslips were mounted in Mowiol mounting medium and preparations were viewed on a Zeiss Axiovert 200M fluorescence microscope (Carl Zeiss) using a final ×40 optical zoom and processed with ImageJ software.

### Sample preparation.

PMA-differentiated THP-1 cell monolayers at a cell confluence of 2 × 10^5^ cells per well, in 24-well plates (6 wells per condition), were infected with *R. parkeri*, *R. africae*, and *R. massiliae* at an MOI of 10 or maintained uninfected as a control. Plates were centrifuged at 300 × *g* for 5 min at room temperature to induce contact between rickettsiae and host cells and incubated at 34°C and 5% CO_2_ for 24 h. At the specified time point, the culture medium was removed, cells were washed 1× with PBS, and total protein was extracted using 100 μl of protein extraction buffer per well (25 mM Tris/HCl, 5 mM EDTA, 1% Triton X-100, and Pierce protease inhibitors [Thermo Fisher Scientific], pH 7.0). Samples were passed 10 times through an insulin syringe with a 28-gauge needle (Becton, Dickinson) and denatured using 6× SDS sample buffer (4× Tris/HCl, 30% glycerol, 10% SDS, 0.6 M dithiothreitol, 0.012% bromophenol blue, pH 6.8) during 10 min at 95°C. The total protein content in each sample was then quantified using the Pierce 660-nm protein assay kit (Thermo Fisher Scientific) and kept at −80°C until further processing. Experiments were done in quadruplicate. After thawing, amounts of 10 μg of each replicate sample from each experimental condition were pooled, creating the four pooled samples (*R. parkeri* pool, *R. africae* pool, *R. massiliae* pool, and uninfected pool). At this point, the same amount of a recombinant protein (green fluorescent protein fused to maltose-binding periplasmic protein [MalE-GFP]) was added to each replicate sample and the pooled samples to serve as an internal standard. All the samples were boiled for 5 min, and acrylamide was added as an alkylating agent.

### In-gel digestion and LC-MS/MS.

The volume corresponding to 40 μg of each replicate sample, as well as pooled samples, was then loaded into a precast gel (4 to 20% Mini-Protean TGX gel; Bio-Rad), and SDS-PAGE was partially run at 110 V (75). After SDS-PAGE, proteins were stained with colloidal Coomassie blue as previously described (76). The lanes were sliced into 3 fractions with a scalpel, and after the excision of the gel bands, each one was sliced into smaller pieces. The gel pieces were destained using a 50 mM ammonium bicarbonate solution with 30% acetonitrile (ACN), followed by a washing step with water (each step was performed in a thermomixer [Eppendorf] at 1,050 × rpm for 15 min). The gel pieces were dehydrated on a Concentrator plus/Vacufuge R plus (Eppendorf). To each gel band, 75 μl of trypsin (0.01 μg/μl solution in 10 mM ammonium bicarbonate) was added and the band left for 15 min at 4°C to rehydrate the gel. After this period, 75 μl of 10 mM ammonium bicarbonate was added and in-gel digestion was performed overnight at room temperature in the dark. After digestion, the excess solution from gel pieces was collected into a low-binding microcentrifuge tube (LoBind, Eppendorf) and peptides were extracted from the gel pieces by the sequential addition of three solutions of increasing percentages of ACN (30, 50, and 98%) in 1% formic acid (FA). After the addition of each solution, the gel pieces were shaken in a thermomixer (Eppendorf) at 1,250 rpm for 15 min and the solution was collected into the tube containing the previous fraction. The peptide mixtures were dried by rotary evaporation under vacuum (Concentrator plus/Vacufuge plus; Eppendorf). The peptides from each fraction of each sample were pooled for SWATH analysis, while the peptides from the pooled samples were kept separated in the three fractions of the digestion procedure. After digestion, all samples were subjected to solid-phase extraction with C18 sorbent (OMIX tip; Agilent Technologies). The eluted peptides were evaporated and solubilized in 30 μl mobile phase, aided by ultrasonication using a cup horn device (Vibra-Cell 750 W; Sonics) at 40% amplitude for 2 min. Samples were then centrifuged for 5 min at 14,100 × *g* (MiniSpin plus; Eppendorf) and analyzed by liquid chromatography coupled to tandem mass spectrometry (LC-MS/MS). The TripleTOF 6600 system (Sciex) was operated in two phases: information-dependent acquisition (IDA) of each fraction of the pooled samples, followed by SWATH (sequential windowed data-independent acquisition of the total high-resolution mass spectra) acquisition of each sample. Peptide separation was performed using liquid chromatography (nanoLC 425; Eksigent) on a Triart C_18_ capillary column 1/32 in (12 nm, S-3μm, 150 by 0.3 mm; YMC) and using a Triart C18 capillary guard column (0.5 by 5 mm, 3 μm, 12 nm; YMC) at 50°C at 5 μl/min with a 50-min gradient from 5% to 30% ACN in 0.1% FA in a total run time of 65 min, and the peptides were eluted into the mass spectrometer using an electrospray ionization source (DuoSpray source; Sciex). IDA experiments were performed by analyzing each fraction of the pooled samples. The mass spectrometer was set for IDA scanning full spectra (350 to 2,250 *m/z*) for 250 ms, followed by 50 MS/MS scans (100 to 1,500 *m/z* with an accumulation time of 60 ms in order to maintain a cycle time of 3.3 s). Candidate ions with a charge state between +1 and +5 and counts above a minimum threshold of 100 cps were isolated for fragmentation, and one MS/MS spectrum was collected before adding those ions to the exclusion list for 15 s (mass spectrometer operated by Analyst TF 1.8; Sciex). Rolling collision energy was used with a collision energy spread (CES) of 5. The SWATH setup was essentially as described previously (77), with the same chromatographic conditions used for SWATH and IDA acquisitions. For SWATH-MS-based experiments, the mass spectrometer was operated in a looped product ion mode. The SWATH-MS setup was designed specifically for the samples to be analyzed (Table S7), in order to adapt the SWATH windows to the complexity of this batch of samples. A set of 168 windows of various widths (containing 1 *m/z* for window overlap) was constructed covering the precursor mass range of 350 to 2,250 *m/z*. A 500-ms survey scan (350 to 2,250 *m/z*) was acquired at the beginning of each cycle for instrument calibration, and SWATH-MS/MS spectra were collected from 100 to 2,250 *m/z* for 19 ms, resulting in a cycle time of 3.76 s from the precursors ranging from 350 to 2,250 *m/z*. The collision energy for each window was determined according to the calculation for a charge +2 ion centered upon the window, with varying collision energy spread according to the window.

### Protein identification and relative quantification.

A specific library of precursor masses and fragment ions was created by combining all files from the IDA experiments and used for subsequent SWATH processing. The library was obtained using Protein Pilot software (version 5.0.1; Sciex) with the following search parameters: Homo sapiens Swiss-Prot database (downloaded in September 2020) and MalE-GFP, acrylamide-alkylated cysteines as fixed modification, and the gel-based special focus option. An independent false discovery rate (FDR) analysis using the target-decoy approach provided with the Protein Pilot software was used to assess the quality of the identifications, and identifications were considered positive when identified proteins and peptides reached a 5% local FDR (78, 79). Data processing was performed using the SWATH processing plug-in for PeakView (version 2.2; Sciex). Briefly, peptides were selected from the library using the following criteria: (i) the unique peptides for a specific targeted protein were ranked by the intensity of the precursor ion from the IDA analysis as estimated by the Protein Pilot software, and (ii) peptides that contained biological modifications and/or were shared between different protein entries/isoforms were excluded from selection. Up to 15 peptides were chosen per protein, and SWATH quantitation was attempted for all proteins in the library file that were identified below 5% local FDR from Protein Pilot searches. Peptide retention times were adjusted by using the MalE-GFP peptides. In SWATH acquisition data, peptides are confirmed by finding and scoring peak groups, which are a set of fragment ions for the peptide. Up to 5 target fragment ions were automatically selected, and the peak groups were scored following the criteria described in reference 80. The peak group confidence threshold was determined based on an FDR analysis using the target-decoy approach, and a 1% extraction FDR threshold was used for all the analyses. Peptides that met the 1% FDR threshold in at least three replicates of a given experimental group were retained, and the peak areas of the target fragment ions of those peptides were extracted across the experiments using an extracted-ion chromatogram window of 4 min. Protein levels were estimated by summing all the transitions from all the peptides for a given protein (81) and normalized to the total intensity at the protein level. Statistical tests were performed in RStudio using the nonparametric Kruskal-Wallis test for multiple hypothesis testing, followed by the *post hoc* Dunn’s test and adjusting the *P* values for multiple testing by controlling the FDR using the Benjamini-Hochberg adjustment; proteins were considered altered when an alteration in abundance of at least 20% (fold change of ≤0.83 or ≥1.2) was observed between experimental conditions.

### Bioinformatics analysis.

Functional protein association networks were evaluated using the Search Tool for Retrieval of Interacting Genes/Proteins (STRING) 10.5 (http://string-db.org/) using the highest confidence score (0.9) and the Markov cluster algorithm (MCL) with an inflation parameter of 3 (31). The GO Enrichment Plot function in Image GP (http://www.ehbio.com/ImageGP/) was used to integrate and represent the Gene Ontology analysis.

### Western blotting.

After thawing, the same amount of protein for each sample was resolved by SDS-PAGE using 12.5% polyacrylamide gels in a Bio-Rad Mini-Protean tetra cell and transferred to a polyvinylidene difluoride membrane at 100 V during 100 min at 4°C. The membranes were blocked for 60 min with 2% BSA in Tris-buffered saline (TBS) containing 0.1% Tween 20 and then incubated at 4°C overnight with primary antibodies. The following primary antibodies were used accordingly: anti-RIG-I (D-12) antibody (sc-376845; Santa Cruz Biotechnology) (1:100), anti-ISG15 antibody (F-9) (sc-166755; Santa Cruz Biotechnology) (1:100), anti-IL-1β (D3U3E) rabbit monoclonal antibody (MAb) (12703; Cell Signaling Technology) (1:1,000), anti-cleaved IL-1β (Asp116) (D3A3Z) rabbit MAb (83186; Cell Signaling Technology) (1:1,000), anti-caspase-1 (D7F10) rabbit MAb (3866; Cell Signaling Technology) (1:1,000), anti-gasdermin D (E8G3F) rabbit MAb (97558; Cell Signaling Technology) (1:1,000), anti-cleaved gasdermin D (Asp275) (E7H9G) rabbit MAb (36425; Cell Signaling Technology) (1:1,000), and anti-HMGB1 (D3E5) rabbit MAb (6893; Cell Signaling Technology) (1:1,000). After several washes with TBS-T (TBS containing 0.1% Tween 20), the membranes were incubated at room temperature with the following corresponding secondary antibodies: anti-mouse IgG (whole molecule)–peroxidase produced in rabbit (A9044; Sigma) (1:10,000) and anti-rabbit IgG, horseradish peroxidase (HRP) linked, produced in goat (7074; Cell Signaling Technology) (1:10,000). The membranes were washed again in TBS-T and visualized using NZY supreme ECL HRP substrate (NZYTech) on a VWR Imager.

The membranes were stained using SERVA purple (SERVA electrophoresis; Enzo) to determine the total protein load in each lane according to the manufacturer’s guidelines. The integrated density of antibody-stained protein bands and total protein content of each lane were measured using ImageJ software.

### Analysis of alterations in mitochondrial morphology.

PMA-differentiated THP-1 macrophage monolayers at a confluence of 2 × 10^5^ cells per well, in 24-well plates seeded onto glass coverslips, were inoculated with R. montanensis, R. parkeri, R. africae, and R. massiliae at an MOI of 10 or maintained uninfected as a control. Plates were centrifuged at 300 × *g* for 5 min at room temperature to induce contact between rickettsiae and host cells and incubated at 34°C and 5% CO_2_ for 24 h. At each indicated time point postinoculation, infected monolayers were washed with PBS and fixed in 4% PFA for 20 min. Samples were then permeabilized with 0.1% Triton X-100 and blocked with 2% BSA. Labeling was carried out by incubation with anti-*Rickettsia* polyclonal antibody NIH/RML I7198 (1:1,500), followed by Alexa Fluor 568-conjugated goat anti-rabbit IgG (1:1,000), anti-Tom20 antibody (F-10) (sc-17764) (1:500) followed by Alexa Fluor 647 goat anti-mouse antibody (1:1,000), and DAPI (1:1,000). After washing with PBS, glass coverslips were mounted in Mowiol mounting medium and preparations were viewed on an LSM 710 Axio Observer Z1-based laser scanning confocal microscope (Carl Zeiss) equipped with a Plan-Apochromat 40×/1.40 objective and the following lasers: diode 405-30, argon/2, DPSS 561-10, and HeNe633. Confocal images of cells were taken from various fields of view randomly selected across the entire coverslip area, and mitochondrial morphology was analyzed using the semiautomated morphometric tool MiNA within Fiji (38). Mitochondrial networks (labeled with anti-Tom20 antibody) from individual cells were selected and digitally isolated. From the output data, we used the values listed under “mitochondrial footprint” and those under “mean branch length.” A minimum of 50 cells were analyzed per condition, in a total of three independent experiments.

### Quantification of IFN-β levels.

Culture supernatants of uninfected and *Rickettsia*-infected THP-1 macrophages at the respective time points postinfection were collected and filtered with sterile 0.2-μm syringe filters (Whatman Puradisc; Cytiva), and IFN-β levels were measured with the human IFN-beta DuoSet enzyme-linked immunosorbent assay (ELISA) kit (R&D Systems), following the manufacturer’s instructions. Protein concentrations were determined based on the optical density, using a BioTek Cytation 3 imaging reader at 450 nm, and compared to standard curves for purified IFN-β (R&D Systems).

### Statistical analysis.

Statistical analysis and significance are reported in the figure legends. If not described otherwise, statistical analysis was performed using GraphPad Prism version 8.0.1 (GraphPad Software, Inc., CA, USA). Statistical analyses of the proteomics results are specifically described in the corresponding sections in Materials and Methods.

### Data availability.

The mass spectrometry proteomics data have been deposited to the ProteomeXchange Consortium via the PRIDE (82) partner repository with the data set identifier PXD026982.

## References

[1] Parola P, Paddock CD, Socolovschi C, Labruna MB, Mediannikov O, Kernif T, Abdad MY, Stenos J, Bitam I, Fournier PE, Raoult D. 2013. Update on tick-borne rickettsioses around the world: a geographic approach. Clin Microbiol Rev 26:657–702. doi:10.1128/CMR.00032-13. 24092850 PMC3811236

[2] El-Sayed A, Kamel M. 2020. Climatic changes and their role in emergence and re-emergence of diseases. Environ Sci Pollut Res Int 27:22336–22352. doi:10.1007/s11356-020-08896-w. 32347486 PMC7187803

[3] Bouchard C, Dibernardo A, Koffi J, Wood H, Leighton PA, Lindsay LR. 2019. N Increased risk of tick-borne diseases with climate and environmental changes. Can Commun Dis Rep 45:83–89. doi:10.14745/ccdr.v45i04a02. 31285697 PMC6587693

[4] Tomassone L, Portillo A, Novakova M, de Sousa R, Oteo JA. 2018. Neglected aspects of tick-borne rickettsioses. Parasit Vectors 11:263. doi:10.1186/s13071-018-2856-y. 29690900 PMC5937841

[5] Parola P, Socolovschi C, Jeanjean L, Bitam I, Fournier PE, Sotto A, Labauge P, Raoult D. 2008. Warmer weather linked to tick attack and emergence of severe rickettsioses. PLoS Negl Trop Dis 2:e338. doi:10.1371/journal.pntd.0000338. 19015724 PMC2581602

[6] Sahni A, Fang R, Sahni SK, Walker DH. 2019. Pathogenesis of rickettsial diseases: pathogenic and immune mechanisms of an endotheliotropic infection. Annu Rev Pathol 14:127–152. doi:10.1146/annurev-pathmechdis-012418-012800. 30148688 PMC6505701

[7] Fang R, Blanton LS, Walker DH. 2017. Rickettsiae as emerging infectious agents. Clin Lab Med 37:383–400. doi:10.1016/j.cll.2017.01.009. 28457356

[8] Alvarez-Hernandez G, Roldan JFG, Milan NSH, Lash RR, Behravesh CB, Paddock CD. 2017. Rocky Mountain spotted fever in Mexico: past, present, and future. Lancet Infect Dis 17:e189–e196. doi:10.1016/S1473-3099(17)30173-1. 28365226

[9] McQuiston JH, Zemtsova G, Perniciaro J, Hutson M, Singleton J, Nicholson WL, Levin ML. 2012. Afebrile spotted fever group Rickettsia infection after a bite from a Dermacentor variabilis tick infected with Rickettsia montanensis. Vector Borne Zoonotic Dis 12:1059–1061. doi:10.1089/vbz.2012.1078. 23153005 PMC4699432

[10] Paddock CD, Finley RW, Wright CS, Robinson HN, Schrodt BJ, Lane CC, Ekenna O, Blass MA, Tamminga CL, Ohl CA, McLellan SL, Goddard J, Holman RC, Openshaw JJ, Sumner JW, Zaki SR, Eremeeva ME. 2008. Rickettsia parkeri rickettsiosis and its clinical distinction from Rocky Mountain spotted fever. Clin Infect Dis 47:1188–1196. doi:10.1086/592254. 18808353

[11] Tsai YS, Wu YH, Kao PT, Lin YC. 2008. African tick bite fever. J Formos Med Assoc 107:73–76. doi:10.1016/S0929-6646(08)60011-X. 18218581

[12] Raoult D, Fournier PE, Fenollar F, Jensenius M, Prioe T, de Pina JJ, Caruso G, Jones N, Laferl H, Rosenblatt JE, Marrie TJ. 2001. Rickettsia africae, a tick-borne pathogen in travelers to sub-Saharan Africa. N Engl J Med 344:1504–1510. doi:10.1056/NEJM200105173442003. 11357153

[13] Cascio A, Torina A, Valenzise M, Blanda V, Camarda N, Bombaci S, Iaria C, De Luca F, Wasniewska M. 2013. Scalp eschar and neck lymphadenopathy caused by Rickettsia massiliae. Emerg Infect Dis 19:836–837. doi:10.3201/eid1905.121169. 23697545 PMC3647502

[14] Vitale G, Mansuelo S, Rolain JM, Raoult D. 2006. Rickettsia massiliae human isolation. Emerg Infect Dis 12:174–175. doi:10.3201/eid1201.050850. 16634183 PMC3291392

[15] European Centre for Disease Prevention and Control. 2013. Epidemiological situation of rickettsioses in EU/EFTA countries. ECDC, Stockholm, Sweden.

[16] Riley SP, Fish AI, Garza DA, Banajee KH, Harris EK, Del Piero F, Martinez JJ. 2016. Nonselective persistence of a Rickettsia conorii extrachromosomal plasmid during mammalian infection. Infect Immun 84:790–797. doi:10.1128/IAI.01205-15. 26755154 PMC4771360

[17] Banajee KH, Embers ME, Langohr IM, Doyle LA, Hasenkampf NR, Macaluso KR. 2015. Amblyomma maculatum feeding augments Rickettsia parkeri infection in a rhesus macaque model: a pilot study. PLoS One 10:e0135175. doi:10.1371/journal.pone.0135175. 26244337 PMC4526656

[18] Price JV, Vance RE. 2014. The macrophage paradox. Immunity 41:685–693. doi:10.1016/j.immuni.2014.10.015. 25517611

[19] Curto P, Simoes I, Riley SP, Martinez JJ. 2016. Differences in intracellular fate of two spotted fever group Rickettsia in macrophage-like cells. Front Cell Infect Microbiol 6:80. doi:10.3389/fcimb.2016.00080. 27525249 PMC4965480

[20] Engström P, Burke TP, Mitchell G, Ingabire N, Mark KG, Golovkine G, Iavarone AT, Rape M, Cox JS, Welch MD. 2019. Evasion of autophagy mediated by Rickettsia surface protein OmpB is critical for virulence. Nat Microbiol 4:2538–2551. doi:10.1038/s41564-019-0583-6. 31611642 PMC6988571

[21] Narra HP, Sahni A, Walker DH, Sahni SK. 2020. Recent research milestones in the pathogenesis of human rickettsioses and opportunities ahead. Future Microbiol 15:753–765. doi:10.2217/fmb-2019-0266. 32691620 PMC7787141

[22] Curto P, Riley SP, Simoes I, Martinez JJ. 2019. Macrophages infected by a pathogen and a non-pathogen spotted fever group Rickettsia reveal differential reprogramming signatures early in infection. Front Cell Infect Microbiol 9:97. doi:10.3389/fcimb.2019.00097. 31024862 PMC6467950

[23] Curto P, Santa C, Allen P, Manadas B, Simoes I, Martinez JJ. 2019. A pathogen and a non-pathogen spotted fever group Rickettsia trigger differential proteome signatures in macrophages. Front Cell Infect Microbiol 9:43. doi:10.3389/fcimb.2019.00043. 30895174 PMC6414445

[24] Burke TP, Engstrom P, Chavez RA, Fonbuena JA, Vance RE, Welch MD. 2020. Inflammasome-mediated antagonism of type I interferon enhances Rickettsia pathogenesis. Nat Microbiol 5:688–696. doi:10.1038/s41564-020-0673-5. 32123346 PMC7239376

[25] Jean Beltran PM, Federspiel JD, Sheng X, Cristea IM. 2017. Proteomics and integrative omic approaches for understanding host-pathogen interactions and infectious diseases. Mol Syst Biol 13:922. doi:10.15252/msb.20167062. 28348067 PMC5371729

[26] Krey K, Babnis AW, Pichlmair A. 2020. System-based approaches to delineate the antiviral innate immune landscape. Viruses 12:1196. doi:10.3390/v12101196. PMC7589202 33096788

[27] Yeung A, Hale C, Clare S, Palmer S, Bartholdson Scott J, Baker S, Dougan G. 2019. Using a systems biology approach to study host-pathogen interactions. Microbiol Spectr 7:7.2.27. doi:10.1128/microbiolspec.BAI-0021-2019. PMC11590422 30953425

[28] Kristof MN, Allen PE, Yutzy LD, Thibodaux B, Paddock CD, Martinez JJ. 2021. Significant growth by Rickettsia species within human macrophage-like cells is a phenotype correlated with the ability to cause disease in mammals. Pathogens 10:228. doi:10.3390/pathogens10020228. 33669499 PMC7934685

[29] Rukmangadachar LA, Makharia GK, Mishra A, Das P, Hariprasad G, Srinivasan A, Gupta SD, Ahuja V, Acharya SK. 2016. Proteome analysis of the macroscopically affected colonic mucosa of Crohn’s disease and intestinal tuberculosis. Sci Rep 6:23162. doi:10.1038/srep23162. 26988818 PMC4796817

[30] Bussey KA, Lau U, Schumann S, Gallo A, Osbelt L, Stempel M, Arnold C, Wissing J, Gad HH, Hartmann R, Brune W, Jansch L, Whitehouse A, Brinkmann MM. 2018. The interferon-stimulated gene product oligoadenylate synthetase-like protein enhances replication of Kaposi’s sarcoma-associated herpesvirus (KSHV) and interacts with the KSHV ORF20 protein. PLoS Pathog 14:e1006937. doi:10.1371/journal.ppat.1006937. 29499066 PMC5851652

[31] Szklarczyk D, Morris JH, Cook H, Kuhn M, Wyder S, Simonovic M, Santos A, Doncheva NT, Roth A, Bork P, Jensen LJ, von Mering C. 2017. The STRING database in 2017: quality-controlled protein-protein association networks, made broadly accessible. Nucleic Acids Res 45:D362–D368. doi:10.1093/nar/gkw937. 27924014 PMC5210637

[32] Serio AW, Jeng RL, Haglund CM, Reed SC, Welch MD. 2010. Defining a core set of actin cytoskeletal proteins critical for actin-based motility of Rickettsia. Cell Host Microbe 7:388–398. doi:10.1016/j.chom.2010.04.008. 20478540 PMC2935136

[33] Rehwinkel J, Gack MU. 2020. RIG-I-like receptors: their regulation and roles in RNA sensing. Nat Rev Immunol 20:537–551. doi:10.1038/s41577-020-0288-3. 32203325 PMC7094958

[34] Dixit E, Kagan JC. 2013. Intracellular pathogen detection by RIG-I-like receptors. Adv Immunol 117:99–125. doi:10.1016/B978-0-12-410524-9.00004-9. 23611287 PMC3947775

[35] Diao F, Li S, Tian Y, Zhang M, Xu LG, Zhang Y, Wang RP, Chen D, Zhai Z, Zhong B, Tien P, Shu HB. 2007. Negative regulation of MDA5- but not RIG-I-mediated innate antiviral signaling by the dihydroxyacetone kinase. Proc Natl Acad Sci USA 104:11706–11711. doi:10.1073/pnas.0700544104. 17600090 PMC1913852

[36] Pourcelot M, Arnoult D. 2014. Mitochondrial dynamics and the innate antiviral immune response. FEBS J 281:3791–3802. doi:10.1111/febs.12940. 25051991

[37] Castanier C, Garcin D, Vazquez A, Arnoult D. 2010. Mitochondrial dynamics regulate the RIG-I-like receptor antiviral pathway. EMBO Rep 11:133–138. doi:10.1038/embor.2009.258. 20019757 PMC2828750

[38] Valente AJ, Maddalena LA, Robb EL, Moradi F, Stuart JA. 2017. A simple ImageJ macro tool for analyzing mitochondrial network morphology in mammalian cell culture. Acta Histochem 119:315–326. doi:10.1016/j.acthis.2017.03.001. 28314612

[39] Mayer-Barber KD, Yan B. 2017. Clash of the cytokine titans: counter-regulation of interleukin-1 and type I interferon-mediated inflammatory responses. Cell Mol Immunol 14:22–35. doi:10.1038/cmi.2016.25. 27264686 PMC5214938

[40] Xue Y, Enosi Tuipulotu D, Tan WH, Kay C, Man SM. 2019. Emerging activators and regulators of inflammasomes and pyroptosis. Trends Immunol 40:1035–1052. doi:10.1016/j.it.2019.09.005. 31662274

[41] Frank D, Vince JE. 2019. Pyroptosis versus necroptosis: similarities, differences, and crosstalk. Cell Death Differ 26:99–114. doi:10.1038/s41418-018-0212-6. 30341423 PMC6294779

[42] McNab F, Mayer-Barber K, Sher A, Wack A, O’Garra A. 2015. Type I interferons in infectious disease. Nat Rev Immunol 15:87–103. doi:10.1038/nri3787. 25614319 PMC7162685

[43] Guarda G, Braun M, Staehli F, Tardivel A, Mattmann C, Forster I, Farlik M, Decker T, Du Pasquier RA, Romero P, Tschopp J. 2011. Type I interferon inhibits interleukin-1 production and inflammasome activation. Immunity 34:213–223. doi:10.1016/j.immuni.2011.02.006. 21349431

[44] Szulc-Dąbrowska L, Bossowska-Nowicka M, Struzik J, Toka FN. 2020. Cathepsins in bacteria-macrophage interaction: defenders or victims of circumstance? Front Cell Infect Microbiol 10:601072. doi:10.3389/fcimb.2020.601072. 33344265 PMC7746538

[45] Sharma T, Grover S, Arora N, P M, Ehtesham NZ, Hasnain SE. 2020. PGRS domain of Rv0297 of Mycobacterium tuberculosis is involved in modulation of macrophage functions to favor bacterial persistence. Front Cell Infect Microbiol 10:451. doi:10.3389/fcimb.2020.00451. 33042856 PMC7517703

[46] Pires D, Bernard EM, Pombo JP, Carmo N, Fialho C, Gutierrez MG, Bettencourt P, Anes E. 2017. Mycobacterium tuberculosis modulates miR-106b-5p to control cathepsin S expression resulting in higher pathogen survival and poor T-cell activation. Front Immunol 8:1819. doi:10.3389/fimmu.2017.01819. 29326705 PMC5741618

[47] Pires D, Marques J, Pombo JP, Carmo N, Bettencourt P, Neyrolles O, Lugo-Villarino G, Anes E. 2016. Role of cathepsins in Mycobacterium tuberculosis survival in human macrophages. Sci Rep 6:32247. doi:10.1038/srep32247. 27572605 PMC5004184

[48] Rivera-Marrero CA, Stewart J, Shafer WM, Roman J. 2004. The down-regulation of cathepsin G in THP-1 monocytes after infection with Mycobacterium tuberculosis is associated with increased intracellular survival of bacilli. Infect Immun 72:5712–5721. doi:10.1128/IAI.72.10.5712-5721.2004. 15385470 PMC517540

[49] Chauhan K, Kalam H, Dutt R, Kumar D. 2019. RNA splicing: a new paradigm in host-pathogen interactions. J Mol Biol 431:1565–1575. doi:10.1016/j.jmb.2019.03.001. 30857970 PMC7115970

[50] Boudreault S, Roy P, Lemay G, Bisaillon M. 2019. Viral modulation of cellular RNA alternative splicing: a new key player in virus-host interactions? Wiley Interdiscip Rev RNA 10:e1543. doi:10.1002/wrna.1543. 31034770 PMC6767064

[51] Dowling D, Nasr-Esfahani S, Tan CH, O’Brien K, Howard JL, Jans DA, Purcell DF, Stoltzfus CM, Sonza S. 2008. HIV-1 infection induces changes in expression of cellular splicing factors that regulate alternative viral splicing and virus production in macrophages. Retrovirology 5:18. doi:10.1186/1742-4690-5-18. 18241354 PMC2267807

[52] Pai AA, Baharian G, Page Sabourin A, Brinkworth JF, Nedelec Y, Foley JW, Grenier JC, Siddle KJ, Dumaine A, Yotova V, Johnson ZP, Lanford RE, Burge CB, Barreiro LB. 2016. Widespread shortening of 3′ untranslated regions and increased exon inclusion are evolutionarily conserved features of innate immune responses to infection. PLoS Genet 12:e1006338. doi:10.1371/journal.pgen.1006338. 27690314 PMC5045211

[53] Kalam H, Fontana MF, Kumar D. 2017. Alternate splicing of transcripts shape macrophage response to Mycobacterium tuberculosis infection. PLoS Pathog 13:e1006236. doi:10.1371/journal.ppat.1006236. 28257432 PMC5352146

[54] Mohr I, Sonenberg N. 2012. Host translation at the nexus of infection and immunity. Cell Host Microbe 12:470–483. doi:10.1016/j.chom.2012.09.006. 23084916 PMC7104986

[55] Jaafar ZA, Kieft JS. 2019. Viral RNA structure-based strategies to manipulate translation. Nat Rev Microbiol 17:110–123. doi:10.1038/s41579-018-0117-x. 30514982 PMC6452865

[56] Ohmer M, Tzivelekidis T, Niedenfuhr N, Volceanov-Hahn L, Barth S, Vier J, Borries M, Busch H, Kook L, Biniossek ML, Schilling O, Kirschnek S, Hacker G. 2019. Infection of HeLa cells with Chlamydia trachomatis inhibits protein synthesis and causes multiple changes to host cell pathways. Cell Microbiol 21:e12993. doi:10.1111/cmi.12993. 30551267

[57] Lemaitre B, Girardin SE. 2013. Translation inhibition and metabolic stress pathways in the host response to bacterial pathogens. Nat Rev Microbiol 11:365–369. doi:10.1038/nrmicro3029. 23669888

[58] Grobler Y, Yun CY, Kahler DJ, Bergman CM, Lee H, Oliver B, Lehmann R. 2018. Whole genome screen reveals a novel relationship between Wolbachia levels and Drosophila host translation. PLoS Pathog 14:e1007445. doi:10.1371/journal.ppat.1007445. 30422992 PMC6258568

[59] Perales C, Carrasco L, Ventoso I. 2003. Cleavage of eIF4G by HIV-1 protease: effects on translation. FEBS Lett 533:89–94. doi:10.1016/s0014-5793(02)03764-x. 12505164

[60] Ventoso I, Blanco R, Perales C, Carrasco L. 2001. HIV-1 protease cleaves eukaryotic initiation factor 4G and inhibits cap-dependent translation. Proc Natl Acad Sci USA 98:12966–12971. doi:10.1073/pnas.231343498. 11606767 PMC60808

[61] Ohlmann T, Prevot D, Decimo D, Roux F, Garin J, Morley SJ, Darlix JL. 2002. In vitro cleavage of eIF4GI but not eIF4GII by HIV-1 protease and its effects on translation in the rabbit reticulocyte lysate system. J Mol Biol 318:9–20. doi:10.1016/S0022-2836(02)00070-0. 12054764

[62] Jager S, Cimermancic P, Gulbahce N, Johnson JR, McGovern KE, Clarke SC, Shales M, Mercenne G, Pache L, Li K, Hernandez H, Jang GM, Roth SL, Akiva E, Marlett J, Stephens M, D’Orso I, Fernandes J, Fahey M, Mahon C, O’Donoghue AJ, Todorovic A, Morris JH, Maltby DA, Alber T, Cagney G, Bushman FD, Young JA, Chanda SK, Sundquist WI, Kortemme T, Hernandez RD, Craik CS, Burlingame A, Sali A, Frankel AD, Krogan NJ. 2011. Global landscape of HIV-human protein complexes. Nature 481:365–370. doi:10.1038/nature10719. 22190034 PMC3310911

[63] Pan Y, Zhang ZN, Yin LB, Fu YJ, Jiang YJ, Shang H. 2019. Reduced eIF3d accelerates HIV disease progression by attenuating CD8+ T cell function. J Transl Med 17:167. doi:10.1186/s12967-019-1925-0. 31118081 PMC6530059

[64] Li M, Gustchina A, Cruz R, Simoes M, Curto P, Martinez J, Faro C, Simoes I, Wlodawer A. 2015. Structure of RC1339/APRc from Rickettsia conorii, a retropepsin-like aspartic protease. Acta Crystallogr D Biol Crystallogr 71:2109–2118. doi:10.1107/S1399004715013905. 26457434 PMC4601372

[65] Cruz R, Huesgen P, Riley SP, Wlodawer A, Faro C, Overall CM, Martinez JJ, Simoes I. 2014. RC1339/APRc from Rickettsia conorii is a novel aspartic protease with properties of retropepsin-like enzymes. PLoS Pathog 10:e1004324. doi:10.1371/journal.ppat.1004324. 25144529 PMC4140852

[66] Ngo CC, Man SM. 2017. Mechanisms and functions of guanylate-binding proteins and related interferon-inducible GTPases: roles in intracellular lysis of pathogens. Cell Microbiol 19:e12791. doi:10.1111/cmi.12791. 28975702

[67] Schwartz SL, Conn GL. 2019. RNA regulation of the antiviral protein 2′-5′-oligoadenylate synthetase. Wiley Interdiscip Rev RNA 10:e1534. doi:10.1002/wrna.1534. 30989826 PMC6585406

[68] Snyder DT, Hedges JF, Jutila MA. 2017. Getting “inside” type I IFNs: type I IFNs in intracellular bacterial infections. J Immunol Res 2017:9361802. doi:10.1155/2017/9361802. 28529959 PMC5424489

[69] Bechelli J, Rumfield CS, Walker DH, Widen S, Khanipov K, Fang R. 2021. Subversion of host innate immunity by Rickettsia australis via a modified autophagic response in macrophages. Front Immunol 12:638469. doi:10.3389/fimmu.2021.638469. 33912163 PMC8071864

[70] Malireddi RK, Kanneganti TD. 2013. Role of type I interferons in inflammasome activation, cell death, and disease during microbial infection. Front Cell Infect Microbiol 3:77. doi:10.3389/fcimb.2013.00077. 24273750 PMC3824101

[71] Labzin LI, Lauterbach MA, Latz E. 2016. Interferons and inflammasomes: cooperation and counterregulation in disease. J Allergy Clin Immunol 138:37–46. doi:10.1016/j.jaci.2016.05.010. 27373324

[72] Mayer-Barber KD, Andrade BB, Barber DL, Hieny S, Feng CG, Caspar P, Oland S, Gordon S, Sher A. 2011. Innate and adaptive interferons suppress IL-1alpha and IL-1beta production by distinct pulmonary myeloid subsets during Mycobacterium tuberculosis infection. Immunity 35:1023–1034. doi:10.1016/j.immuni.2011.12.002. 22195750 PMC3246221

[73] Ammerman NC, Beier-Sexton M, Azad AF. 2008. Laboratory maintenance of Rickettsia rickettsii. Curr Protoc Microbiol Chapter 3:Unit 3A.5. doi:10.1002/9780471729259.mc03a05s11. PMC2725428 19016440

[74] Chan YG, Riley SP, Chen E, Martinez JJ. 2011. Molecular basis of immunity to rickettsial infection conferred through outer membrane protein B. Infect Immun 79:2303–2313. doi:10.1128/IAI.01324-10. 21444665 PMC3125829

[75] Anjo SI, Santa C, Manadas B. 2015. Short GeLC-SWATH: a fast and reliable quantitative approach for proteomic screenings. Proteomics 15:757–762. doi:10.1002/pmic.201400221. 25418953

[76] Manadas B, Santos AR, Szabadfi K, Gomes JR, Garbis SD, Fountoulakis M, Duarte CB. 2009. BDNF-induced changes in the expression of the translation machinery in hippocampal neurons: protein levels and dendritic mRNA. J Proteome Res 8:4536–4552. doi:10.1021/pr900366x. 19702335

[77] Gillet LC, Navarro P, Tate S, Rost H, Selevsek N, Reiter L, Bonner R, Aebersold R. 2012. Targeted data extraction of the MS/MS spectra generated by data-independent acquisition: a new concept for consistent and accurate proteome analysis. Mol Cell Proteomics 11:O111.016717. doi:10.1074/mcp.O111.016717. PMC3433915 22261725

[78] Tang WH, Shilov IV, Seymour SL. 2008. Nonlinear fitting method for determining local false discovery rates from decoy database searches. J Proteome Res 7:3661–3667. doi:10.1021/pr070492f. 18700793

[79] Sennels L, Bukowski-Wills JC, Rappsilber J. 2009. Improved results in proteomics by use of local and peptide-class specific false discovery rates. BMC Bioinformatics 10:179. doi:10.1186/1471-2105-10-179. 19523214 PMC2709624

[80] Lambert JP, Ivosev G, Couzens AL, Larsen B, Taipale M, Lin ZY, Zhong Q, Lindquist S, Vidal M, Aebersold R, Pawson T, Bonner R, Tate S, Gingras AC. 2013. Mapping differential interactomes by affinity purification coupled with data-independent mass spectrometry acquisition. Nat Methods 10:1239–1245. doi:10.1038/nmeth.2702. 24162924 PMC3882083

[81] Collins BC, Gillet LC, Rosenberger G, Rost HL, Vichalkovski A, Gstaiger M, Aebersold R. 2013. Quantifying protein interaction dynamics by SWATH mass spectrometry: application to the 14-3-3 system. Nat Methods 10:1246–1253. doi:10.1038/nmeth.2703. 24162925

[82] Perez-Riverol Y, Csordas A, Bai J, Bernal-Llinares M, Hewapathirana S, Kundu DJ, Inuganti A, Griss J, Mayer G, Eisenacher M, Perez E, Uszkoreit J, Pfeuffer J, Sachsenberg T, Yilmaz S, Tiwary S, Cox J, Audain E, Walzer M, Jarnuczak AF, Ternent T, Brazma A, Vizcaino JA. 2019. The PRIDE database and related tools and resources in 2019: improving support for quantification data. Nucleic Acids Res 47:D442–D450. doi:10.1093/nar/gky1106. 30395289 PMC6323896

