# Supplementary material for: Spotted Fever Group Rickettsia Trigger Species-Specific Alterations in Macrophage Proteome Signatures with Different Impacts in Host Innate Inflammatory Responses
Source: Microbiol Spectr. 2021 Dec 22;9(3):e00814-21. doi: 10.1128/spectrum.00814-21 (PMC8693926; doi:10.1128/spectrum.00814-21)
Supplement: SUPPLEMENTAL FILE 1 — Supplemental material. Download SPECTRUM00814-21_Supp_1_seq13.pdf, PDF file, 5.4 MB [file spectrum00814-21_supp_1_seq13.pdf]

Supplemental Material

Supplementary Tables (Legends; Excel tables)

Table S1 - List of the 1,925 confidently quantified host proteins and the associated fold change upon infection of THP-1 macrophages with R. parkeri (R.p.), R. africae (R.a.), and R. massiliae (R.ma.).

Table S2 - List of the quantified host proteins and the associated fold change upon infection of THP-1 macrophages with R. parkeri (R.p.), R. africae (R.a.), and R. massiliae (R.ma.) that are categorized as: Neutrophil degranulation (GO:0043312) - Sheet2; Translation (GO:0006412) - Sheet3; mRNA splicing via spliceosome (GO:0000398) and mRNA export from nucleus (GO:0006406) - Sheet4; Regulation of cytoskeleton organization (GO:0032956) - Sheet5.

Table S3 - List of the 1,925 confidently quantified host proteins and the associated fold change between the following infection conditions: R. africae (R.a.)-infected THP-1 macrophages vs R. massiliae (R.ma.)-infected THP-1 macrophages - Sheet2; R. africae (R.a.)-infected THP-1 macrophages vs R. parkeri (R.p.)-infected THP-1 macrophages - Sheet3; R. massiliae (R.ma.)-infected THP-1 macrophages vs R. parkeri (R.p.)-infected THP-1 macrophages - Sheet4.

Table S4 - List of the quantified host proteins within specific annotation categories, and the associated fold change between the following infection conditions: R. africae (R.a.)-infected THP-1 macrophages vs. R. massiliae (R.ma.)-infected THP-1 macrophages - Sheet2; R. africae (R.a.)-infected THP-1 macrophages vs. R. parkeri (R.p.)-infected THP-1 macrophages - Sheet3; R.

massiliae (R.ma.)-infected THP-1 macrophages vs. R. parkeri (R.p.)-infected THP-1 macrophages - Sheet4.

Table S5 - List of the quantified host proteins and the associated fold change upon infection of THP-1 macrophages with R. parkeri (R.p.), R. africae (R.a.), and R. massiliae (R.ma.) that are categorized as: Innate immune responses (GO:0045087) - Sheet2; RIG-I-like receptor signaling pathway (hsa04622) - Sheet3.

Table S6 - List of the host proteins that were confidentially quantified in both datasets and its categorization according to GO or hsa terms. List of the 684 host proteins that were confidently quantified in all infection conditions and the associated fold change between infected and uninfected THP-1 macrophages. Proteins that were considered to significantly increase or decrease its abundance upon infection are colored in orange and blue, respectively. - Sheet2; Protein folding (GO:0006457) - Sheet3; TCA cycle (hsa00020) - Sheet4; Oxidative phosphorylation (hsa00190) - Sheet5;

Table S7 - Detailed information about SWATH windows.

Supplementary Figures

Figure S1

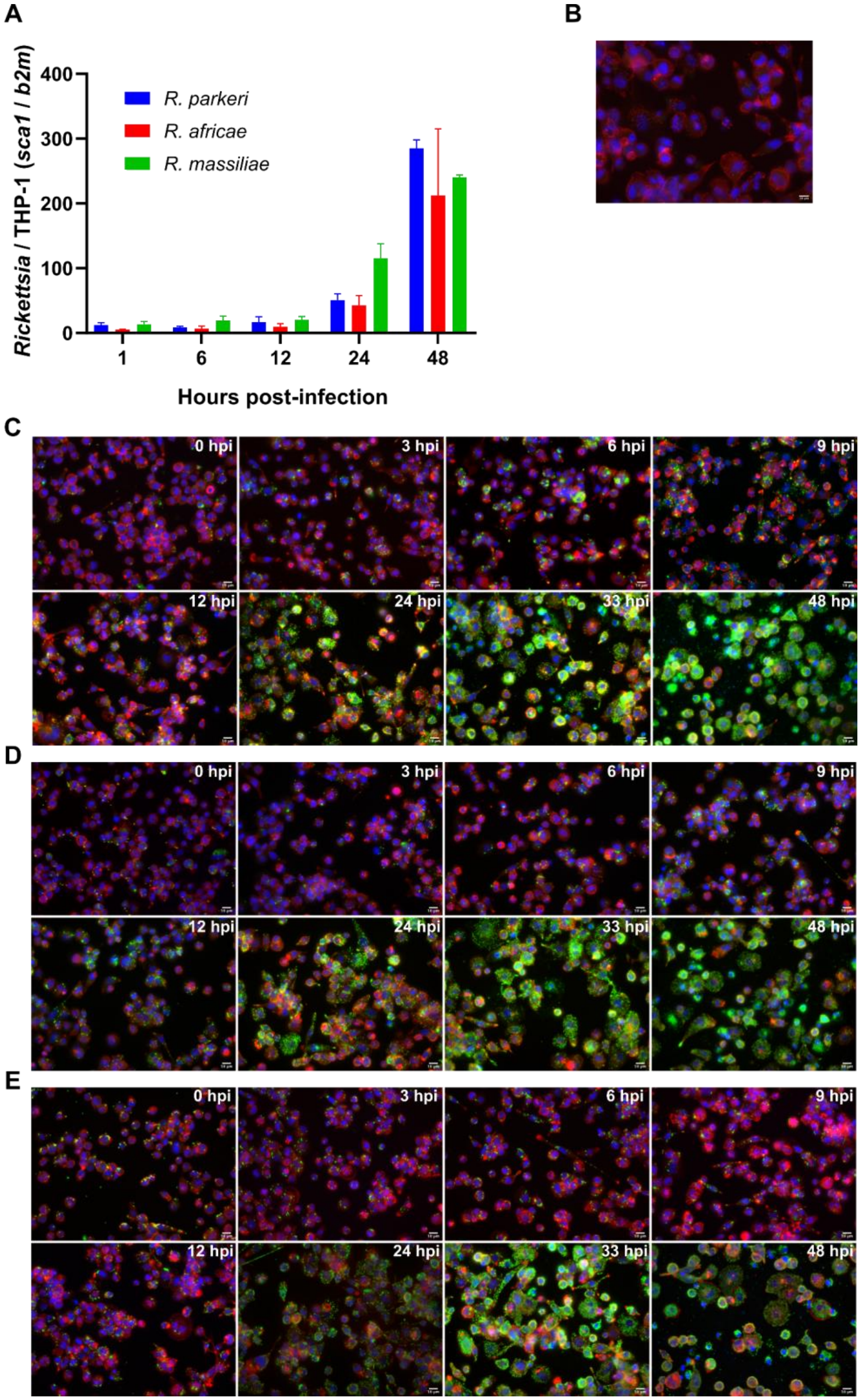

Fig S1. SFG *Rickettsia* species responsible for mild rickettsioses are able to proliferate within THP-1 macrophages. (**A**) PMA-differentiated THP-1 cells were infected with *R. parkeri*, *R. africae* and *R. massiliae* (MOI=10) and genomic DNA was extracted at 1 hour, 6,12, 24, and 48 hours post-infection. Quantitative PCR data are expressed as the ratio of *Rickettsia sca1* vs. *b2m* DNA content. (**B-E**) Immunofluorescence microscopy of THP-1 macrophages uninfected (**B**), and infected with *R. parkeri* (**C**), *R. africae* (**D**), and *R. massiliae* (**E**) (MOI=10) at several time points post-infection from left to right and top to bottom (0, 3, 6, 9, 12, 24, 33 and 48 hours post-infection. Cells were stained with DAPI(blue) to stain host nuclei, Phalloidin (red) to stain actin, and rabbit anti-*Rickettsia* polyclonal antibody NIH/RML I7198 followed by Alexa Fluor 488 (green) to stain *Rickettsia*. Scale bar = 10 µm.

Figure S2

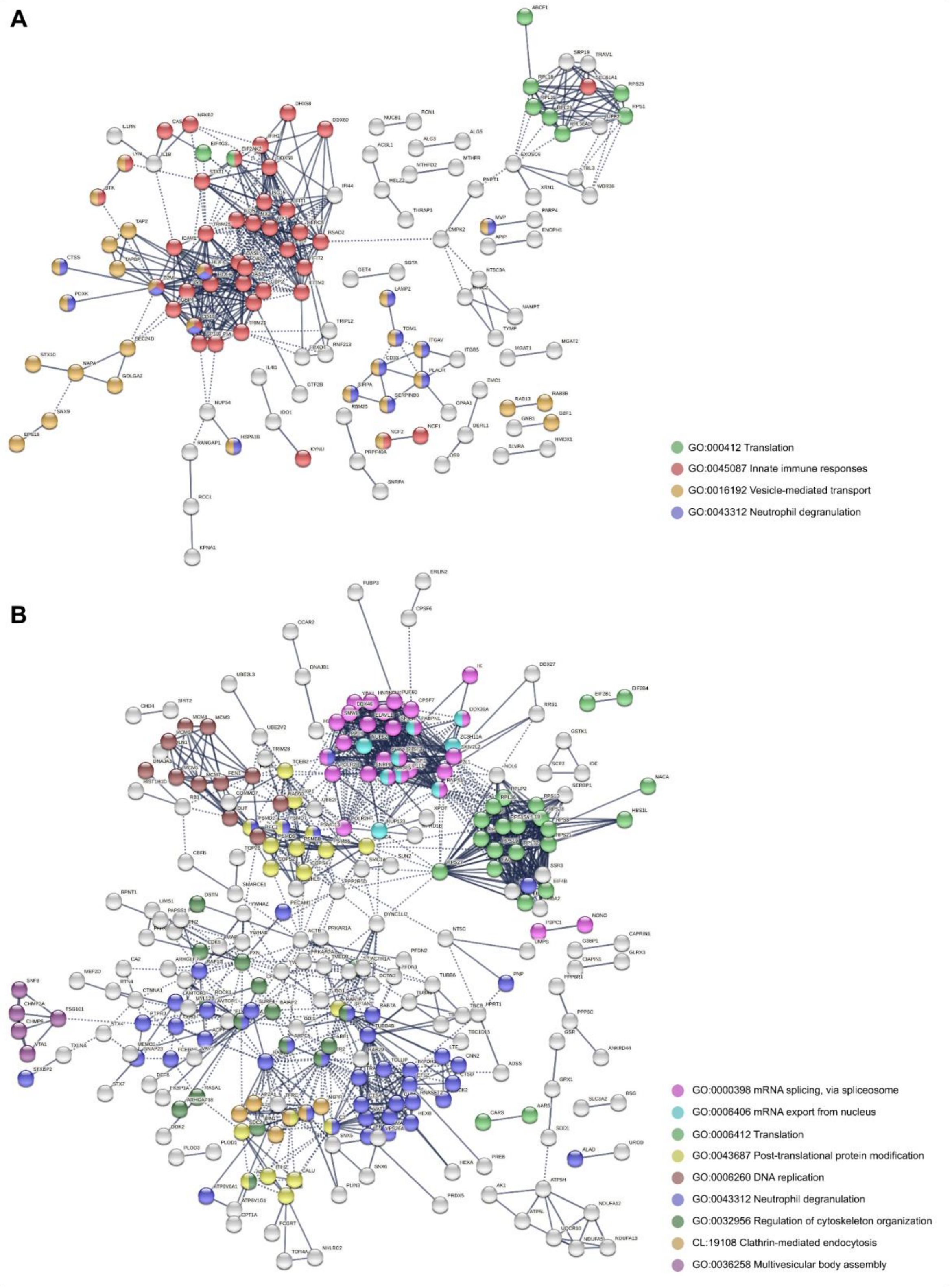
A
GO:000412 Translation
GO:0045087 Innate immune responses
GO:0016192 Vesicle-mediated transport
GO:0043312 Neutrophil degranulation
B
GO:0000398 mRNA splicing, via spliceosome
GO:0006406 mRNA export from nucleus
GO:0006412 Translation
GO:0043687 Post-translational protein modification
GO:0006260 DNA replication
GO:0043312 Neutrophil degranulation
GO:0032956 Regulation of cytoskeleton organization
CL:19108 Clathrin-mediated endocytosis
GO:0036258 Multivesicular body assembly

Fig S2. Clustering of host proteins with altered abundance upon infection with *R. parkeri*. (A and B) Protein-protein interaction network for the 221 and 411 host proteins with increased **(A)** and decreased **(B)** abundance upon infection with *R. parkeri*, respectively. The analysis was carried out with STRING 11.0 (http://string-db.org/) using the highest confidence (0.9) score and MCL clustering with an inflation parameter of 3. Nodes are represented with different colors according to their categorization in gene ontology (GO) or local network cluster, STRING (CL) terms.

Figure S3

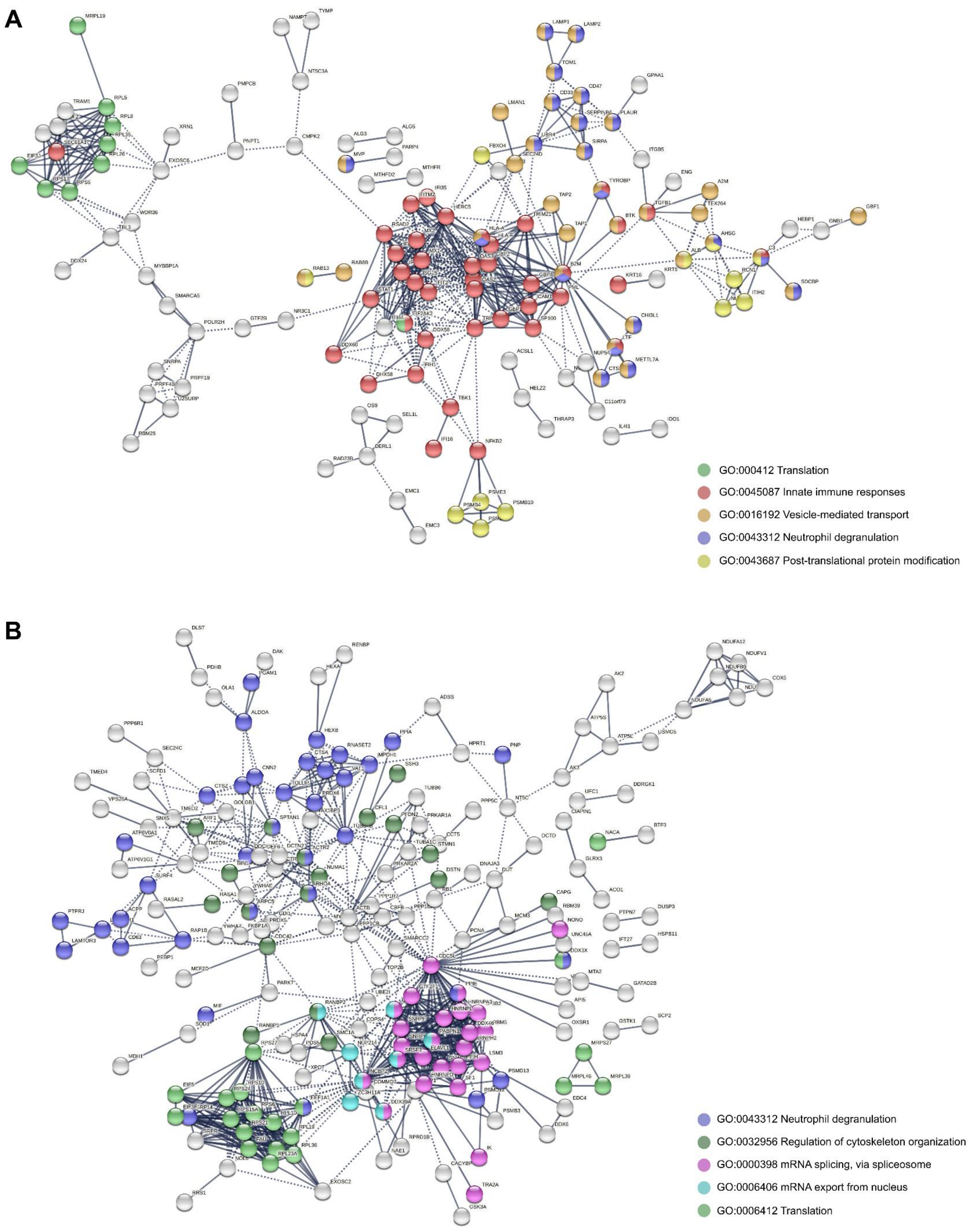
A
GO:000412 Translation
GO:0045087 Innate immune responses
GO:0016192 Vesicle-mediated transport
GO:0043312 Neutrophil degranulation
GO:0043687 Post-translational protein modification
B
GO:0043312 Neutrophil degranulation
GO:0032956 Regulation of cytoskeleton organization
GO:0000398 mRNA splicing, via spliceosome
GO:0006406 mRNA export from nucleus
GO:0006412 Translation

Fig S3. Clustering of host proteins with altered abundance upon infection with *R. africae*. (A and B) Protein-protein interaction network for the 250 and 309 host proteins with increased **(A)** and decreased **(B)** abundance upon infection with *R. africae*, respectively. The analysis was carried out with STRING 11.0 (http://string-db.org/) using the highest confidence (0.9) score and MCL clustering with an inflation parameter of 3. Nodes are represented with different colors according to their categorization in gene ontology (GO) terms.

Figure S4

A

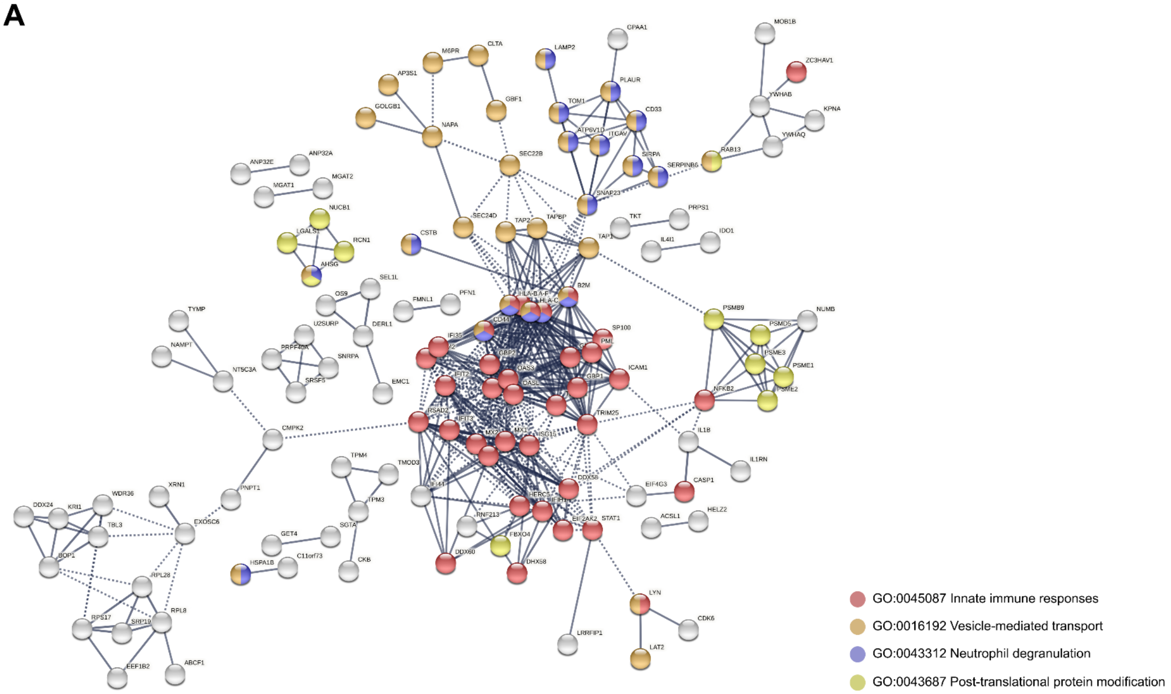

GO:0045087 Innate immune responses
GO:0016192 Vesicle-mediated transport
GO:0043312 Neutrophil degranulation
GO:0043687 Post-translational protein modification

B

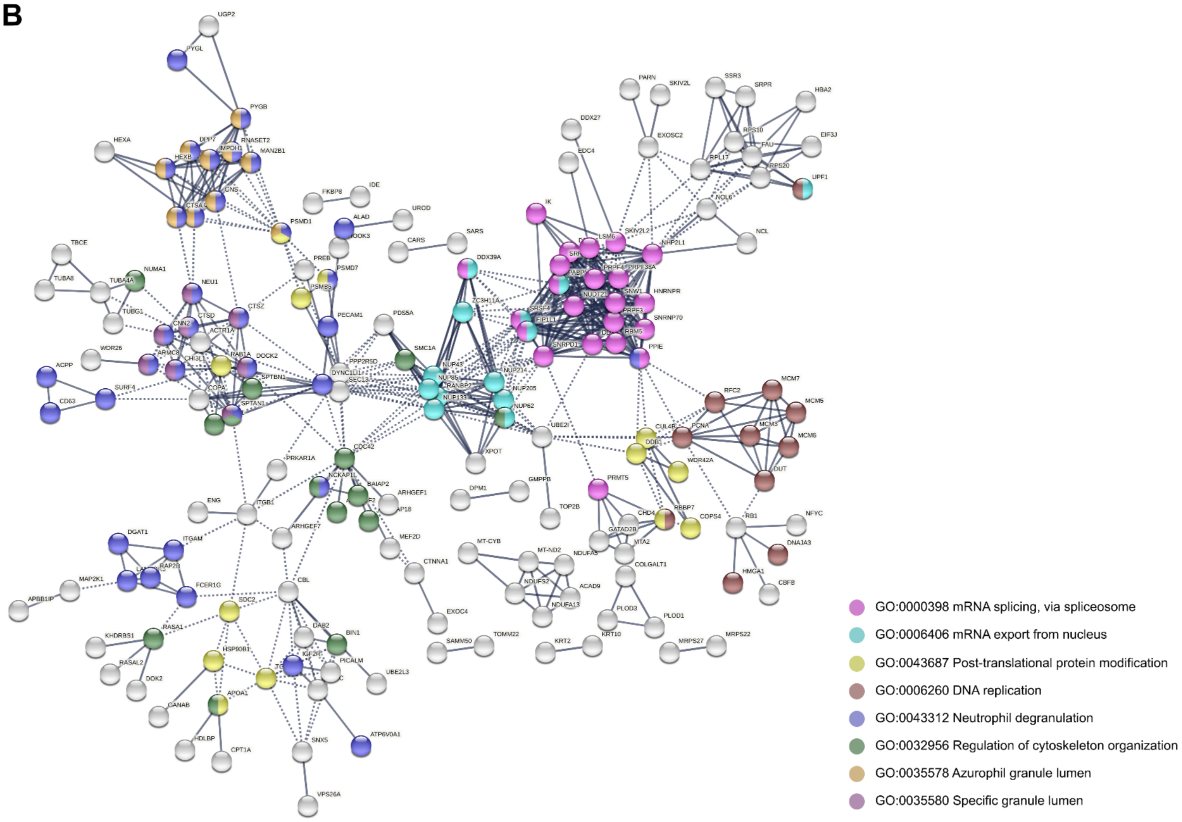

GO:0000398 mRNA splicing, via spliceosome
GO:0006406 mRNA export from nucleus
GO:0043687 Post-translational protein modification
GO:0006260 DNA replication
GO:0043312 Neutrophil degranulation
GO:0032956 Regulation of cytoskeleton organization
GO:0035578 Azurophil granule lumen
GO:0035580 Specific granule lumen

Fig S4. Clustering of host proteins with altered abundance upon infection with *R. massiliae*. **(A and B)** Protein-protein interaction network for the 238 and 307 host proteins with increased **(A)** and decreased **(B)** abundance upon infection with *R. massiliae*, respectively. The analysis was carried out with STRING 11.0 (http://string-db.org/) using the highest confidence (0.9) score and MCL clustering with an inflation parameter of 3. Nodes are represented with different colors according to their categorization in gene ontology (GO) terms.

Supplementary File 1 (Figures S5-S20) - Compilation of Western blotting exposures and total loading controls for representative images shown in Figure 7.

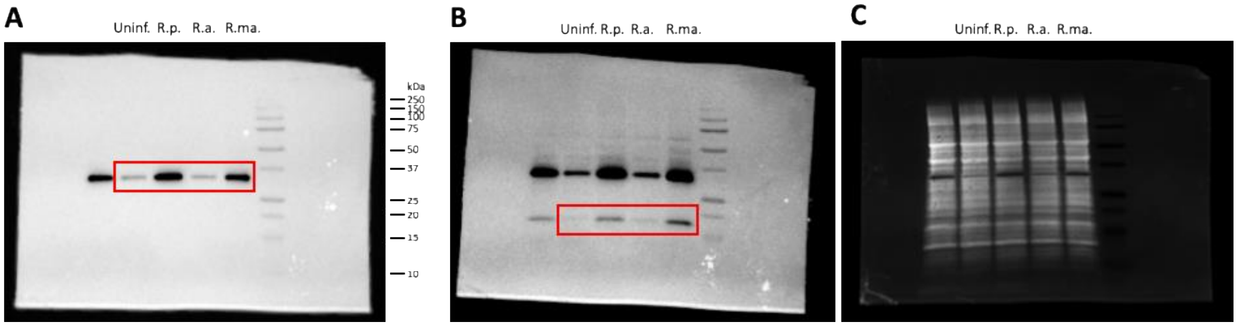

Fig S5. Western blotting exposures from data relative to Figure 9 - panel A. (A-C) Total protein extracts from uninfected THP-1 macrophages (uninf.), *R. parkeri*- (R.p.)-, *R. africae*- (R.a.), and *R. massiliae*- (R.ma.) infected THP-1 macrophages were probed for interleukin-1β precursor **(A)** and activated forms **(B)**. Immunoblot analysis with SERVA purple was used as the protein loading control **(C)**. Red box represent the cropped regions selected for representation in main Figure.

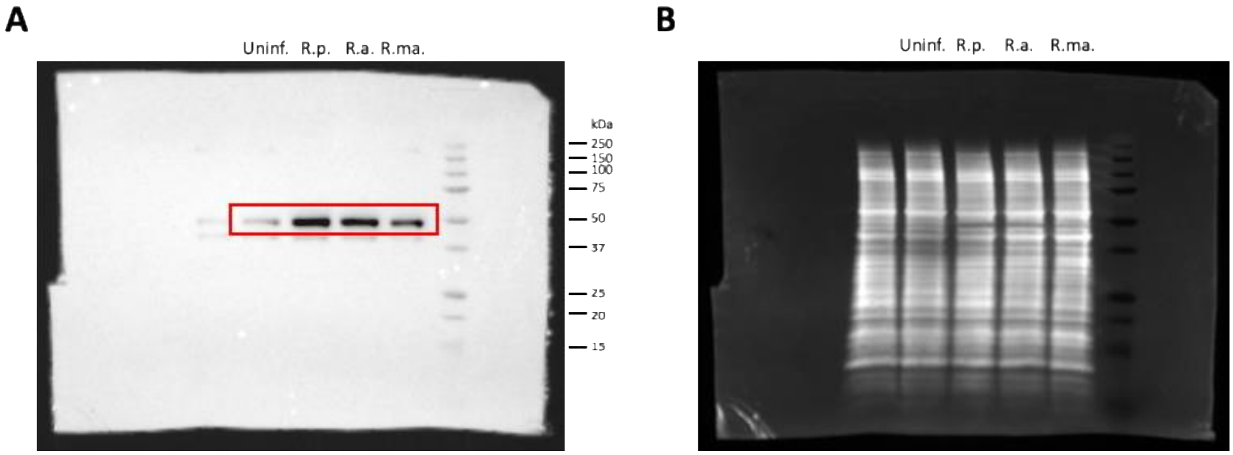

Fig S6. Western blotting exposures from data relative to Figure 9 - panel B. (A-B) Total protein extracts from uninfected THP-1 macrophages (uninf.), *R. parkeri*- (R.p.)-, *R. africae*- (R.a.), and *R. massiliae*- (R.ma.) infected THP-1 macrophages were probed for caspase-1 **(A)**.

Immunoblot analysis with SERVA purple was used as the protein loading control **(B)**. Red box represent the cropped regions selected for representation in main Figure.

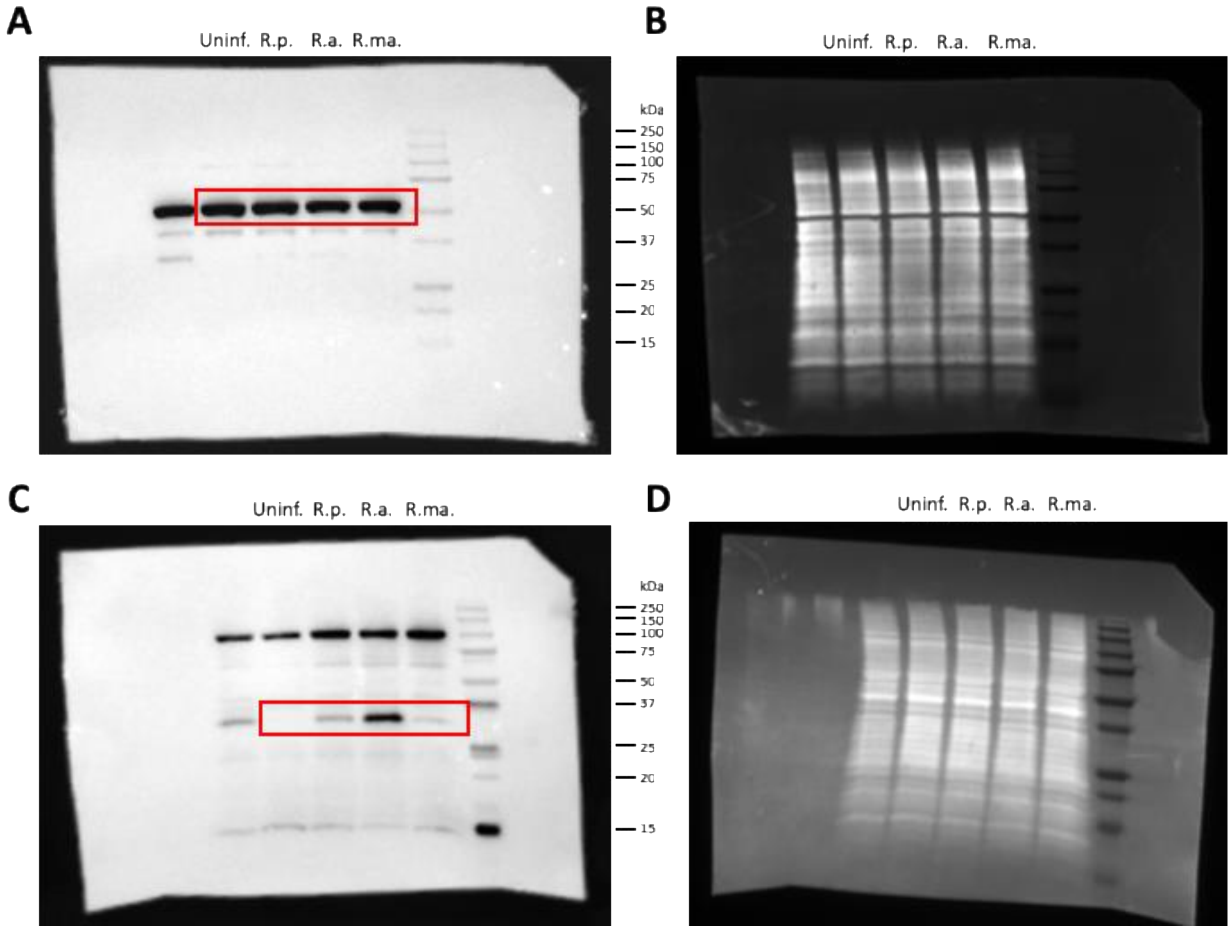

Fig S7. Western blotting exposures from data relative to Figure 9 - panel C. (A-D) Total protein extracts from uninfected THP-1 macrophages (uninf.), *R. parkeri*- (R.p.)-, *R. africae*- (R.a.), and *R. massiliae*- (R.ma.) infected THP-1 macrophages were probed for Gasdermin D precursor **(A)** and activated forms **(C)**. Immunoblot analysis with SERVA purple was used as the protein loading control **(B and D)**. Red box represent the cropped regions selected for representation in main Figure.

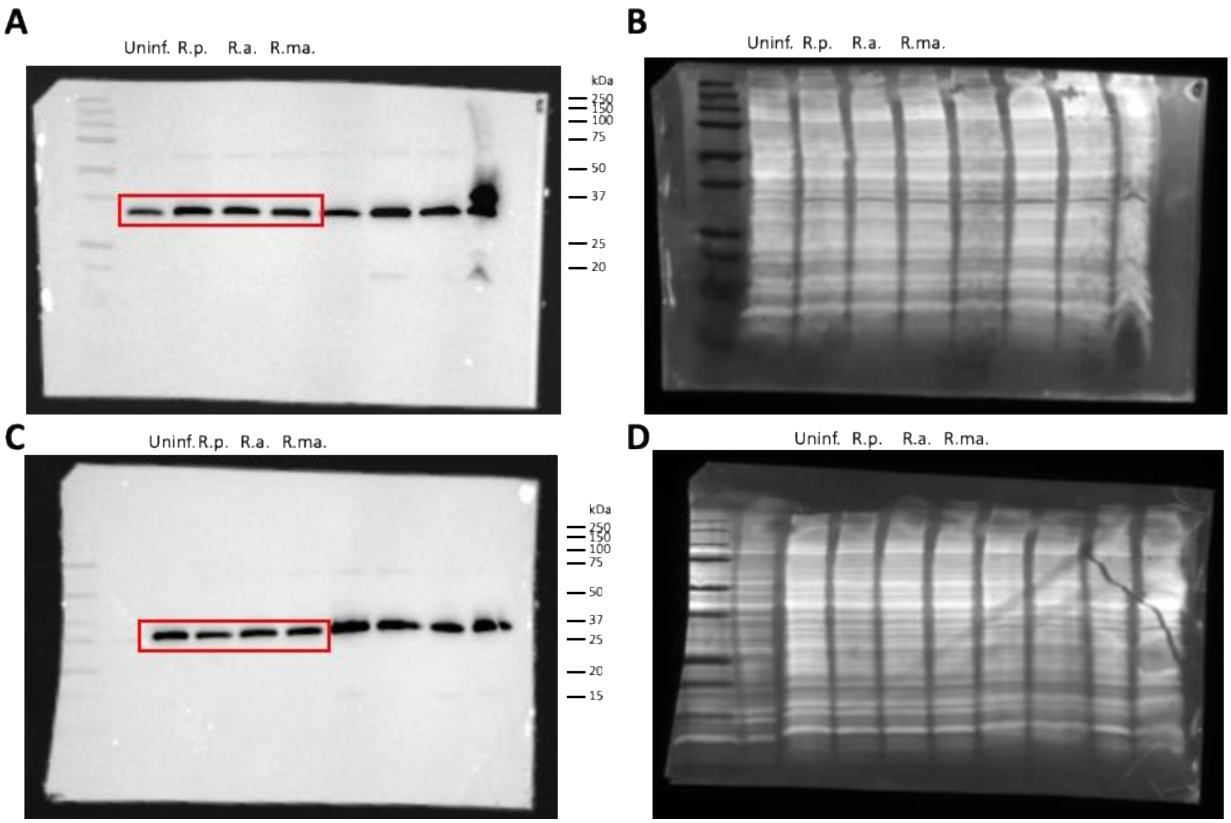

Fig S8. Western blotting exposures from data relative to Figure 9 - panel D. (A-D) Total protein extracts from uninfected THP-1 macrophages (uninf.), *R. parkeri*- (R.p.)-, *R. africae*- (R.a.), and *R. massiliae*- (R.ma.) infected THP-1 macrophages at 0 **(A)** and 3 **(C)** hpi were probed for interleukin-1β precursor form. Immunoblot analysis with SERVA purple was used as the protein loading control for samples at 0 **(B)** and 3 **(D)** hpi. Red box represent the cropped regions selected for representation in main Figure.

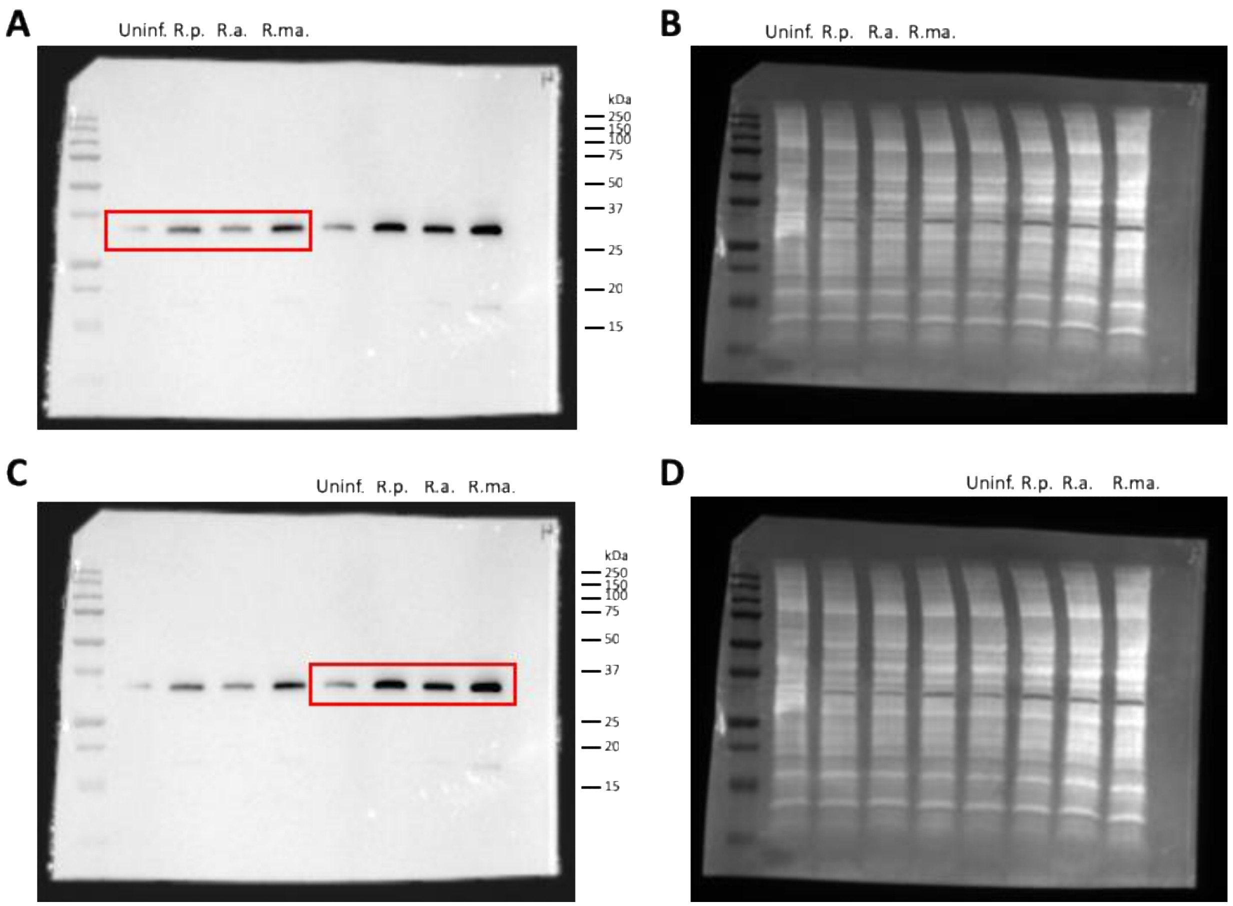

Fig S9. Western blotting exposures from data relative to Figure 9 - panel D. (A-D) Total protein extracts from uninfected THP-1 macrophages (uninf.), *R. parkeri*- (R.p.)-, *R. africae*- (R.a.), and *R. massiliae*- (R.ma.) infected THP-1 macrophages at 6 **(A)** and 9 **(C)** hpi were probed for interleukin-1β precursor form. Immunoblot analysis with SERVA purple was used as the protein loading control for samples at 6 **(B)** and 9 **(D)** hpi. Red box represent the cropped regions selected for representation in main Figure.

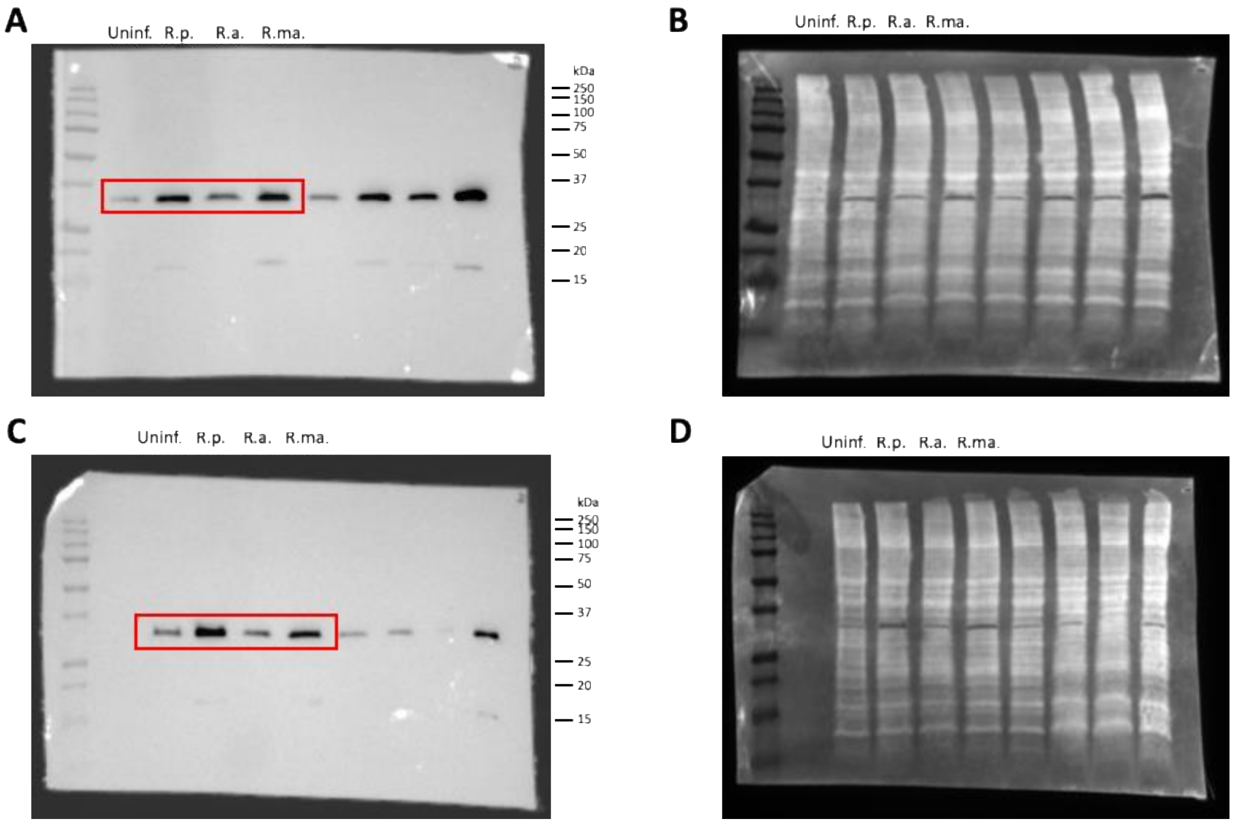

Fig S10. Western blotting exposures from data relative to Figure 9 - panel D. (A-D) Total protein extracts from uninfected THP-1 macrophages (uninf.), *R. parkeri*- (R.p.)-, *R. africae*- (R.a.), and *R. massiliae*- (R.ma.) infected THP-1 macrophages at 12 **(A)** and 33 **(C)** hpi were probed for interleukin-1β precursor form. Immunoblot analysis with SERVA purple was used as the protein loading control for samples at 12 **(B)** and 33 **(D)** hpi. Red box represent the cropped regions selected for representation in main Figure.

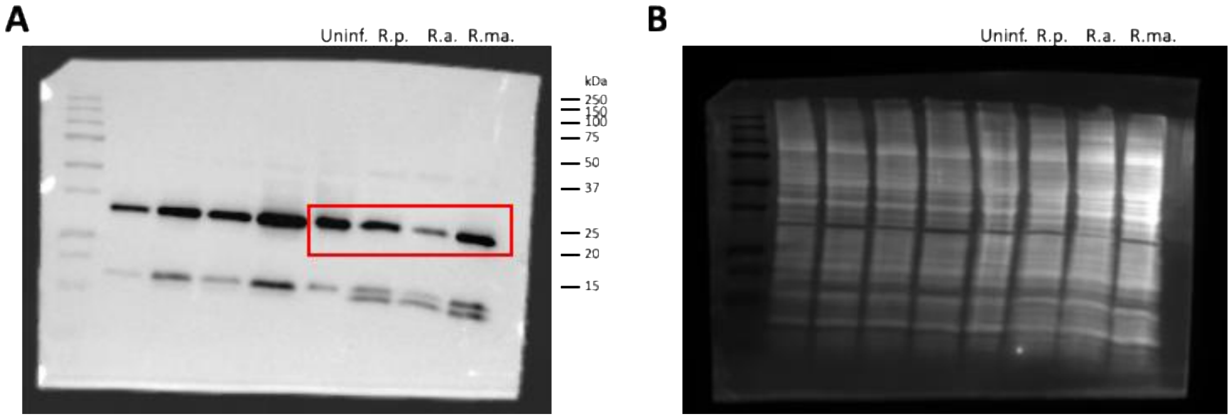

Fig S11. Western blotting exposures from data relative to Figure 9 - panel D. (A-B) Total protein extracts from uninfected THP-1 macrophages (uninf.), *R. parkeri*- (R.p.)-, *R. africae*- (R.a.), and *R. massiliae*- (R.ma.) infected THP-1 macrophages at 48 hpi were probed for interleukin-1β precursor form **(A)**. Immunoblot analysis with SERVA purple was used as the protein loading control **(B)**. Red box represent the cropped regions selected for representation in main Figure.

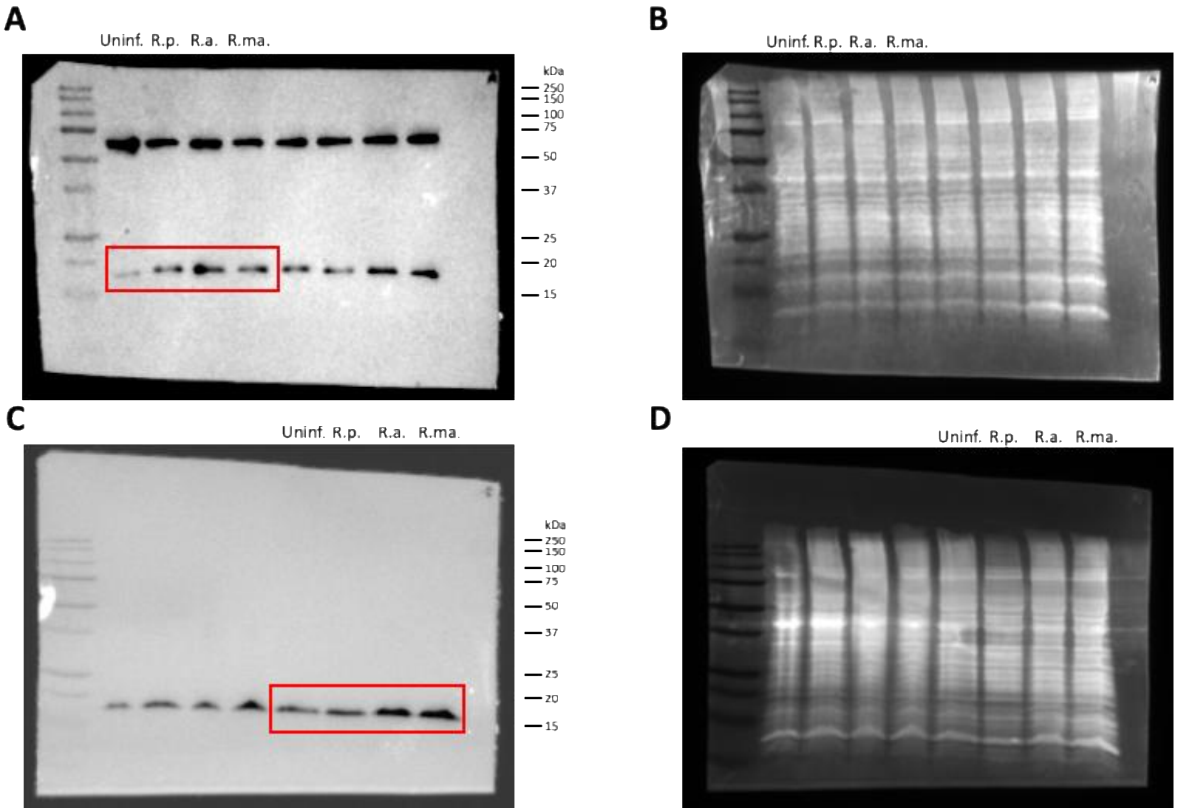

Fig S12. Western blotting exposures from data relative to Figure 9 - panel E. (A-D) Total protein extracts from uninfected THP-1 macrophages (uninf.), *R. parkeri*- (R.p.)-, *R. africae*- (R.a.), and *R. massiliae*- (R.ma.) infected THP-1 macrophages at 0 **(A)** and 3 **(C)** hpi were probed for interleukin-1β activated form. Immunoblot analysis with SERVA purple was used as the protein loading control for samples at 0 **(B)** and 3 **(D)** hpi. Red box represent the cropped regions selected for representation in main Figure.

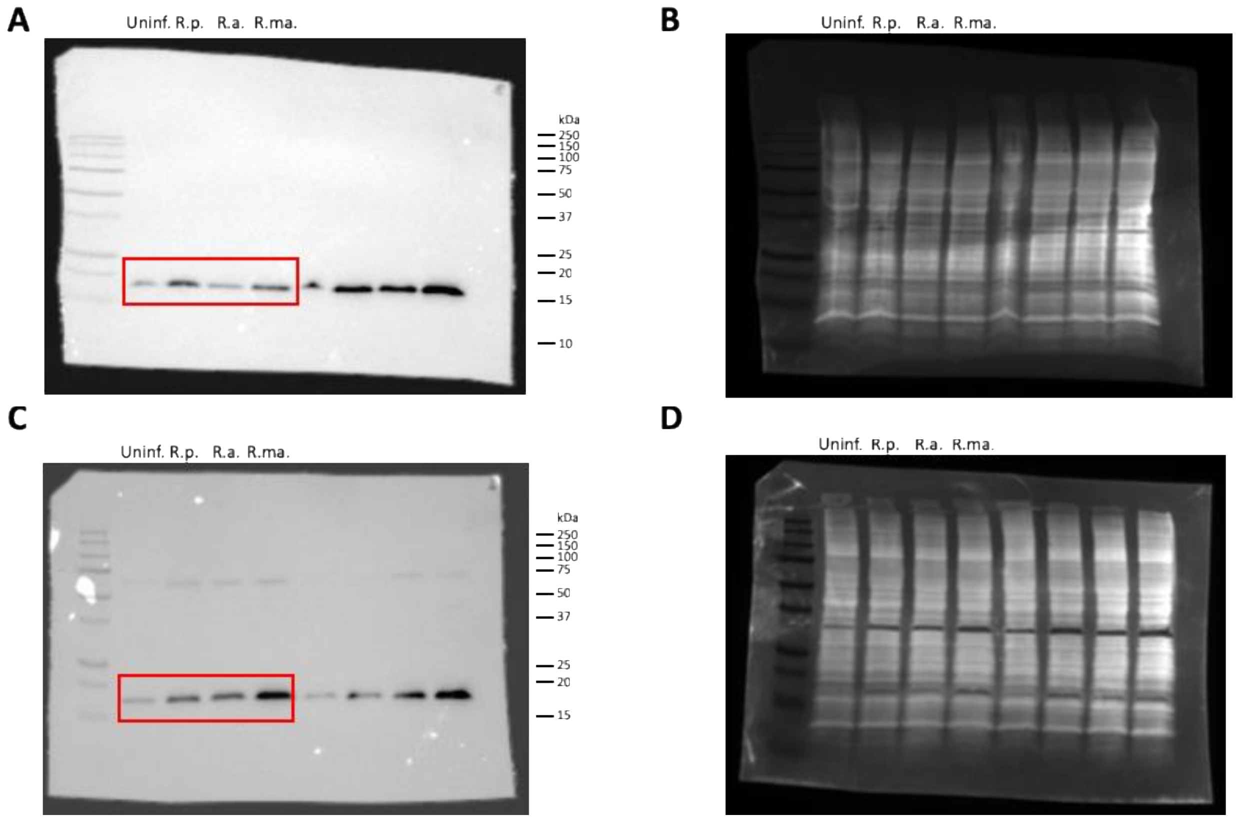

Fig S13. Western blotting exposures from data relative to Figure 9 - panel E. (A-D) Total protein extracts from uninfected THP-1 macrophages (uninf.), *R. parkeri*- (R.p.)-, *R. africae*- (R.a.), and *R. massiliae*- (R.ma.) infected THP-1 macrophages at 6 **(A)** and 9 **(C)** hpi were probed for interleukin-1β activated form. Immunoblot analysis with SERVA purple was used as the protein loading control for samples at 6 **(B)** and 9 **(D)** hpi. Red box represent the cropped regions selected for representation in main Figure.

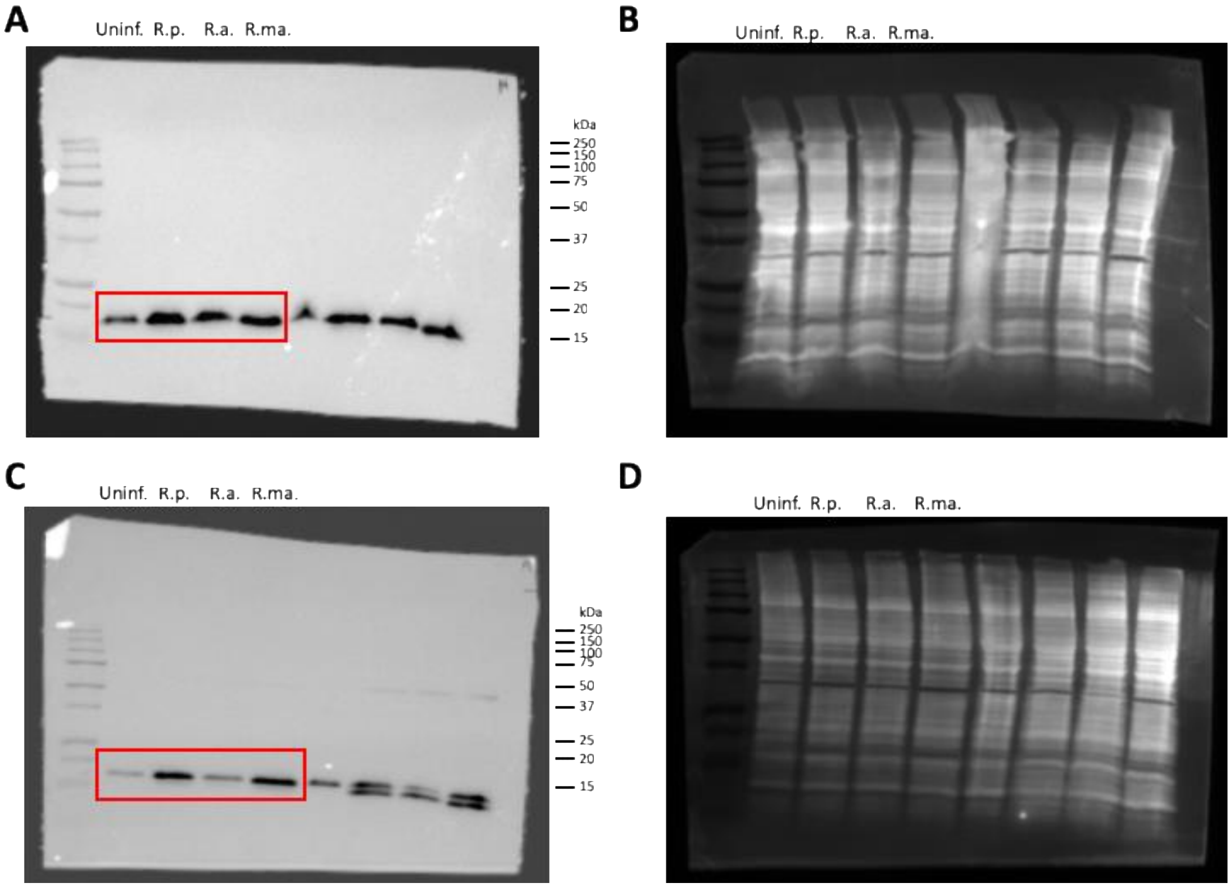

Fig S14. Western blotting exposures from data relative to Figure 9 - panel E. (A-D) Total protein extracts from uninfected THP-1 macrophages (uninf.), *R. parkeri*- (R.p.)-, *R. africae*- (R.a.), and *R. massiliae*- (R.ma.) infected THP-1 macrophages at 12 **(A)** and 33 **(C)** hpi were probed for interleukin-1β activated form. Immunoblot analysis with SERVA purple was used as the protein loading control for samples at 12 **(B)** and 33 **(D)** hpi. Red box represent the cropped regions selected for representation in main Figure.

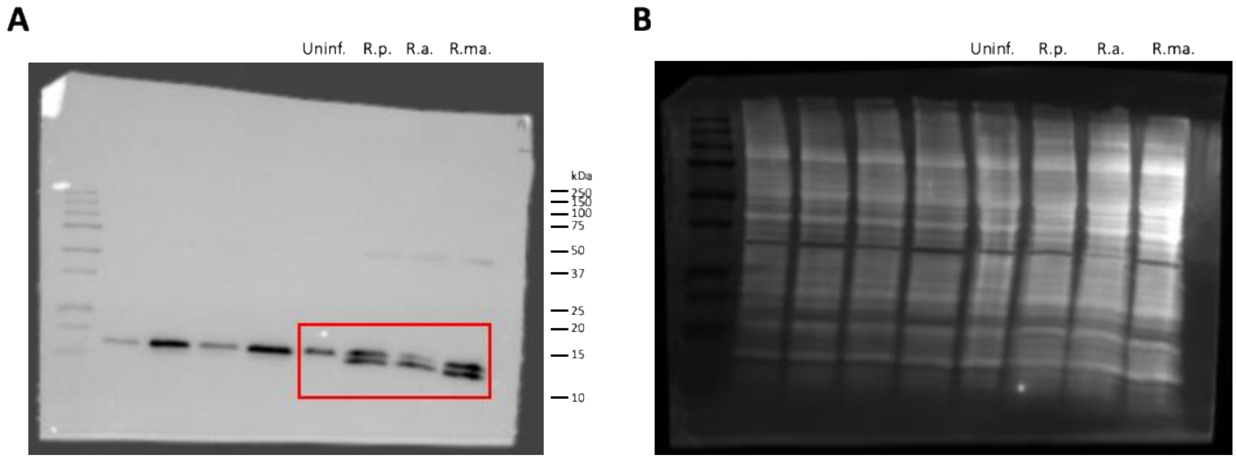

Fig S15. Western blotting exposures from data relative to Figure 9 - panel E. (A-B) Total protein extracts from uninfected THP-1 macrophages (uninf.), *R. parkeri*- (R.p.)-, *R. africae*- (R.a.), and *R. massiliae*- (R.ma.) infected THP-1 macrophages at 48 hpi were probed for interleukin-1β activated form **(A)**. Immunoblot analysis with SERVA purple was used as the protein loading control **(B)**. Red box represent the cropped regions selected for representation in main Figure.

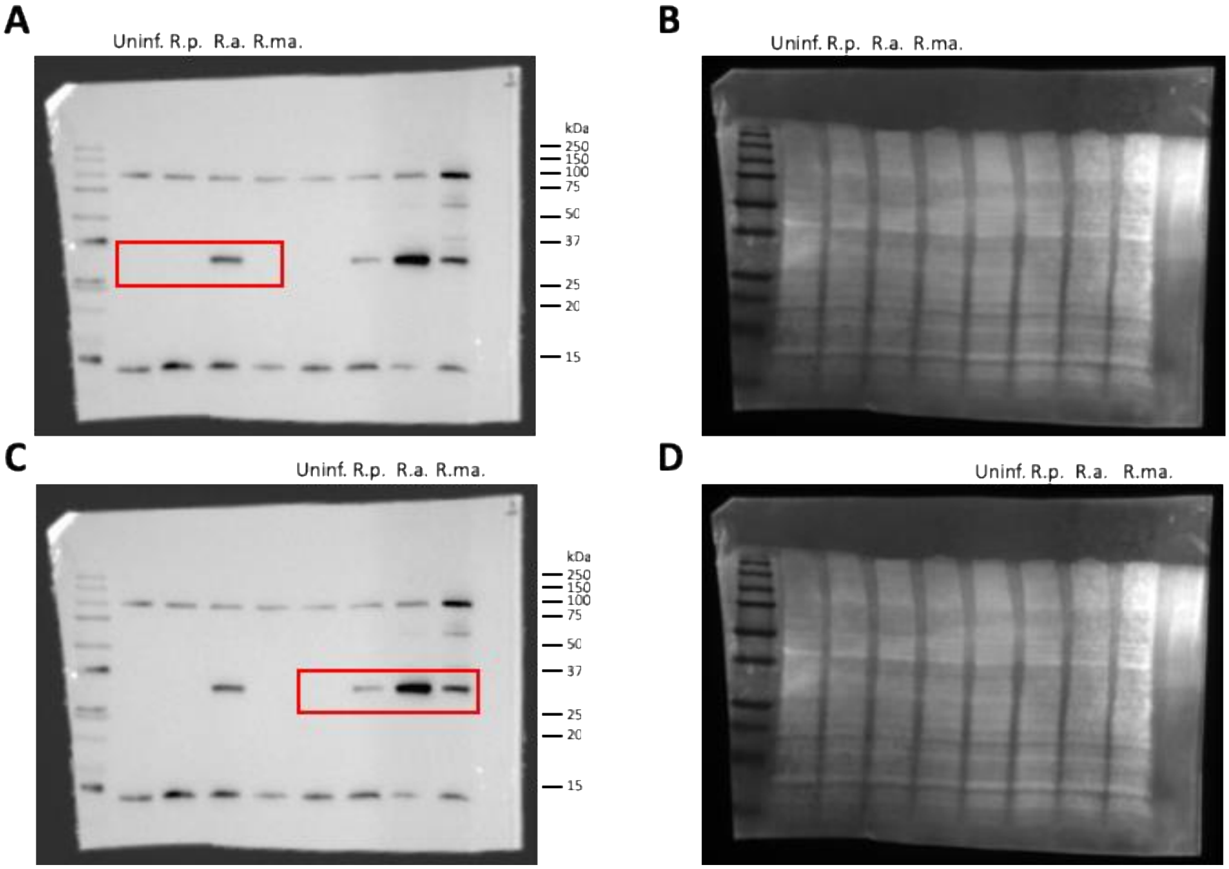

Fig S16. Western blotting exposures from data relative to Figure 9 - panel F. (A-D) Total protein extracts from uninfected THP-1 macrophages (uninf.), *R. parkeri*- (R.p.)-, *R. africae*- (R.a.), and *R. massiliae*- (R.ma.) infected THP-1 macrophages at 12 **(A)** and 24 **(C)** hpi were probed for Gasdermin D activated form. Immunoblot analysis with SERVA purple was used as the protein loading control for samples at 12 **(B)** and 24 **(D)** hpi. Red box represent the cropped regions selected for representation in main Figure.

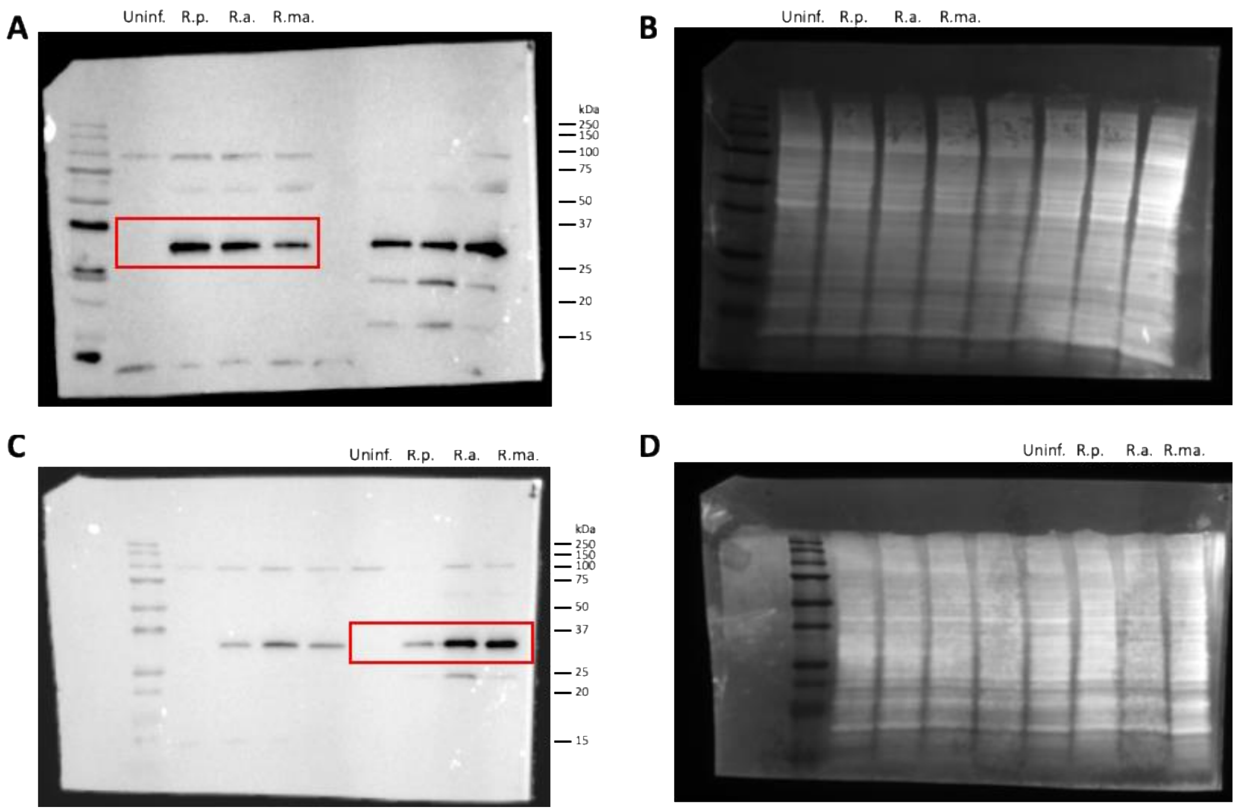

Fig S17. Western blotting exposures from data relative to Figure 9 - panel F. (A-D) Total protein extracts from uninfected THP-1 macrophages (uninf.), *R. parkeri*- (R.p.)-, *R. africae*- (R.a.), and *R. massiliae*- (R.ma.) infected THP-1 macrophages at 33 **(A)** and 48 **(C)** hpi were probed for Gasdermin D activated form. Immunoblot analysis with SERVA purple was used as the protein loading control for samples at 33 **(B)** and 48 **(D)** hpi. Red box represent the cropped regions selected for representation in main Figure.

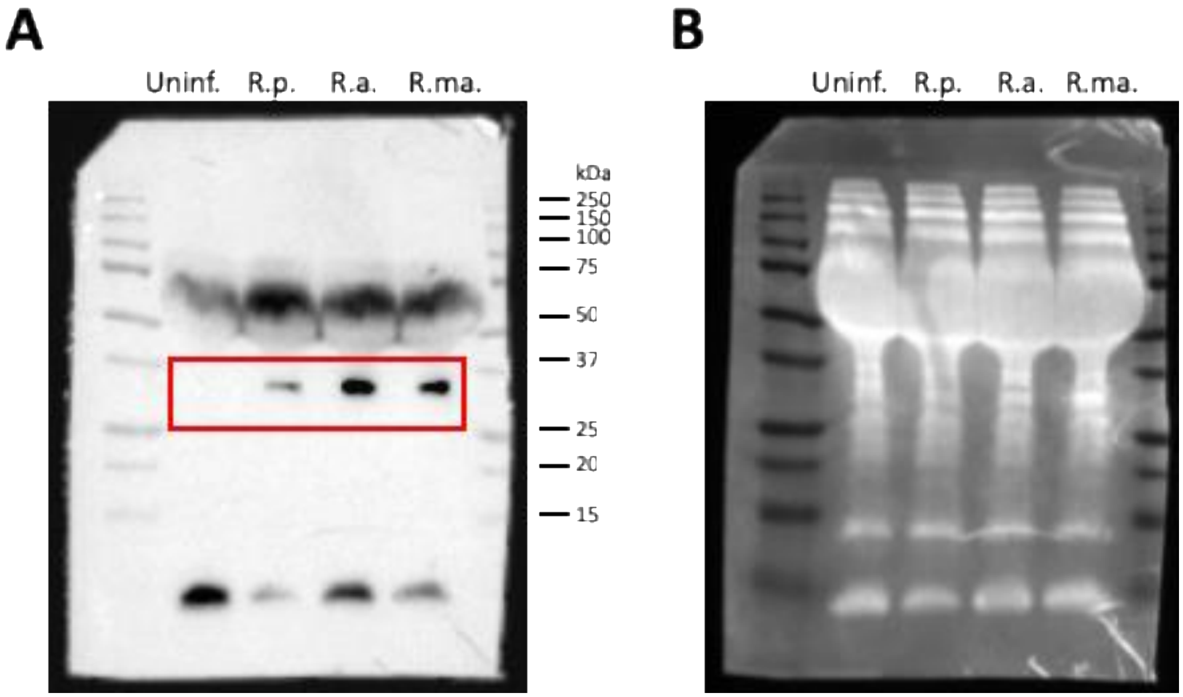

Fig S18. Western blotting exposures from data relative to Figure 9 - panel G. (A-B) Culture supernatants from uninfected THP-1 macrophages (uninf.), *R. parkeri*- (R.p.)-, *R. africae*- (R.a.), and *R. massiliae*- (R.ma.) infected THP-1 macrophages at 48 hpi were probed for HMGB1. Immunoblot analysis with SERVA purple was used as the protein loading control. Red box represent the cropped regions selected for representation in main Figure.

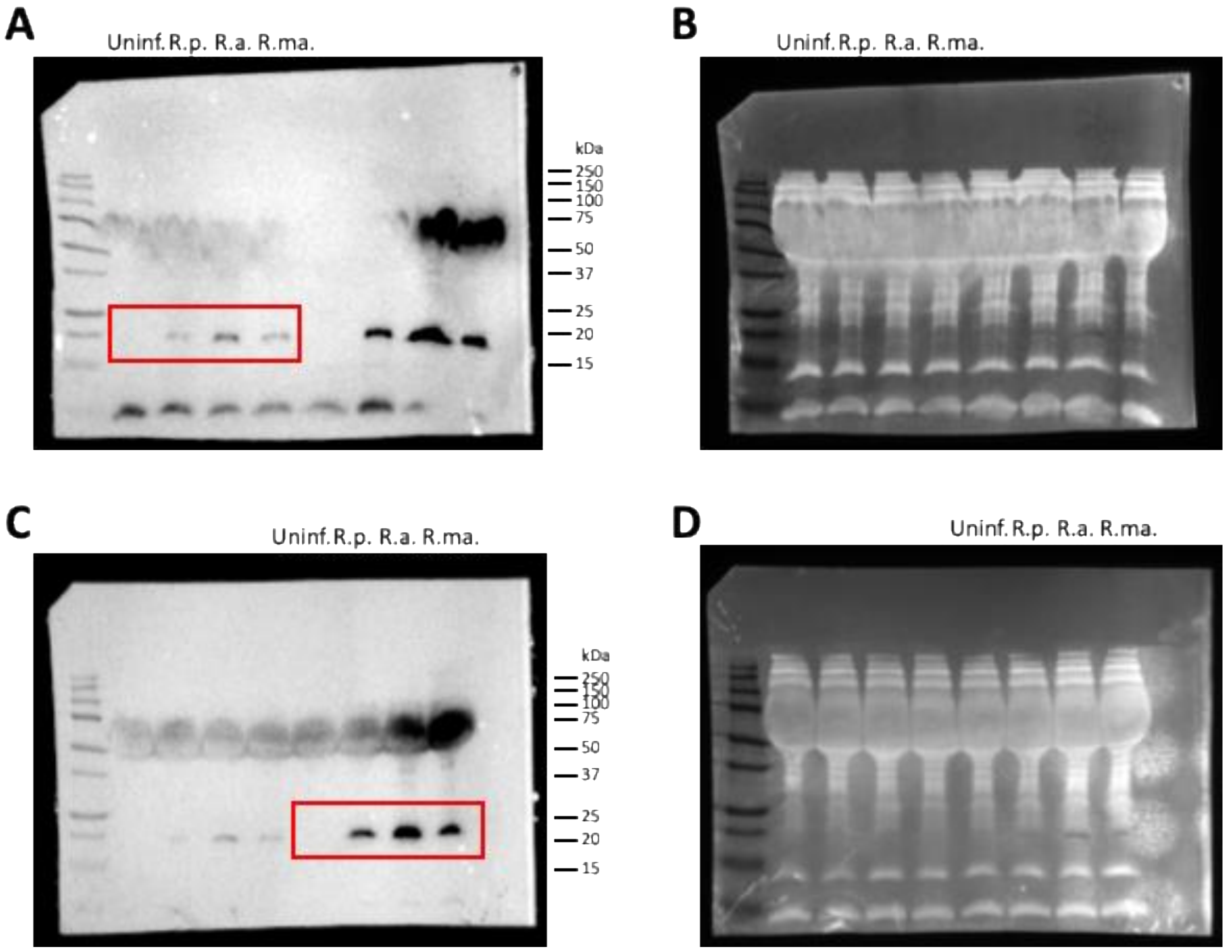

Fig S19. Western blotting exposures from data relative to Figure 9 - panel H. (A-D) Culture supernatants from uninfected THP-1 macrophages (uninf.), *R. parkeri*- (R.p.)-, *R. africae*- (R.a.), and *R. massiliae*- (R.ma.) infected THP-1 macrophages at 12 **(A)** and 24 **(C)** hpi were probed for interleukin-1β activated form. Immunoblot analysis with SERVA purple was used as the protein loading control for samples at 12 **(B)** and 24 **(D)** hpi. Red box represent the cropped regions selected for representation in main Figure.

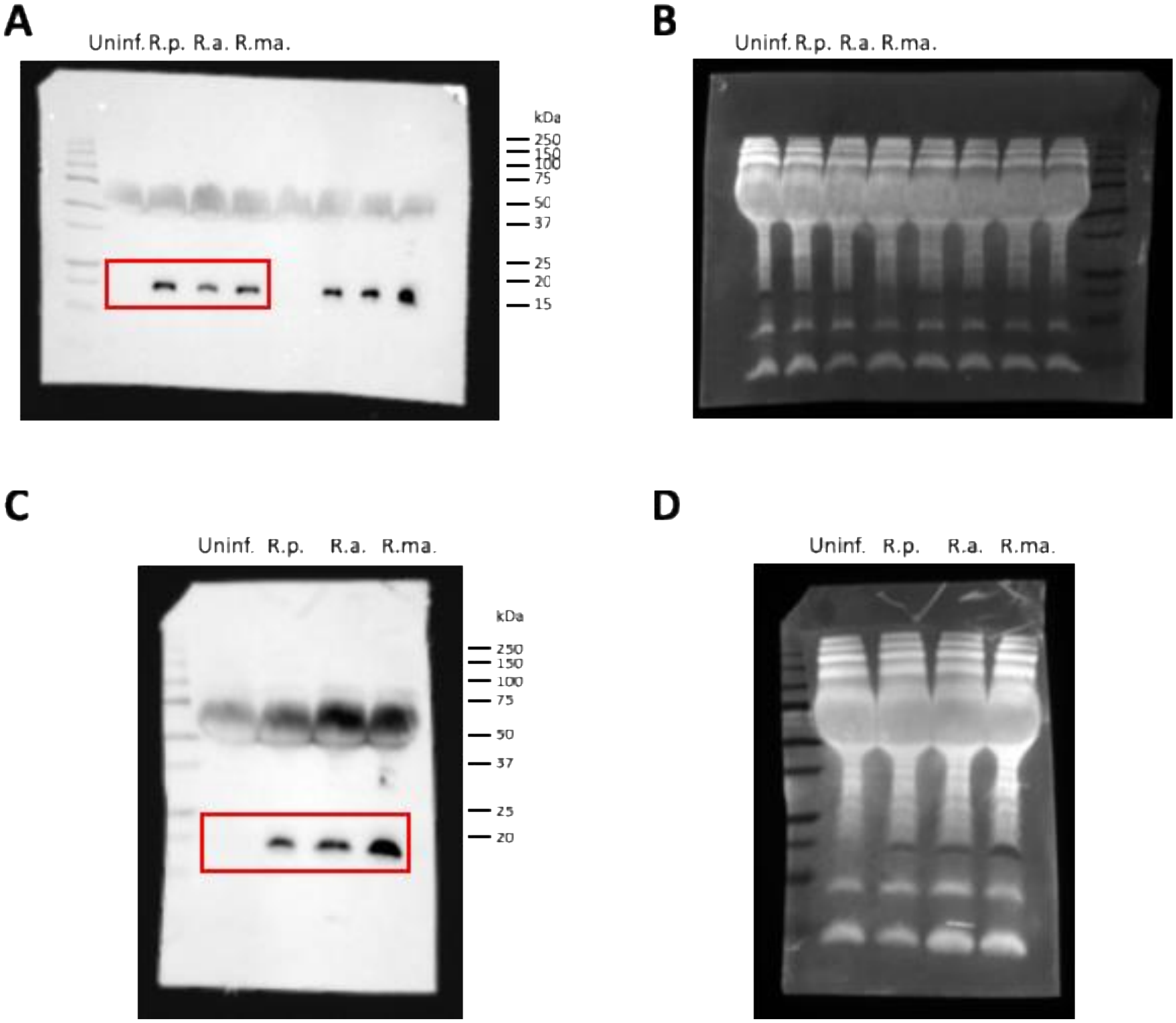

Fig S20. Western blotting exposures from data relative to Figure 9 - panel H. (A-D) Culture supernatants from uninfected THP-1 macrophages (uninf.), *R. parkeri*- (R.p.)-, *R. africae*- (R.a.), and *R. massiliae*- (R.ma.) infected THP-1 macrophages at 33 **(A)** and 48 **(C)** hpi were probed for interleukin-1β activated form. Immunoblot analysis with SERVA purple was used as the protein loading control for samples at 33 **(B)** and 48 **(D)** hpi. Red box represent the cropped regions selected for representation in main Figure.

Supplementary File 2 (Figures S21-S26) - Comparison of extracted ion chromatograms (XIC) for selected peptides obtained in the present dataset with the mass spectrometry proteomics dataset obtained for *R. conorii* infected cells. In THP-1 macrophages infected with the highly pathogenic *R. conorii*, we did not observe a drastic increase in interferon-stimulated responses (1). Those proteins were either not identified in the samples or not quantified with the quality to be reported. Using the SWATH files acquired at that time and extracting the information with the library of identified proteins in the present dataset, we re-mined the datasets to search for the peptides of 4 proteins of interest: IL-1β, Mx1 (P20591), RIG-I, and ISG15. In Figures S1-S4 (presented below), we report the extracted ion chromatogram (XIC) for a given peptide from the selected proteins in 4 different samples: Uninfected THP-1 sample (control) and *R. conorii*-infected sample from the former dataset; Uninfected THP-1 sample (control) and *R. massiliae*-infected sample from the present dataset. The same approach was used for a peptide of the internal standard (malE-GFP) (Fig. S5), and the protein myosin 9 that has no regulation reported in the present dataset but is consistently quantified throughout datasets (Fig. S6). These two proteins serve as positive controls for our comparative approach. As the chromatographic conditions used for the samples in the current batch and the ones in the *R. conorii* batch are not the same, the alignment of the retention times (although effective) made it necessary to use wider extraction windows in the *R. conorii* batch samples.

From the four proteins analyzed, only IL-1β (Fig. S21) was undoubtedly present in *R. conorii*-infected THP-1 with high intensity, whereas in the respective control the peptide could not be found. These results recapitulate the same regulation observed in the present study, represented by the uninfected control and *R. massiliae*-infected sample.

For Mx1 and RIG-I (Fig. S22, S23), the peptide was not found in *R. conorii*, or any control sample (from this or the previous batch). However, the peak intensity is evident in *R. massiliae*-infected sample denoting the overrepresentation of this protein upon infection with mildly pathogenic rickettsiae.

For ISG15 (Fig. S24), the peptide is visible in *R. massiliae*-infected THP-1 and its control sample, with a visible overrepresentation in *R. massiliae*. In the case of the *R. conorii* batch, there is a visible peak (in the far end of the extraction window) with very low intensity in both *R. conorii* and its control, showing that the protein may be present but is not regulated in the infection condition.

The control peptides for malE-GFP and myosin 9 (Fig. S25, S26) are present in all studied samples, confirming that these proteins are present in all samples and demonstrating the effectiveness of this approach.

Interleukin- 1beta (SLVMSGPYELK)

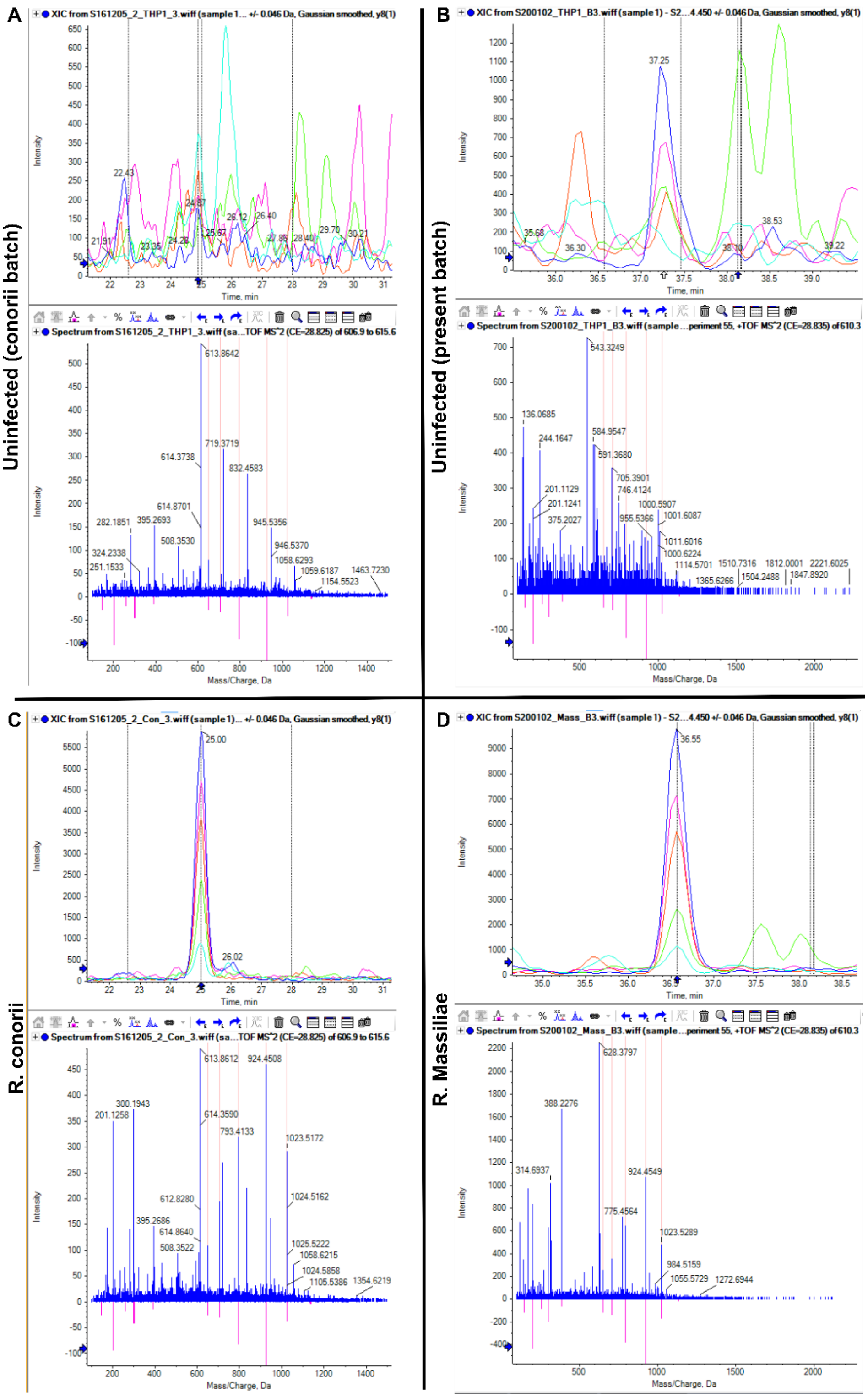

Fig S21. Extracted ion chromatograms (XIC) of IL-1β peptide. (A-B) XIC and spectrum of IL-1β peptide in uninfected THP-1 samples from *R. conorii* batch and present batch, respectively; **(C-D)** XIC and spectrum of IL-1β peptide in *R. conorii* and *R. massiliae* samples, respectively. XIC window for *R. conorii* batch samples of 10 min and 4 min for the present batch samples.

Interferon-induced GTP-binding protein Mx1 (SLPLLENQIK)

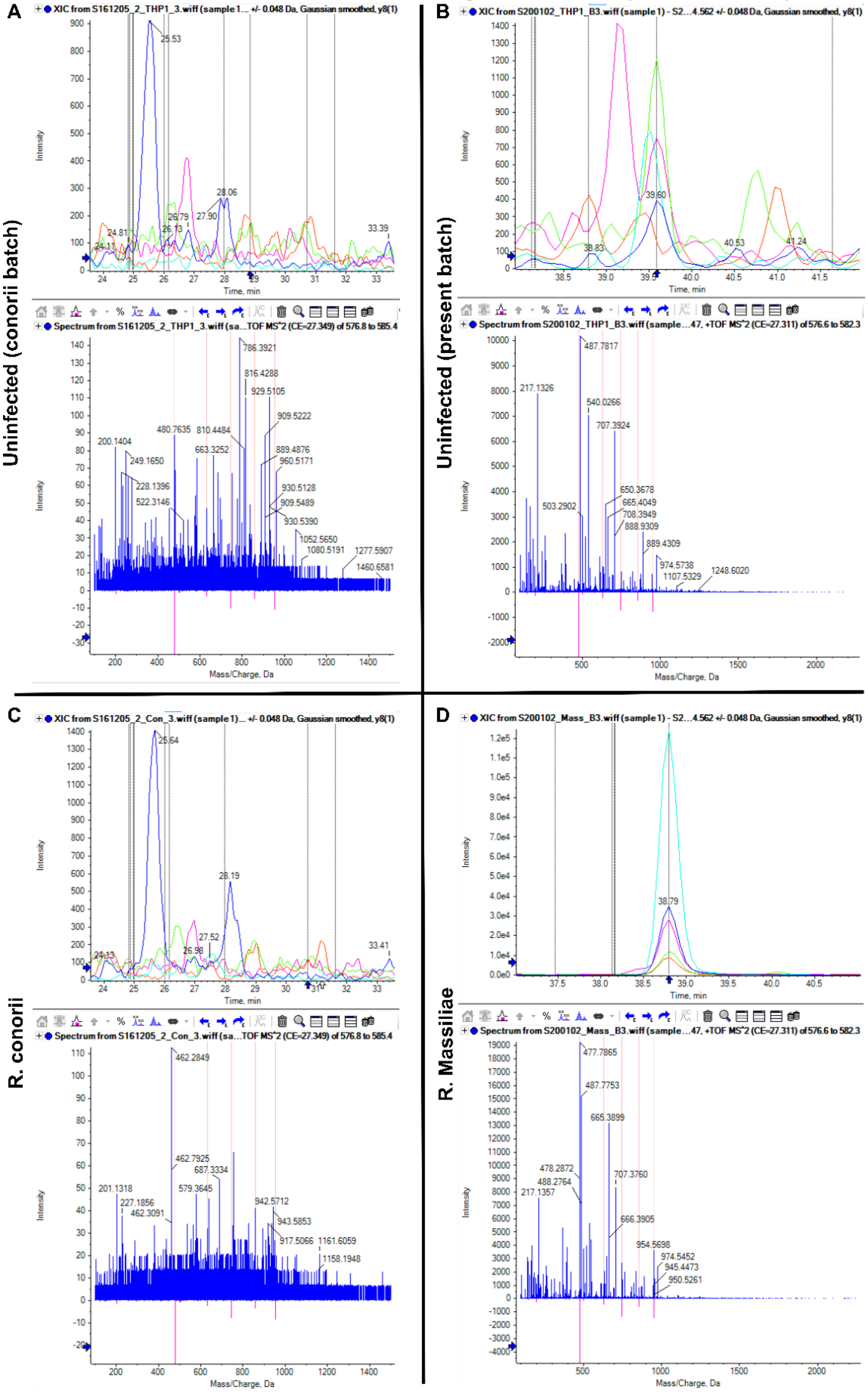

Fig S22. Extracted ion chromatograms of Interferon-induced GTP-binding protein Mx1. (A-B) XIC and spectrum of Mx1 in uninfected THP-1 samples from *R. conorii* batch and present batch, respectively; **(C-D)** XIC and spectrum of Mx1 peptide in *R. conorii* and *R. massiliae* samples, respectively. XIC window for *R. conorii* batch samples of 10 min and 4 min for the present batch samples.

Antiviral innate immune response receptor RIG-I (AAGFDEIEQDLTQR)

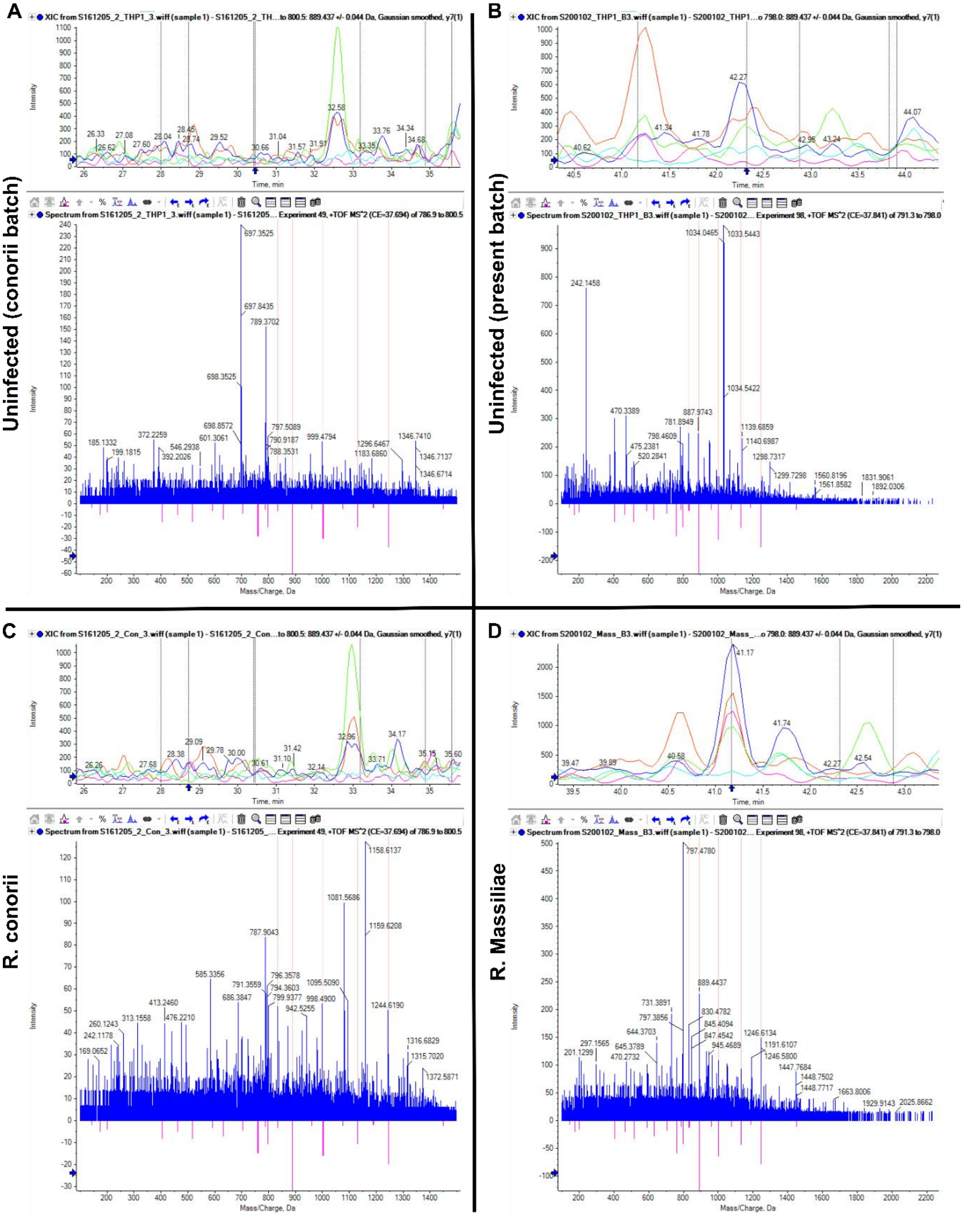

Fig S23. Extracted ion chromatograms of Antiviral innate immune response receptor RIG-I. (A-B) XIC and spectrum of RIG-I in uninfected THP-1 samples from *R. conorii* batch and

present batch, respectively; **(C-D)** XIC and spectrum of RIG-I peptide in *R. conorii* and *R. massiliae* samples, respectively. XIC window for *R. conorii* batch samples of 10 min and 4 min for the present batch samples.

Ubiquitin-like protein ISG15 (VPLASQGLGPGSTVLLVVDK)

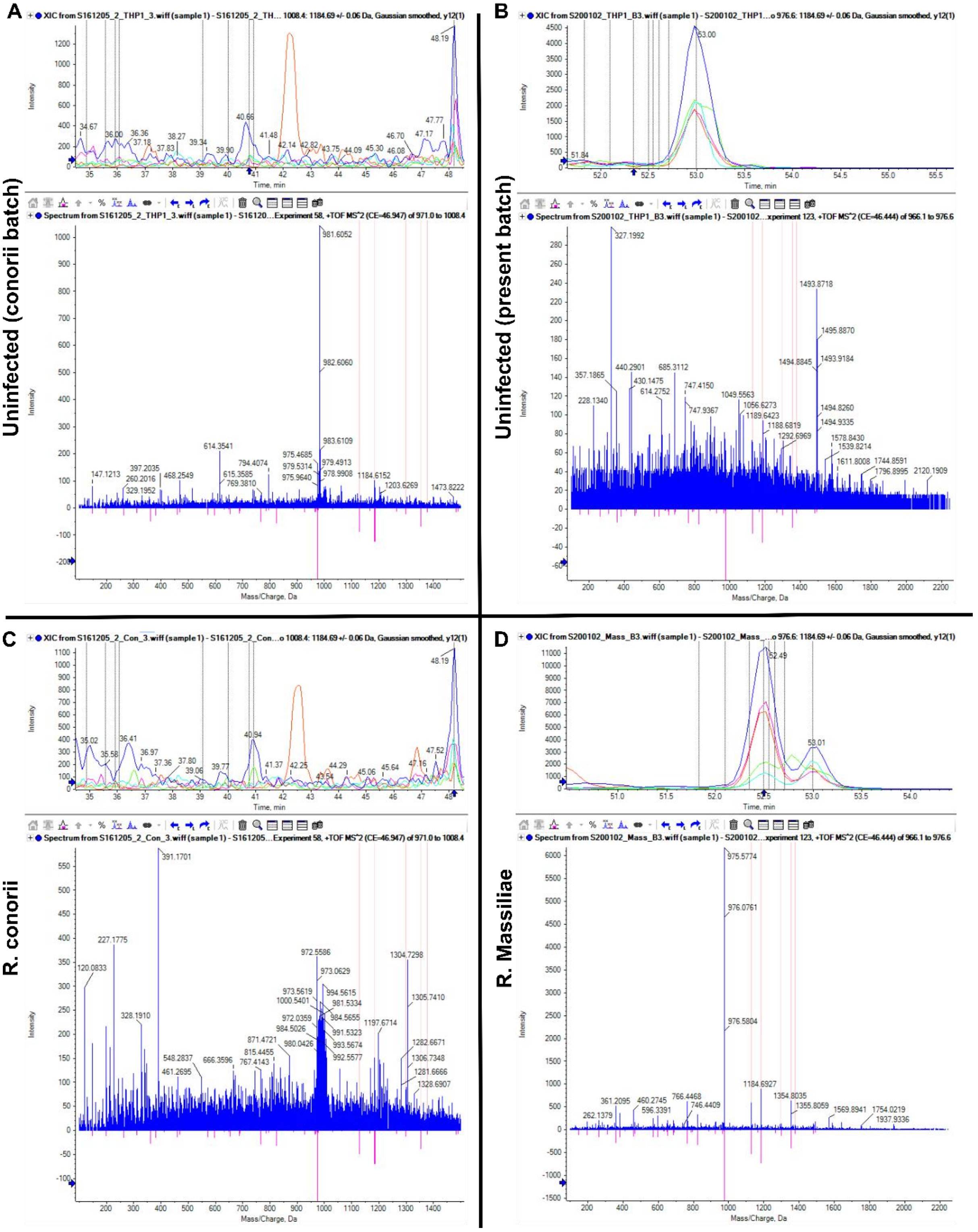

Fig S24. Extracted ion chromatograms of Ubiquitin-like protein ISG15. (A-B) XIC and spectrum of Ubiquitin-like protein ISG15 in uninfected THP-1 samples from *R. conorii* batch and

present batch, respectively; **(C-D)** XIC and spectrum of Ubiquitin-like protein ISG15 peptide in *R. conorii* and *R. massiliae* samples, respectively. XIC window for *R. conorii* batch samples of 14 min and 4 min for the present batch samples.

MalE-GFP (TWEEIPALDK)

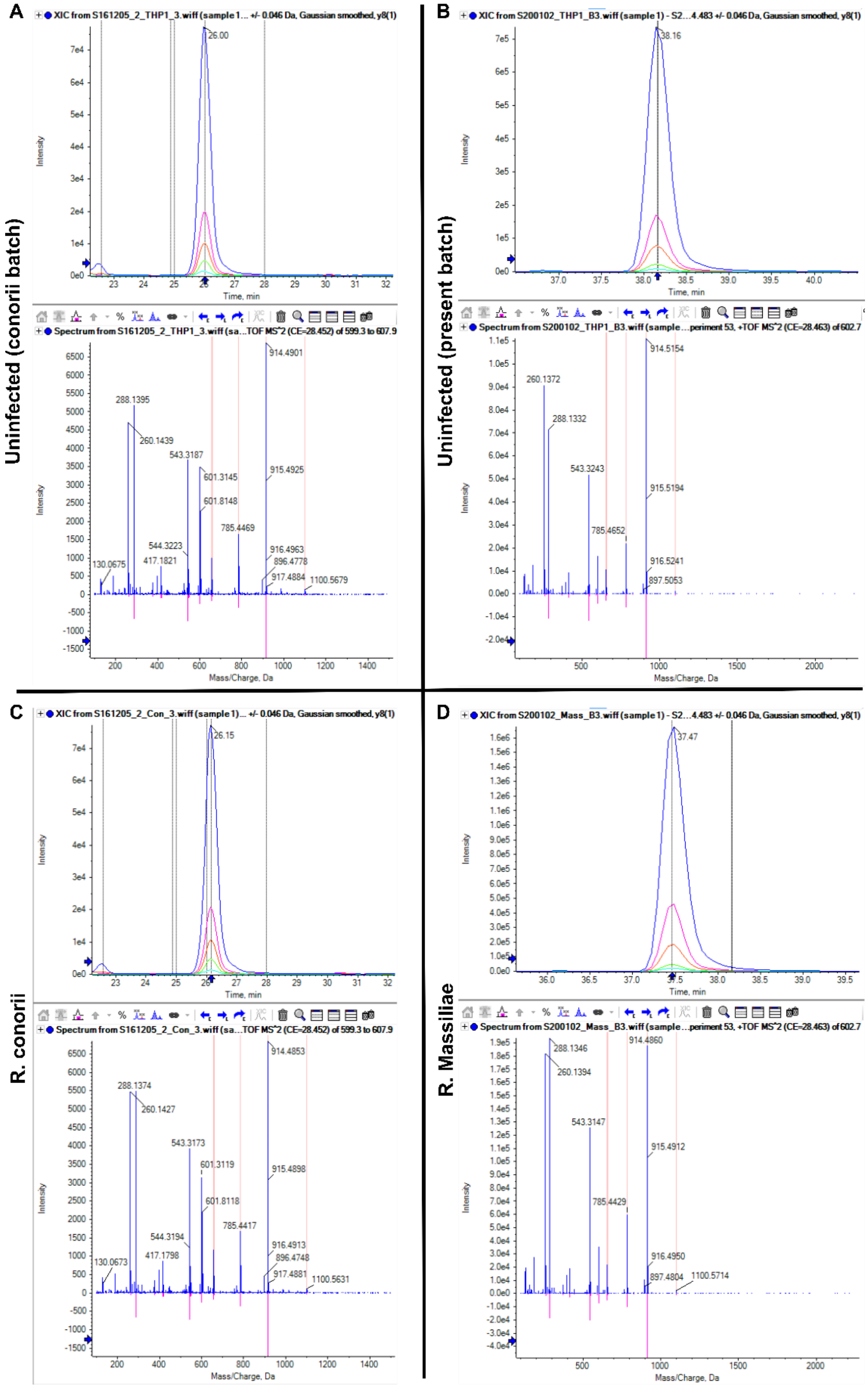

Fig S25. Extracted ion chromatograms of malE-GFP. (A-B) XIC and spectrum of malE-GFP in uninfected THP-1 samples from *R. conorii* batch and present batch, respectively; **(C-D)** XIC and spectrum of malE-GFP peptide in *R. conorii* and *R. massiliae* samples, respectively. XIC window for *R. conorii* batch samples of 10 min and 4 min for the present batch samples.

Myosin 9 (VISGVLQLGNIVFK)

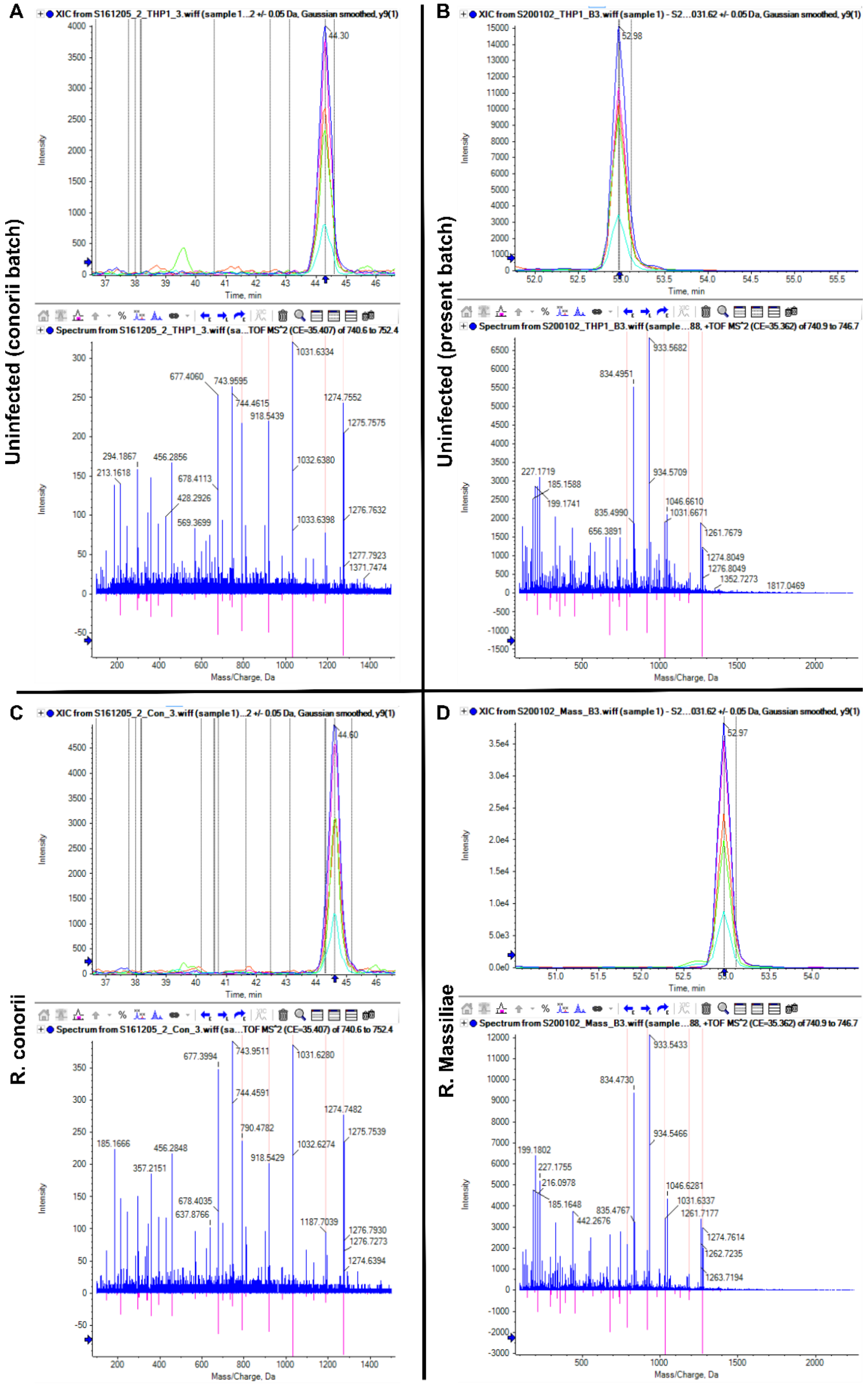

Fig S26. Extracted ion chromatograms of Myosin 9. (A-B) XIC and spectrum of myosin 9 in uninfected THP-1 sample from *R. conorii* batch and present batch, respectively; **(C-D)** XIC and spectrum of myosin 9 peptide in *R. conorii* and *R. massiliae* samples, respectively. XIC window for *R. conorii* batch samples of 10 min and 4 min for the present batch samples.

1. Curto P, Santa C, Allen P, Manadas B, Simoes I, Martinez JJ. 2019. A Pathogen and a Non-pathogen Spotted Fever Group Rickettsia Trigger Differential Proteome Signatures in Macrophages. Front Cell Infect Microbiol 9:43.
